# Terpenoids from the Soft Coral *Sinularia densa* Collected in the South China Sea

**DOI:** 10.3390/md22100442

**Published:** 2024-09-27

**Authors:** Cili Wang, Jiarui Zhang, Kai Li, Junjie Yang, Lei Li, Sen Wang, Hu Hou, Pinglin Li

**Affiliations:** 1 Key Laboratory of Marine Food Processing & Safety Control, College of Food Science and Engineering, Ocean University of China, Qingdao 266003, China; wangcili881@163.com; 2 Key Laboratory of Marine Drugs, Chinese Ministry of Education, School of Medicine and Pharmacy, Ocean University of China, Qingdao 266003, China; zjjrrracpt@163.com (J.Z.); 17854232177@163.com (K.L.); yangjunjie_ouc@163.com (J.Y.); wangsen19972021@163.com (S.W.); 3 Laboratory of Marine Drugs and Biological Products, National Laboratory for Marine Science and Technology, Qingdao 266235, China; 4 Biology Institute, Qilu University of Technology (Shandong Academy of Sciences), Jinan 250103, China; ll10610602375@163.com

**Keywords:** *Sinularia densa*, soft coral, terpenoids, anti-inflammatory activity, anti-thrombotic activity

## Abstract

The chemical investigation of the South China Sea soft coral *Sinularia densa* has resulted in the isolation of seven new terpenoids, including two new meroterpenoids, namely sinudenoids F–G (**1**–**2**), and five new cembranes, namely sinudenoids H–L (**3**–**7**). Their structures and absolute configurations were elucidated based on extensive analyses of spectroscopic data, single-crystal X-ray diffraction, comparison with the literature data, and quantum chemical calculations. Among them, sinudenoid F (**1**) and sinudenoid G (**2**) are rare meroterpenoids featuring a methyl benzoate core. Sinudenoid H (**3**) possesses a rare carbon skeleton of 8, 19-bisnorfuranocembrenolide, which is the second reported compound with this skeleton. In a bioassay, sinudenoid H (**3**) exhibited better anti-inflammatory activity compared to the positive control indomethacin at 20 µM in CuSO_4_-treated transgenic fluorescent zebrafish. Moreover, sinudenoid J (**5**) and sinudenoid L (**7**) exhibited moderate anti-thrombotic activity in arachidonic acid (AA)-induced thrombotic zebrafish at 20 µM.

## 1. Introduction

Soft corals of the genus *Sinularia* (order Malacalcyonacea, family Sinulariidae) are one of the most widely distributed soft coral genera across the oceans. Since the first report of this genus in 1975 [1], more than 150 species of this genus have been identified, and more than 70 species have been chemically investigated [2]. As an important source of marine-derived natural products, the genus has yielded over 700 metabolites, such as diterpenes [3], norditerpenes [4], sesquiterpenes [5], steroids [6], and other types [7,8]. The majority of secondary metabolites isolated from the genus *Sinularia* are terpenoid derivatives [2], and have exhibited diverse biological activities, including anti-inflammatory [9], cytotoxic [10], antifouling [11], antibacterial [12], and anti-HBV activities [13]. The genus *Sinularia* deserves further investigation due to its chemical diversity and extensive biological activities.

**Figure 1:**
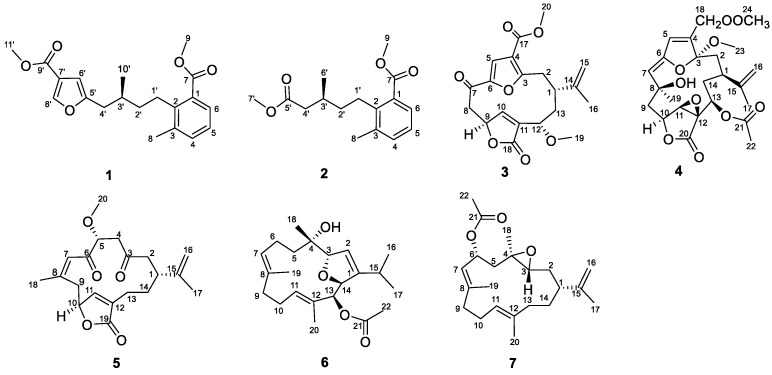
Structures of **1**–**7** from the soft coral *Sinularia densa*.

In our recent chemical investigations of the Xisha soft coral *Sinularia densa*, which is the first systematic investigation for this genus, five C_19_-Norcembranoid diterpenes with unusual scaffolds, named sinudenoids A–E, were discovered, showing the chemical diversity of this genus [14]. In order to find more bioactive metabolites, our ongoing research on the soft coral Xisha from *Sinularia densa* (Sinulariidae) has uncovered seven undescribed substances (**1**–**7**) (Figure 1). Here, we report the isolation, structure elucidation, and anti-inflammatory and anti-thrombotic effects of these isolated compounds.

## 2. Results

Sinudenoid F (**1**) was isolated as a colourless oil, and its molecular formula was determined as C_20_H_24_O_5_ by the HRESIMS ion peak at *m*/*z* 367.1511 [M + Na]^+^, implying nine degrees of unsaturation. Analysis of the ^1^H and ^13^C NMR data of **1** (Table S1) revealed the presence of 20 carbons, including seven non-protonated carbons (five olefinic and two carbonyl), six methines (one sp^3^ hybridized and five olefinic), three methylenes (all sp^3^ hybridized), and four methyls (two linked to oxygen, one to a sp^3^ carbon and one to an olefinic carbon).

**Figure 2:**
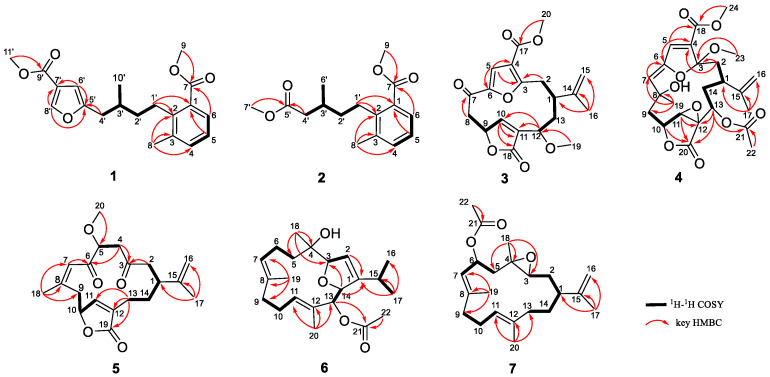
Selected ^1^H–^1^H COSY and HMBC correlations of **1**–**7**.

**Figure 3:**
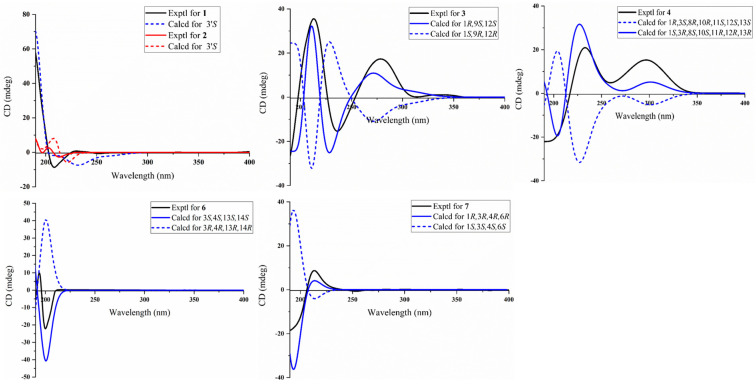
Experimental and calculated ECD spectra of **1**–**4** and **6**–**7**.

The presence of an isopentyl group was confirmed by the ^1^H–^1^H COSY correlations from H-1′ to H-4′ and H-3′ to H_3_-10′ (Figure 2). Subsequently, the presence of a trisubstituted benzene ring was supported by the HMBC correlations from H_3_-8 to C-2 (*δ*_C_142.0), C-3 (*δ*_C_137.4), and C-4 (*δ*_C_133.9); H-5 to C-1; and H-6 to C-7, together with the ^1^H–^1^H COSY correlation from H-4 to H-6. Furthermore, the HMBC correlation from H_3_-9 to C-7 (*δ*_C_169.0) indicated the presence of a methyl ester group at C-1 of the aromatic ring. Considering the aforementioned data, the HMBC correlations from H-1′ to C-1 and C-2 indicated the attachment of the isopentyl group to the trisubstituted benzene ring at C-2. Moreover, the presence of a furan ring located at C-4′ was inferred by the HMBC correlations from H-8′ to C-5′ and C-7′; H-6′ to C-5′ and C-7′; and H-4′ to C-5′, together with the downfield chemical shifts of C-5′ (*δ*_C_157.0) and C-8′ (*δ*_C_146.4). The HMBC correlation from H_3_-11′ to C-9′ (*δ*_C_164.1) indicated the presence of a methyl ester group, which was further confirmed by its molecular formula and the requirement of degrees of unsaturation. Due to the absence of the key HMBC correlation from either H-6′ to C-9′ or H-8′ to C-9′, the connection between C-7′ and C-9′ was established through a meticulous comparison of the 1D NMR data between **1** and 3-furancarboxylic acid [15], in conjunction with the absence of a substituent at C-7′. Consequently, the planer structure of **1** was established. Finally, the absolute configuration of **1** was defined as 3′*S* in the TDDFT-ECD calculations (Figure 3).

Sinudenoid G (**2**), a colourless oil, had a molecular formula of C_16_H_22_O_4_, as established by the HRESIMS ion peak at *m*/*z* 301.1412 [M + Na]^+^, which was 66 mass units less than that of **1**. The ^1^H and ^13^C NMR data of **2** (Table S2) resemble those of **1**, with the exception of the disubstituted furan ring at C-4′ in **1** replaced by a methyl ester group in **2**. This alteration was confirmed by the HMBC correlations (Figure 2) from H_3_-7′ to C-5′ (*δ*_C_173.6) and H-4′ to C-5′. Finally, the absolute configuration of **2** was defined as 3′*S* in the TDDFT-ECD calculations (Figure 3).

**Figure 4:**
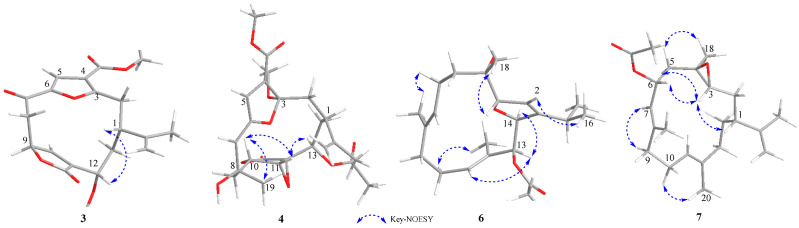
NOESY correlations of **3**–**4** and **6**–**7**.

Sinudenoid H (**3**) was isolated as a colourless oil, with a molecular formula of C_20_H_22_O_7_ deduced from the HRESIMS ion peak at *m*/*z* 375.1448 [M + H]^+^, implying ten degrees of unsaturation. The 1D NMR data of **3** (Table S3) resemble those of sarcofuranocembrenolide A, a known 8, 19-bisnorfuranocembrenolide previously isolated from the soft coral *Sarcophyton* sp. [16]. In fact, the structure of **3** closely resembles sarcofuranocembrenolide A, differing in the presence of a methoxy group at C-12 in **3** instead of an ethoxy group in sarcofuranocembrenolide A (Figure S1). The HMBC correlation (Figure 2) from H_3_-19 (*δ*_H_ 3.19) to C-12 confirmed these functional group disparities. In the NOESY spectrum of **3** (Figure 4), the correlation of H-12 (*δ*_H_ 4.08)/H-1 (*δ*_H_ 2.43) suggested their co-facial orientation. Due to the absence of definitive NOESY correlations, the configuration of C-9 was determined as S* using the DP4^+^ calculation (Supporting Information, Figures S2 and S3). Moreover, the TDDFT/ECD calculations also supported the 1*R*, 9*S*, 12*S*-configurations of **3** (Figure 3), instead of the 1*R*, 9*R*, 12*S* configurations (Figure S10).

Sinudenoid I (**4**) was isolated as a colourless oil. Its molecular formula was determined to be C_24_H_30_O_10_ from the sodium adduct ion peak at *m*/*z* 501.1729 [M + Na]^+^, implying ten degrees of unsaturation. Analysis of the 1D NMR data (Table S4) and HSQC spectrum of **4** suggested the presence of twenty-four carbons, including nine non-protonated carbons (three olefinic, three carbonyl, and three oxygenated), six methines (one sp^3^ hybridized, two olefinic, and three oxygenated), four methylenes (three sp^3^ hybridized and one olefinic), and five methyls (one linked to a carbonyl, one to an oxygenated sp^3^ carbon, two to oxygen atoms, and one to an olefinic carbon). These dates indicated that **4** was a polycyclic diterpenoid.

Upon scrutinizing the NMR data of **4**, it was found that the 1D NMR data resemble that of sinumaximol C, a diterpenoid previously isolated from the soft coral *Sinularia maxima* [17]. In fact, the structure of **4** was truly similar to sinumaximol C (Figure S1), except for the presence of an additional acetoxy group at C-13 in compound **4** in comparison to sinumaximol C. This deduction was further proven by HMBC correlations (Figure 2) from H_3_-22 (*δ*_H_ 2.01) to C-21 (*δ*_C_ 170.7), and H-13 (*δ*_H_ 4.84) to C-21. The 1D NOE correlations (Figure 4) from H-10 to H-11, H_3_-19, along with the NOESY correlation between H-13 and H-11, indicated that H-10, H-11, H-13, and H-19 were positioned on the same face. Subsequently, the similar chemical shifts of carbons from C-3 to C-12 compared with sinumaximol C indicated the 8R*, 10R*, 11S*, and 12S* configurations in **4**, which was same as those of sinumaximol C. In the absence of definitive NOESY correlations, the relative configurations of C-1 and C-3 were determined as 1R*, 3S* by the DP4^+^ calculation (Supporting Information, Figures S4 and S5). Finally, the absolute configurations of **4** were defined as 1*S*, 3*R*, 8*S*, 10*S*, 11*R*, 12*R*, and 13*R* using the TDDFT-ECD calculations (Figure 3).

**Figure 5:**
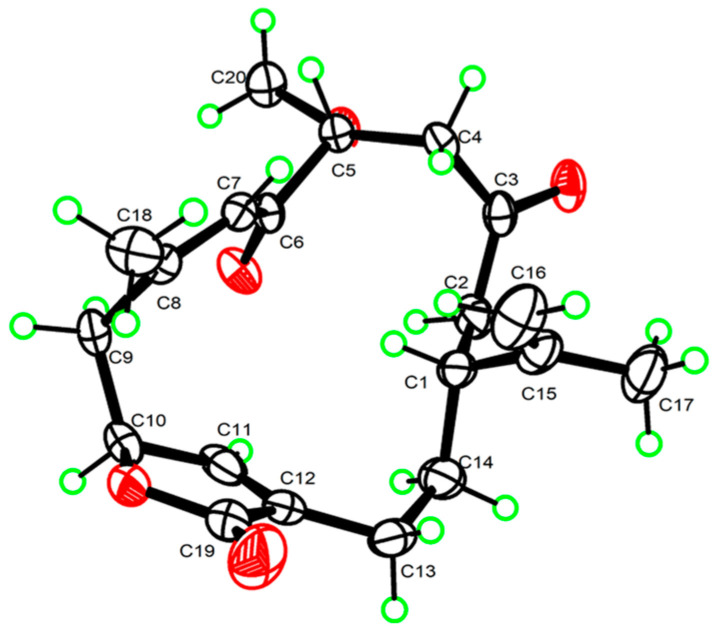
Perspective ORTEP drawings of the X-ray structures of **5** (displacement ellipsoids are drawn at the 50% probability level).

Sinudenoid J (**5**) was purified as a colourless crystal, with a molecular formula of C_20_H_26_O_5_ determined from its HRESIMS ion peak at *m*/*z* 369.1674 [M + Na]^+^. The ^1^H and ^13^C NMR data (Table S5) of **5** resemble those of gyrosanolide D (Figure S1), a known cembranoid previously isolated from the soft coral *Sinularia gyrosa* [18]. The difference in the planar structures between **5** and gyrosanolide D was that the hydroxy group at C-5 in gyrosanolide D was replaced by a methoxy group in **5**. This deduction was further proven by the HMBC correlation (Figure 2) from H_3_-20 (*δ*_H_ 3.41) to C-5 (*δ*_C_ 82.6). Due to the lack of effective NOESY correlations, the geometry of the double bond and the configurations of chiral centres in compound **5** could not be definitively determined. However, an X-ray crystallographic experiment with the Cu Kα radiation of **5** (Figure 5) confirmed the *Z*-geometry of the Δ^7^ double bond. Subsequently, the absolute configurations of **5** were defined as 1*R*, 5*R*, and 10*S*, indicating that the geometry of the Δ^7^ double bond and the configuration at C-5 in **5** differ from those of gyrosanolide D.

Sinudenoid K (**6**) was isolated as a colourless oil. Its molecular formula of C_22_H_32_O_4_ was deduced from the sodium adduct ion peak at *m*/*z* 361.2374 [M + H]^+^, indicating seven degrees of unsaturation. Analysis of the 1D NMR data (Table S6) and HSQC spectrum of **6** revealed the presence of twenty-two carbons, including five non-protonated carbons (three olefinic, one carbonyl, and sp^3^ hybridized), seven methines (one sp^3^ hybridized, three olefinic, and three oxygenated), four methylenes (all sp^3^ hybridized), and six methyls (one linked to a carbonyl, one to an oxygenated sp^3^ carbon, two to sp^3^ carbons and two to olefinic carbons). These dates indicated that **6** possessed a cembrane nucleus.

Upon analysis the NMR data of **6**, it was found that the 1D NMR data of **6** resemble that of klyflaccicembranols H, a cembranoid previously isolated from the soft coral *Klyxum flaccidum* [19]. In fact, the structure of **6** was truly similar to that of klyflaccicembranol H (Figure S1), except for the presence of the trisubstituted double bond at C-11, C-12, and C-20 in **6,** instead of a trisubstituted epoxide ring in klyflaccicembranol H. This deduction was further proven by the HMBC correlations (Figure 2) from the H_3_-20 (*δ*_H_ 1.53) to C-11 (*δ*_C_ 133.3), C-12 (*δ*_C_ 129.2), and C-13 (*δ*_C_ 81.2).

In the NOESY spectrum of **6** (Figure 4), the correlations of H_3_-19/H-6a (*δ*_H_ 2.42), H-11/H_3_-13, and H_3_-20/H-10a (*δ*_H_ 2.29) indicated that the Δ^7^ and Δ^11^ double bonds exhibited *E*-geometry. The correlation of H-2/H_3_-16 (*δ*_H_ 1.16) suggested that the Δ^2^ double bond had a *Z*-geometry. Additionally, the NOESY cross-peak of H_3_-18/H-3 suggested that these protons were co-facial. Furthermore, the correlation from H-14 to H-13 in the 1D-NOE difference spectrum indicated that H-13 and H-14 were on the same side. In the absence of the key NOESY correlations, the relative configurations of these chiral centres were determined as 3S*, 4S*, 13S*, and 14S* using the DP4^+^ calculation (Supporting Information, Figures S6 and S7). Finally, the absolute configurations of **6** were defined through the TDDFT-ECD calculations (Figure 3).

Sinudenoid L (**7**) was isolated as a colourless oil, with a molecular formula of C_22_H_34_O_3_, as indicated by its HRESIMS ion peak at *m*/*z* 369.2399 [M + Na]^+^. The 1D NMR data of **7** (Table S7) resemble those of (7*E*,11*E*)-3,4-epoxy-7,11,15-cembratriene (Figure S1), a cembranoid previously isolated from a south Pacific soft coral [20]. The only difference was the replacement of one hydrogen atom of methylene at C-6 in the known cembranoid with an acetoxy group in **7**. The differentiation was supported by the HMBC correlations (Figure 2) from H_3_-22 to C-21 (*δ*_C_ 170.1) and H-6 (*δ*_H_ 5.66) to C-21.

Then, the relative configurations of **7** were determined by the analysis of the NOESY spectrum (Figure 4). The NOESY correlations of H-7/H-9a (*δ*_H_ 1.98) and H_3_-20/H-10a (*δ*_H_ 2.25) were used to establish the *E*-geometry of the Δ^7^ and Δ^11^ double bonds. Additionally, the NOESY correlations of H-1/H-3 and 5a (*δ*_H_ 1.45)/H-3 indicated that these protons were all co-facial. Moreover, the NOESY correlations of H-5b/H_3_-18 indicate these protons were also co-facial. By utilizing the ^13^C NMR chemical shift calculations for the DP4^+^ calculations (Supporting Information, Figures S8 and S9), in conjunction with the NOESY correlation of H-3/H-6, the configuration of C-6 was defined as R*. Finally, the TDDFT/ECD calculations of **7** also supported the 1*R*, 3*R*, 4*R*, and 6*R*-configurations (Figure 3), instead of the 1*R*, 3*R*, 4*R*, and 6*S*-configurations (Figure S11).

**Figure 6:**
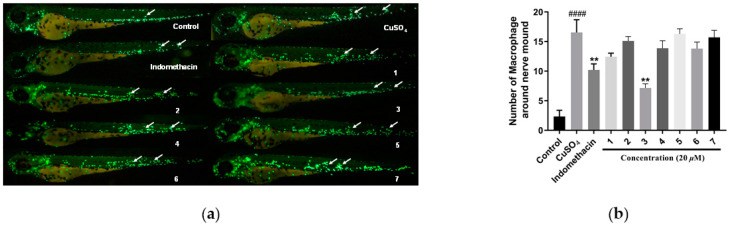
Anti-inflammatory assays of **1**–**7**. (**a**) Images of inflammatory sites in CuSO_4_-treated transgenic fluorescent zebrafish (Tg:zlyz-EGFP) expressing enhanced green fluorescent protein (EGFP) treated with **1** through **7**, using indomethacin as a positive control. (**b**) Quantitative analysis of macrophages in the region of inflammatory sites in zebrafish treated with **1** through **7**. #### indicates that the CuSO_4_ model group has a very significant difference compared with the control group (*p* < 0.01). ** indicates that sample groups have significant differences compared with the CuSO_4_ model group (*p* < 0.01).

**Figure 7:**
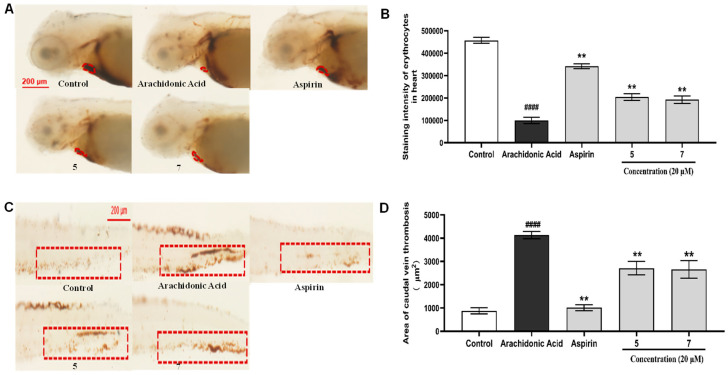
Anti-thrombotic assays of **5** and **7**. (**A**) Images of the staining intensity of erythrocytes in the heart in arachidonic acid (AA)-induced thrombotic zebrafish, treated with either **5** or **7**, using aspirin as a positive control. (**B**) Quantitative analysis of the staining intensity of erythrocytes in the heart in zebrafish treated with either **5** or **7**. (**C**) Images of areas of caudal vein thrombosis in arachidonic acid (AA)-induced thrombotic zebrafish, treated with either **5** or **7**, using aspirin as a positive control. (**D**) Quantitative analysis of the area of caudal vein thrombosis in zebrafish treated with either **5** or **7**. #### indicates that the arachidonic acid model group has a very significant difference compared with the control group (*p* < 0.01). ** indicates that sample groups have significant differences compared with the arachidonic acid model group (*p* < 0.01).

In the bioassay, these new compounds (**1**–**7**) were evaluated for anti-inflammatory activity in CuSO_4_-treated transgenic fluorescent zebrafish. The results (Figure 6) showed that **3** could reduce migration and decrease the number of macrophages surrounding the neuromasts in zebrafish, displaying better anti-inflammatory activity with an inhibition rate of 56.8% compared to the indomethacin positive control with an inhibition rate of 38.4% at 20 μM. Moreover, due to the limited availability, **5** and **7** were selectively evaluated for anti-thrombotic activity in arachidonic acid (AA)-induced thrombotic zebrafish. In arachidonic acid (AA)-induced thrombotic zebrafish, **5** and **7** enhanced the staining intensity of erythrocytes in the heart and inhibited the area of caudal vein thrombosis, demonstrating anti-thrombotic activity at 20 μM (Figure 7).

## 3. Materials and Methods

### 3.1. General Experimental Procedures

Optical rotations were measured using a Jasco P-1020 digital polarimeter. Ultraviolet (UV) spectra were measured using a Beckman DU640 spectrophotometer. CD spectra were measured using a Jasco J-810 spectropolarimeter. NMR spectra were measured using an Agilent 500 MHz and a JEOL JNMECP 600 spectrometer. The 7.26 ppm (^1^H) resonance of the residual CHCl_3_ in CDCl_3_ and the 77.16 ppm (^13^C) resonance of CDCl_3_ were used as internal references for ^1^H and ^13^C NMR spectra, respectively. HRESIMS spectra were measured using a Micromass Q-Tof Ultima GLOBAL GAA076LC mass spectrometer. The crystallographic data were measured on a Bruker D8 Venture diffractometer (Bruker, Beijing, China) equipped with graphite-monochromatized Cu Kα radiation. A semi-preparative HPLC was performed using a Waters 1525 pump equipped with a 2998 photodiode array detector and a YMC C18 column (YMC, 10 × 250 mm, 5 μm). Silica gel (200–300 mesh, 300–400 mesh and silica gel H) was used for the column chromatography.

### 3.2. Animal Material

The soft coral *Sinularia densa* (Sinulariidae) was collected from Xisha Island (Ganquan island) of the South China Sea in July 2018 (111°58′ E, 16°50′ N), and was frozen immediately after collection. The specimen was dispatched via express delivery and subsequently identified based on its morphology and sclerites by Prof Ping-Jyun Sung, Institute of Marine Biotechnology, National Museum of Marine Biology & Aquarium, Pingtung 944, Taiwan. The voucher specimen (No. xs-18-yg-99) has been deposited at the State Key Laboratory of Marine Drugs, Ocean University of China, People’s Republic of China.

### 3.3. Extraction and Isolation

A frozen specimen of *Sinularia densa* (3.5 kg, wet weight) was homogenized and then exhaustively extracted with CH_3_OH six times (for 3 days each time) at room temperature. The combined solutions were concentrated in vacuo and desalted by redissolving with CH_3_OH to yield a residue (83.0 g). The crude extract was subjected to silica gel vacuum column chromatography eluted with a gradient of petroleum ether/acetone (from 200:1 to 1:1, *v*/*v*) and subsequently CH_2_Cl_2_/MeOH (from 10:1 to 1:1, *v*/*v*) to obtain fourteen fractions (Fr.1–Fr.14). Each fraction was detected by TLC. Fr.2 was subjected to silica gel vacuum column chromatography (petroleum ether/acetone, from 50:1 to 1:1, *v*/*v*) to give four subfractions, Fr.2.1–Fr.2.4. Fr.2.3 was separated by semi-preparative HPLC (ODS, 5 µm, 250 × 10 mm; MeOH/H_2_O, 85:15, *v*/*v*; 1.5 mL/min) to afford **1** (6.3 mg, *t*_R_ = 24 min). Fr.2.4 was separated by semi-preparative HPLC (ODS, 5 µm, 250 × 10 mm; MeOH/H_2_O, 85:15, *v*/*v*; 1.5 mL/min) to afford **2** (1.4 mg, *t*_R_ = 15 min). Fr.4 was subjected to silica gel vacuum column chromatography (petroleum ether/acetone, from 40:1 to 1:1, *v*/*v*) to give four subfractions, Fr.4.1–Fr.4.4. Fr.4.3 was separated by semi-preparative HPLC (ODS, 5 µm, 250 × 10 mm; MeOH/H_2_O, 65:35, *v*/*v*; 1.5 mL/min) to afford **3** (1.0 mg, *t*_R_ = 42 min) and **6** (2.4 mg, *t*_R_ = 48 min). Fr.6 was subjected to silica gel vacuum column chromatography (petroleum ether /acetone, from 30:1 to 1:1, *v*/*v*) to give six subfractions, Fr.6.1–Fr.6.6. Fr.6.4 was separated by semi-preparative HPLC (ODS, 5 µm, 250 × 10 mm; MeOH/H_2_O, 45:55, *v*/*v*; 1.5 mL/min) to afford **5** (2.2 mg, *t*_R_ = 48 min) and **4** (3.0 mg, *t*_R_ = 30 min). Fr.6.5 was separated by semi-preparative HPLC (ODS, 5 µm, 250 × 10 mm; MeOH/H_2_O, 45:55, *v*/*v*; 1.5 mL/min) to afford **7** (2.3 mg, *t*_R_ = 48 min).

Sinudenoid **F** (**1**): Colourless oil; [α]^25^_D_ +85.2 (*c* 0.1, MeOH); UV (MeOH) λmax (log ε) = 200 (2.22) nm, 233 (0.36) nm; IR (KBr) *ν*_max_ = 3420, 2980, 1740, 1635, 1033 cm^−1^; HRESIMS m/z 345.1693 [M + H]^+^ (calcd. for C_20_H_25_O_5_, 345.1697) and HRESIMS *m*/*z* 367.1511 [M + Na]^+^ (calcd. for C_20_H_24_O_5_Na, 367.1516). For ^1^H NMR and ^13^C NMR data, see Table S1.

Sinudenoid **G** (**2**): Colourless oil; [α]^25^_D_ −32.1 (*c* 0.1, MeOH); UV (MeOH) λmax (log ε) = 204 (1.20) nm, 242 (0.40) nm; IR (KBr) *ν*_max_ = 2924, 1719, 1615, 1384, 1269 cm^−1^; HRESIMS *m*/*z* 279.1594 [M + H]^+^ (calcd. for C_16_H_23_O_4_, 279.1591) and HRESIMS *m*/*z* 301.1412 [M + Na]^+^ (calcd. for C_16_H_22_O_4_Na, 301.1410). For ^1^H NMR and ^13^C NMR data, see Table S2.

Sinudenoid **H** (**3**): Colourless oil; [α]^25^_D_ +35.2 (*c* 0.5, MeOH); UV (MeOH) λmax (log ε) = 193 (1.50) nm, 280 (0.69) nm; IR (KBr) *ν*_max_ = 3420, 1756, 1718, 1683, 1592, 1384 cm^−1^; HRESIMS *m*/*z* 375.1448 [M + H]^+^ (calcd. for C_20_H_23_O_7_, 375.1438). For ^1^H NMR and ^13^C NMR data, see Table S3.

Sinudenoid **I** (**4**): Colourless oil; [α]^25^_D_ −124.0 (*c* 1.0, MeOH); UV (MeOH) λmax (log ε) = 192 (0.84) nm, 300 (0.92) nm; IR (KBr) *ν*_max_ = 2925, 1750, 1708, 1670, 1645, 1363 cm^−1^; HRESIMS *m*/*z* 501.1729 [M + Na]^+^ (calcd. for C_24_H_30_O_10_Na, 501.1731). For ^1^H NMR and ^13^C NMR data, see Table S4.

Sinudenoid **J** (**5**): Colourless crystal; [α]^25^_D_ +67.0 (*c* 0.3, MeOH); UV (MeOH) λmax (log ε) = 194 (0.89) nm; IR (KBr) *ν*_max_ = 1757, 1734, 1716 cm^−1^; HRESIMS *m*/*z* 347.1859 [M + H]^+^ (calcd. for C_20_H_27_O_5_, 347.1853). For ^1^H NMR and ^13^C NMR data, see Table S5.

Sinudenoid **K** (**6**): Colourless oil; [α]^25^_D_ −29.0 (*c* 0.5, MeOH); UV (MeOH) λmax (log ε) = 192 (0.04) nm, 214 (0.04) nm, 240 (0.03) nm; IR (KBr) *ν*_max_ = 3431, 2359, 1631, 1375 cm^−1^; HRESIMS *m*/*z* 361.2374 [M + H]^+^ (calcd. for C_22_H_33_O_4_, 361.2384). For ^1^H NMR and ^13^C NMR data, see Table S6.

Sinudenoid **L** (**7**): Colourless oil; [α]^25^_D_ +28.3 (*c* 0.1, MeOH); UV (MeOH) λmax (log ε) = 195 (1.68) nm; IR (KBr) *ν*_max_ = 3419, 2925, 1683, 1615, 1384 cm^−1^; HRESIMS *m*/*z* 369.2399 [M+Na]^+^ (calcd. for C_22_H_34_O_3_Na, 369.2400). For ^1^H NMR and ^13^C NMR data, see Table S7.

### 3.4. X-ray Crystallographic Analysis

Sinudenoid J (**5**) was obtained as a colourless crystal in an EtOH-H_2_O solvent system using the vapour diffusion method. Crystal data for Sinudenoid J (**5**): C_20_H_26_O_5_, M = 346.41, a = 7.4110(3) Å, b = 12.6917(5) Å, c = 19.7025(8) Å, α = 90°, β = 90°, γ = 90°, V = 1853.18 (13) Å^3^, T = 150 K, space group P2_1_2_1_2_1_, Z = 4, *μ* (Cu Kα) = 0.720 mm^−1^, 3717 independent reflections (R*int* = 0.0488). The final R_1_ values were 0.0356 (I > 2σ(I)). The final wR_2_ values were 0.0869 (I > 2σ(I)). The final R_1_ values were 0.0370 (all data). The final wR_2_ values were 0.0882 (all data). The goodness of fit on F^2^ was 1.044. The Flack parameter was −0.02(7). The crystallographic data for **5** in this article have been deposited at the Cambridge Crystallographic Data Centre under supplementary publication number 2150265. The data can be obtained via https://www.ccdc.cam.ac.uk/ (accessed on 5 February 2022).

### 3.5. Bioassay

Three-day post-fertilization (dpf) healthy macrophage fluorescent transgenic zebrafish were employed as animal models to evaluate the anti-thrombotic and anti-inflammatory effects of the isolates. Healthy macrophage fluorescent transgenic zebrafish (Tg: zlyz-EGFP) were provided by the Biology Institute of Shandong Academy of Sciences (Jinan, China). Zebrafish larvae with CuSO_4_-treated transgenic fluorescent (Tg:zlyz-EGFP) expressing enhanced green fluorescent protein (EGFP) were used to evaluate anti-inflammatory effects. Zebrafish larvae with arachidonic acid (AA)-induced thrombus were utilized to evaluate anti-thrombosis effects. Each zebrafish larva was imaged using a fluorescence microscope (AXIO, Zom.V16), and the number of macrophages around the nerve mound were calculated using Image-Pro Plus software (version 5.1). Statistical analysis was conducted using a one-way analysis of variance with GraphPad Prism 7.00 software.

## 4. Conclusions

In summary, two rare meroterpenoids (**1**–**2**) and five new cembranes (**3**–**7**) were isolated from the soft coral *Sinularia densa*. Sinudenoid F (**1**) and sinudenoid G (**2**) are rare meroterpenoids featuring a methyl benzoate core. While the skeleton of **1** has been obtained in synthetic studies, it represents the first instance of this natural product skeleton [21]. Sinudenoid H (**3**) possesses a rare carbon skeleton of 8, 19-bisnorfuranocembrenolide, and it is the second reported compound of this skeleton [15]. Sinudenoid J (**5**) is a new furanobutenolide-derived C_19_-norcembranoid diterpene, whose structure was confirmed by X-ray diffraction analysis. In the biological assays, **3** showed potent anti-inflammatory activity, while **5** and **7** demonstrated moderate anti-thrombotic activity in zebrafish models. The current study shows the potential of the cembranes as marine-derived anti-inflammatory and anti-thrombotic lead molecules, and provides a basis for developing new drugs.

## Data Availability

The data are contained within the article or Supplementary Materials; further inquiries can be directed to the corresponding author.

## Supplementary Materials

The following supporting information can be downloaded at https://www.mdpi.com/article/10.3390/md22100442/s1, Tables S1–S7: NMR data of **1**–**7**; Figure S1: Structures of **3**–**7** and structurally related compounds of **3**–**7**; Tables S8–S11 and Figures S2–S9: The determination of the relative and absolute configurations for **3**, **4**, **6**, and **7**, respectively; Figures S10 and S11: Experimental and calculated ECD spectra of **3a** and **7b**, respectively; Table S12: Anti-inflammation assay of **1**–**7**; Table S13: Anti-thrombotic assay of **5** and **7**; Table S14 and Figures S12–S17: Computational details; Figures S18–S84: Spectra for **1**–**7**.
