# Supplementary material for: Terpenoids from the Soft Coral Sinularia densa Collected in the South China Sea
Source: Mar Drugs. 2024 Sep 27;22(10):442. doi: 10.3390/md22100442 (PMC11509852; doi:10.3390/md22100442)
Supplement: Supplementary file 1 [file marinedrugs-22-00442-s001.zip › marinedrugs-3208363-supplementary.pdf]

Supporting Information

Terpenoids from the soft coral *Sinularia densa* collected in the South China Sea

*Cili Wang [1,2,3], Jiarui Zhang [2,3], Kai Li [2,3], Junjie Yang [2,3], Lei Li [4], Sen Wang[2,3], Hu Hou [1,2,3,]*and Pinglin Li [2,3,]**

[1] State Key Laboratory of Marine Food Processing & Safety Control, College of Food Science and Engineering, Ocean University of China, Qingdao, 266003, China.

[2] Key Laboratory of Marine Drugs, Chinese Ministry of Education, School of Medicine and Pharmacy, Ocean University of China, Qingdao 266003, China.

[3] Laboratory of Marine Drugs and Biological Products, National Laboratory for Marine Science and Technology, Qingdao 266235, China.

[4]Biology Institute, Qilu University of Technology (Shandong Academy of Sciences), Jinan 250103, China

**Correspondence: houhu@ouc.edu.cn (H.H.); lipinglin@ouc.edu.cn (P.L.); Tel.: +86-532-6089-2936 (H.H.); +86-532-8203-3054 (P.L.)*

Table

1. NMR data assignments of 1–7 3
2. The Determination of the Relative and Absolute Configuration 11
3. Biological assays 21
4. The 1D and 2D NMR spectra of 1–7 43

1. NMR data assignments of 1–7

Table S1. 1D and 2D NMR Data of Sinudenoid F (**1**)

No.	**1**[a]			
	δ_C[b], type	δ_H[c] (*J* in Hz)	^1H-^1H COSY	HMBC
1	130.7, C			
2	142.0 C			
3	137.4, C			
4	133.9, CH	7.28, d, (11.0)	H-5	
5	125.6, CH	7.12, t, (9.5)	H-4, H-6	
6	128.3, CH	7.62, d, (9.5)	H-5	C-7
7	169.0, C			
8	19.8, CH_3	2.33, s		C-2, C-3, C-4
9	52.1, CH_3	3.86, s		C-7
1'a	28.1, CH_2	2.88, m	H-2'a, H-2'b	C-1, C-2
1'b		2.95, m	H-2'a, H-2'b	C-1, C-2
2'a	37.0, CH_2	1.58, m	H-1'a, H-1'b, H-3'	
2'b		1.43, m	H-1'a, H-1'b, H-3'	
3'	33.3, CH	1.96, m	H-2'a, H-2'b, H-4'a, H-4'b, H-10'	
4'a	35.2, CH_2	2.52, dd, (18.0, 8.5)	H-3'	C-5'
4'b		2.71, dd, (18.0, 8.5)	H-3'	C-5'
5'	157.1, C			
6'	106.1, CH	6.36, m		C-5', C-7'
7'	119.7, C			
8'	146.4, CH	7.88, s		C-5', C-7'
9'	164.1, C			
10'	19.5, CH_3	1.01, d, (8.0)	H-3'	
11'	51.6, CH_3	3.81, s		C-9'

[a] In chloroform-*d*. [b] Recorded at 125 MHz. [c]Recorded at 500 MHz.

Table S2. 1D and 2D NMR Data of sinudenoid G (**2**)

No.	2[a]			
	δ_C[b], type	δ_H[c] (*J* in Hz)	^1H-^1H COSY	HMBC
1	128.1, C			
2	141.7, C			
3	141.0, C			
4	130.4, CH	7.19, d, (8.0)	H-5	
5	127.3, CH	7.76, t, (10.0)	H-4, H-6	
6	129.9, CH	7.79, d, (10.0)	H-5	C-7
7	167.5, C			
8	19.6, CH_3	2.35, s		C-2, C-3, C-4
9	52.1, CH_3	3.90, s		C-7
1'a	30.8, CH_2	2.68, m	H-2'a, H-2'b	C-1, C-2
1'b		2.61, m	H-2'a, H-2'b	C-1, C-2
2'b	37.3, CH_2	1.61, m	H-1'a, H-1'b, H-3'	
2'b		1.48, m	H-1'a, H-1'b, H-3'	
3'	30.7, CH	2.08, m	H-2'a, H-2'b, H-4'a, H-4'b ,H-6'	
4'a	41.6, CH_2	2.22, m	H-3'	C-5'
4'b		2.40, m	H-3'	C-5'
5'	173.6, C			
6'	19.8, CH_3	1.04, d, (8.5)	H-3'	
7'	51.6, CH_3	3.68, s		C-5'

[a] In chloroform-*d*. [b] Recorded at 125 MHz. [c]Recorded at 500 MHz

Table S3. 1D and 2D NMR Data of sinudenoid H (**3**)

**No.**	**3**[a]				
	**δ_C[b], type**	**δ_H[c] (J in Hz)**	**^{1}H-^{1}H COSY**	**HMBC**	**NOEDY**
1	40.5, CH	2.43, m	H-2a, H-2b, H-13a, H-13b		
2a	32.2, CH_2	3.62, m	H-1	C-3	
2b		2.70, m	H-1	C-3	
3	164.1, C				
4	119.2, C				
5	118.0, CH	7.52, s		C-3, C-4, C-6	
6	149.8, C				
7	184.5, C				
8a	45.9, C	3.38, m	H-9	C-7	
8b		3.32, m	H-9	C-7	
9	76.9, CH	5.33, t, (8.5)	H-8a, H-8b, H-10		
10	152.3, CH_2	6.76, s	H-9	C-11, C-18	
11	135.5, C				
12	71.4, CH	4.08, dd, (12.1, 3.5)	H-13a, H-13b	C-10, C-11	H-1
13a	38.2, CH_2	2.07, m	H-1, H-12		
13b		1.16, m	H-1, H-12		
14	144.8, C				
15a	114.0, CH_2	4.95, s			
15b		4.98, s			
16	19.1, CH_3	1.83, s		C-1, C-14, C-15	
17	162.9, C				
18	172.6, C				
19	57.0, CH_3	3.19, s		C-12	
20	52.0, CH_3	3.88, s		C-17	

[a] In chloroform-*d*. [b] Recorded at 125 MHz. [c]Recorded at 500 MHz

Table S4. 1D and 2D NMR Data of sinudenoid I (**4**)

No.	4[a]				
	δ_C[b], type	δ_H[c] (*J* in Hz)	^{1}H-^{1}H COSY	HMBC	NOEDY/1D-NOE
1	36.3, CH	2.16, m	H-2a, H-2b, H-14a, H-14b		
2a	43.1, CH_2	2.47 dd, (18.5, 12.5)	H-1	C-3	
2b		1.84, dd, (18.5, 12.5)	H-1	C-3, C-4	
3	116.7, C				
4	130.7 C				
5	139.5, CH	7.14, s		C-4, C-6, C-18	
6	150.2, C				
7	117.9, CH	5.18, s		C-6	
8	71.5, C				
9a	42.0, CH_2	1.98, m	H-10		
9b		2.81, dd, (17.5, 3.0)	H-10		
10	73.8, CH	4.80, t, (7.5)	H-9, H-11	C-20	H-11, H-19
11	62.3, CH	3.88, s	H-10	C-12, C-20	
12	58.4, C				
13	71.1, CH	4.84, d, (8.5)	H-14a, H-14b	C-12, C-20, C-21	H-11
14a	37.3, CH_2	1.62, m	H-1, H-13		
14b		2.64, m	H-1, H-13		
15	147.3, C				
16a	112.7, CH_2	4.69, s			
16b		4.66, s			
17	18.4, CH_3	1.59, s		C-1, C-15, C-16	
18	162.2, C				
19	29.2, CH_3	1.54, s		C-7, C-8, C-9	
20	168.1, C				
21	170.7, C				
22	20.6, CH_3	2.01, s		C-21	
23	50.3, CH_3	3.09, s		C-3	
24	52.0, CH_3	3.73, s		C-18	

[a] In chloroform-*d*. [b] Recorded at 125 MHz. [c]Recorded at 500 MHz

Table S5. 1D and 2D NMR Data of sinudenoid J (**5**)

No.	5[a]			
	δ_C[b], type	δ_H[c] (J in Hz)	^1H-^1H COSY	HMBC
1	41.1, CH	2.10, m	H-2a, H-2b, H-14a, H-14b	
2a	47.0, CH_2	2.39, m	H-1	C-3
2b		2.27, m	H-1	
3	206.6, C			
4a	44.0, CH_2	3.12, s	H-5	
4b		2.87, m	H-5	C-3, C-6
5	82.6, CH	3.93, s	H-4a, H-4b	
6	198.2, C			
7	122.4, CH	6.48, s		C-6
8	158.3, C			
9a	36.6, CH_2	2.29, m	H-10	
9b		2.29, m	H-10	
10	80.6, CH	5.19, s	H-9a, H-9b, H-11	
11	148.5, CH	7.13, s	H-10	
12	132.6, C			
13a	21.4, CH_2	2.29, m	H-14a, H-14b	C-11, C-12, C-19
13b		2.16, m	H-14a, H-14b	C-11, C-12, C-19
14a	28.7, CH_2	1.72, m	H-13a, H-13b, H-1	
14b		1.72, m	H-13a, H-13b, H-1	
15	145.4, C			
16a	113.7, CH_2	4.62, s		
16b		4.79, s		
17	17.7, CH_3	1.60, s		C-1, C-15, C-16
18	28.7, CH_3	2.05, s		C-7, C-8, C-9
19	173.7, C			
20	58.5, CH_3	3.41 s		C-5

[a] In chloroform-*d*. [b] Recorded at 125 MHz. [c]Recorded at 500 MHz.

Table S6. 1D and 2D NMR Data of sinudenoid K (**6**)

No.	**6**[a]				
	δ_C[b], type	δ_H[c] (*J* in Hz)	^1H-^1H COSY	HMBC	NOEDY
1	149.6, C				
2	122.0, CH	5.50, s	H-3	C-1	H-16
3	87.1, CH	4.71, s	H-2		
4	74.8, C				
5a	38.3, CH_2	1.49, m	H-6a, H-6b		
5b		1.85, m	H-6a, H-6b		
6a	22.4, CH_2	2.42, m	H-5a, H-5b, H-7		
6b		1.90, m	H-5a, H-5b, H-7		
7	128.9, CH	5.27, d, (15.0)	H-6a, H-6b		
8	132.3, C				
9a	39.6, CH_2	2.12, m	H-10a, H-10b		
9b		2.12, m	H-10a, H-10b		
10a	25.0, CH_2	2.29, m	H-9a, H-9b, H-11		
10a		2.06, m	H-9a, H-9b, H-11		
11	133.3, CH	5.42, d, (11.5)	H-10		H-13
12	129.2, C				
13	81.2, CH	5.36, d, (6.0)	H-14	C-21	
14	84.0, CH	4.87, d, (6.0)	H-13	C-1, C-3	H-13
15	27.5, CH	2.60, m	H-16, H-17		
16	21.1, CH_3	1.16, d, (8.0)	H-15		
17	22.3, CH_3	1.03, d, (8.5)	H-15	C-1, C-15, C-17	
18	23.9, CH_3	0.99, s		C-3, C-4, C-5	H-3
19	15.8, CH_3	1.58, s		C-7, C-8, C-9	H-6a
20	12.9, CH_3	1.53, s		C-11, C-12, C-13	H-10a
21	169.9, C				
22	21.2, CH_3	2.06, s		C-21	

[a] In chloroform-*d*. [b] Recorded at 125 MHz. [c]Recorded at 500 MHz.

Table S7. 1D and 2D NMR Data of sinudenoid L (**7**)

No.	**7**[a]				
	δ_C[b], type	δ_H[c] (*J* in Hz)	^{1}H-^{1}H COSY	HMBC	NOEDY
1	40.3, CH	2.31, m	H-2a, H-2b, H-14a, H-14b		H-3
2a	33.1, CH_2	1.78, m	H-3, H-1		
2b		1.43, m	H-3, H-1		
3	63.1, CH	2.85, dd, (12.5, 3.5)	H-2a, H-2b		H-6
4	59.3, C				
5a	43.3, CH_2	1.45, m	H-6		H-3
5b		2.40, dd, (12.5, 3.5)	H-6		H-18
6	69.1, CH	5.66, m	H-5a, H-5b, H-7		
7	123.2, CH	5.23, d, (11.0)	H-6		H-9a
8	140.4, C				
9a	40.6, CH_2	1.98, m	H-10a, H-10b		
9b		2.27, m	H-10a, H-10b		
10a	24.7, CH_2	2.25, m	H-9a, H-9b, H-11		
10b		2.15, m	H-9a, H-9b, H-11		
11	124.0, CH	5.10, t, (9.0)	H-10a, H-10b		
12	133.9, C				
13a	34.9, CH_2	1.98, m	H-14a, H-14b		
13b		2.02, m	H-14a, H-14b		
14a	30.0, CH_2	1.64, m	H-13a, H-13b, H-1		
14b		1.55, m	H-13a, H-13b, H-1		
15	148.8, C				
16a	110.8, CH_2	4.71, s			
16b		4.69, s			
17	18.8, CH_3	1.66, s		C-1, C-15, C-16	
18	19.1, CH_3	1.33, s		C-3, C-4, C-5	
19	16.1, CH_3	1.72, s		C-7, C-8, C-9	
20	17.0, CH_3	1.58, s		C-11, C-12, C-13	H-10a
21	170.1, C				
22	21.5, CH_3	2.03, s		C-21	

[a] In chloroform-*d*. [b] Recorded at 125 MHz. [c]Recorded at 500 MHz.

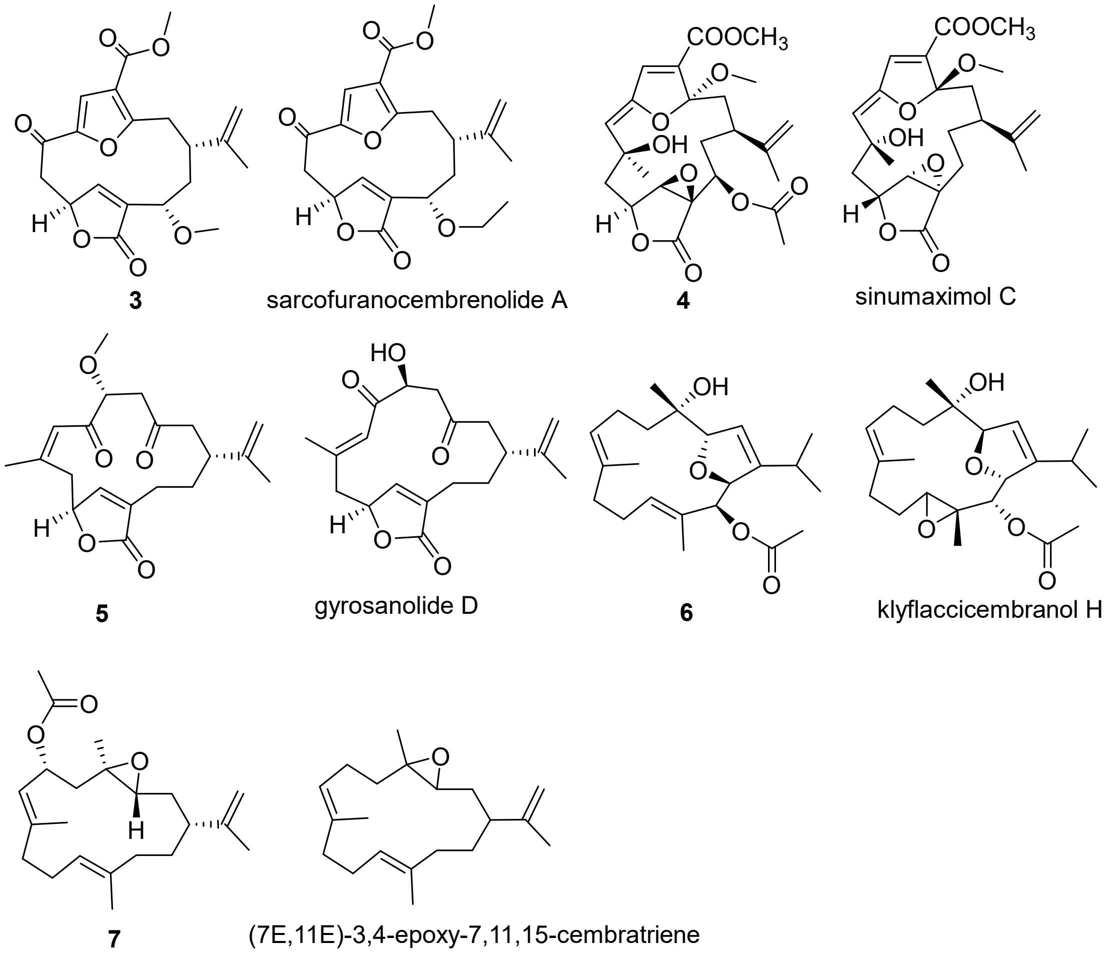

Figure S1. Structures of **3**–**7** and structurally related compounds of **3**–**7**

2. The Determination of the Relative and Absolute Configuration

The conformational search of all possible configurations was carried out by MacroModel integrated into Maestro V11.9 (Schrödinger Inc.). The OPLS3e force field and energy below a threshold of 10 kJ mol-1 were employed. Eliminating redundant conformers used a root-mean-squared-distance (RMSD) cutoff of 0.5 Å and the maximum iteration was 2500. After energy minimization, the unstable configurations were excluded. Excluding unstable conformers by conformational search, the remaining conformers were optimized with the density functional theory (DFT) at the B3LYP/6-31G (d, p) level, and all minima displayed no imaginary frequencies by vibrational frequency analysis at the same level. The populations of conformers were calculated according to the Boltzmann distribution theory and their relative Gibbs free energy. GIAO calculations of NMR shielding were accomplished for all stable conformations by the DFT GIAO model at PCM/mPW1PW91/6-31+G**evel for $DP4^+$ calculations. To determine the absolute configurations of **3**–**8**, the spin-allowed excitation energies and rotatory (Rn) and oscillator strengths (fn) of the lowest excited states of stable conformers were calculated for ECD spectra using TD-DFT method at the CAM-B3LYP/6-311G(d,p) level with IEFPCM solvent model for methanol in agreement with the experiment condition. All the calculations in this article were performed using the Gaussian 09.

Table S8. Experimental NMR data and calculated NMR data for **3**

Nuclei	SP^2	Experiment **3**	Calculation	
			Isomer 1 (1*R*, 9*R*,12*S*)	Isomer 2 (1*R*, 9*S*,12*S*)
C	X	164.1	168.639	169.513
C	X	119.2	119.925	120.619
C	X	118.0	122.093	121.7
C	X	149.8	151.28	150.445
C		32.2	33.567	34.412
C		40.5	43.877	43.593
C		38.2	37.054	41.005
C		71.4	78.707	73.028
C	X	135.5	134.598	137.31
C	X	152.3	158.601	159.179
C		76.9	78.303	80.809
C	X	172.6	171.293	175.191
C	X	184.5	186.939	188.457
C		45.9	41.456	46.787
C	X	144.8	153.249	151.643
C		19.1	18.67	19.487
C	X	114.0	110.802	112.76
C		57.0	57.113	56.443
C	X	162.9	165.587	165.542
C		52.0	53.519	53.389
C		5.33	5.256	5.177
C	X	7.52	7.666	7.71
H		2.70	2.585	2.627
H		3.62	3.922	3.725
H		2.43	2.659	2.547
H		2.07	1.356	1.993
H		1.16	1.875	1.177
H		4.08	3.797	3.951
H	X	6.76	7.517	6.961
H		3.38	3.428	3.293
H		3.32	3.065	3.172
H		1.83	1.982	1.92
H		1.83	1.982	1.92
H		1.83	1.982	1.92
H	X	4.95	5.016	5.106
H	X	4.98	5.026	5.136
H		3.19	3.101	3.082
H		3.19	3.101	3.082
H		3.19	3.101	3.082
H		3.88	3.854	3.859
H		3.88	3.854	3.859
H		3.88	3.854	3.854

Functional	Solvent?	Basis Set	Type of Data
mPW1PW91	PCM	6-31+G(d,p)	Unscaled Shifts

	Isomer 1	Isomer 2	Isomer 3	Isomer 4	Isomer 5	Isomer 6
sDP4+ (H data)	0.00%	100.00%	–	–	–	–
sDP4+ (C data)	0.00%	100.00%	–	–	–	–
sDP4+ (all data)	0.00%	100.00%	–	–	–	–
uDP4+ (H data)	0.00%	100.00%	–	–	–	–
uDP4+ (C data)	0.04%	99.96%	–	–	–	–
uDP4+ (all data)	0.00%	100.00%	–	–	–	–
DP4+ (H data)	0.00%	100.00%	–	–	–	–
DP4+ (C data)	0.00%	100.00%	–	–	–	–
DP4+ (all data)	0.00%	100.00%	–	–	–	–

Figure S2. The DP4+ results between calculated and experimental NMR data for **3**

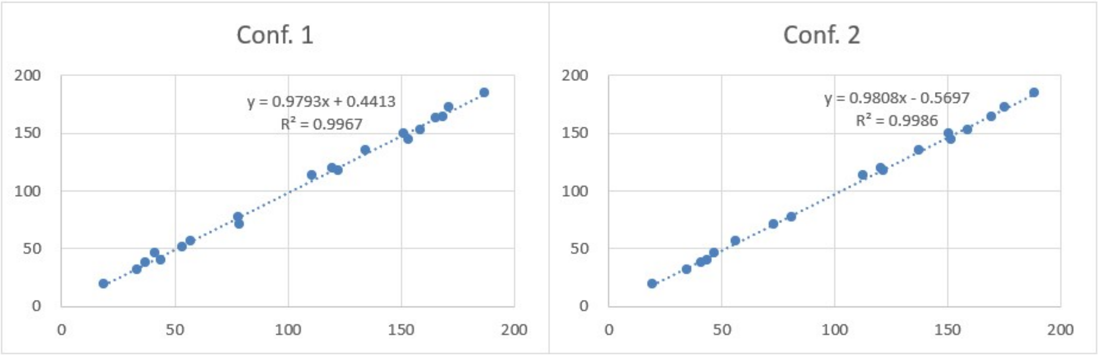

Figure S3. Correlation of experimental and calculated chemical shifts of compound **3**

Table S9. Experimental NMR data and calculated NMR data for **4**

Nuclei	SP[2]	Experiment **4**	Calculation			
			Isomer 1 (1*R*,3*S*,8*R*,*10R*,*11S*, *12S*,*13S*)	Isomer 2 (1*S*,3*S*,8*R*,10*R*,11*S*, 12*S*,13*S*)	Isomer 3 (1*S*,3*R*,8*R*,10*R*,11*S*, 12*S*,13*S*)	Isomer 2 (1*R*,3*R*,8*R*,10*R*,11*S*, 12*S*,13*S*)
C	X	130.7	134.352	134.94	136.107	134.603
C	X	139.5	142.512	144.195	139.957	141.763
C	X	150.2	151.51	150.661	152.427	154.305
C	X	116.7	119.435	119.557	118.485	120.352
C	X	117.9	119.515	120.621	120.777	116.344
C		71.5	75.238	75.61	75.442	75.075
C		42	42.551	42.406	44.751	40.603
C		73.8	80.17	79.921	77.441	78.352
C		62.3	62.669	63.745	63.986	62.688
C		58.4	63.047	62.998	61.778	60.319
C		71.1	77.801	76.01	66.784	69.049
C		43.1	40.595	43.659	40.882	42.037
C		36.3	37.855	42.277	38.85	42.719
C		37.3	40.673	36.68	35.143	35.397
C	X	147.3	149.998	144.301	151.09	153.327
C	X	112.7	116.304	116.112	111.287	114.969
C		18.4	20.04	25.938	23.991	18.293
C	X	168.1	172.848	172.847	173.918	174.198
C		50.3	51.315	51.608	51.476	51.085
C	X	170.7	172.907	173.103	173.677	173.943
C		20.6	18.45	19.074	19.821	20.447
C		29.2	32.254	32.046	25.719	28.815
C	X	162.2	165.127	165.336	164.778	164.985
C		52	53.121	53.022	53.51	53.279
H	X	7.14	6.935	7.069	7.009	7.024
H	X	5.18	5.007	5.037	5.212	5.077
H		2.81	2.346	2.302	2.304	2.268
H		1.98	1.973	1.904	1.892	2.229
H		4.8	4.45	4.382	4.367	4.331
H		3.88	3.982	3.945	4.901	4.676
H		4.84	4.147	4.084	4.886	5.01
H		2.64	2.626	2.39	2.116	2.028
H		1.84	1.874	1.784	1.745	2.635
H		2.47	3.024	2.826	2.919	1.519
H		1.62	1.734	2.306	2.261	1.423
H		2.64	2.359	2.405	2.121	1.86
H	X	4.69	4.962	5.815	4.491	4.551
H	X	4.66	4.853	5.311	5.056	4.884
H		1.59	1.908	1.852	1.926	1.747
H		1.59	1.908	1.852	1.926	1.747
H		1.59	1.908	1.852	1.926	1.747
H		3.09	3.095	3.209	3.197	3.1
H		3.09	3.095	3.209	3.197	3.1
H		3.09	3.095	3.209	3.197	3.1
H		2.01	2.159	2.165	2.101	2.128
H		2.01	2.159	2.165	2.101	2.128
H		2.01	2.159	2.165	2.101	2.128
H		1.54	1.353	1.352	1.752	1.823
H		1.54	1.353	1.352	1.752	1.823
H		1.54	1.353	1.352	1.752	1.823
H		3.73	3.77	3.76	3.761	3.656
H		3.73	3.77	3.76	3.761	3.656
H		3.73	3.77	3.76	3.761	3.656

	A	B	C	D	E	F	G	H
1	Functional		Solvent?		Basis Set		Type of Data	
2	mPW1PW91		PCM		6-31+G(d,p)		Unscaled Shifts	
3								
4			Isomer 1	Isomer 2	Isomer 3	Isomer 4	Isomer 5	Isomer 6
5	sDP4+ (H data)		95.55%	0.00%	4.44%	0.00%	–	–
6	sDP4+ (C data)		53.92%	0.00%	0.01%	46.07%	–	–
7	sDP4+ (all data)		100.00%	0.00%	0.00%	0.00%	–	–
8	uDP4+ (H data)		100.00%	0.00%	0.00%	0.00%	–	–
9	uDP4+ (C data)		85.48%	11.78%	2.74%	0.00%	–	–
10	uDP4+ (all data)		100.00%	0.00%	0.00%	0.00%	–	–
11	DP4+ (H data)		100.00%	0.00%	0.00%	0.00%	–	–
12	DP4+ (C data)		100.00%	0.00%	0.00%	0.00%	–	–
13	DP4+ (all data)		100.00%	0.00%	0.00%	0.00%	–	–

Figure S4. The DP4$^+$ results between calculated and experimental NMR data for **4**

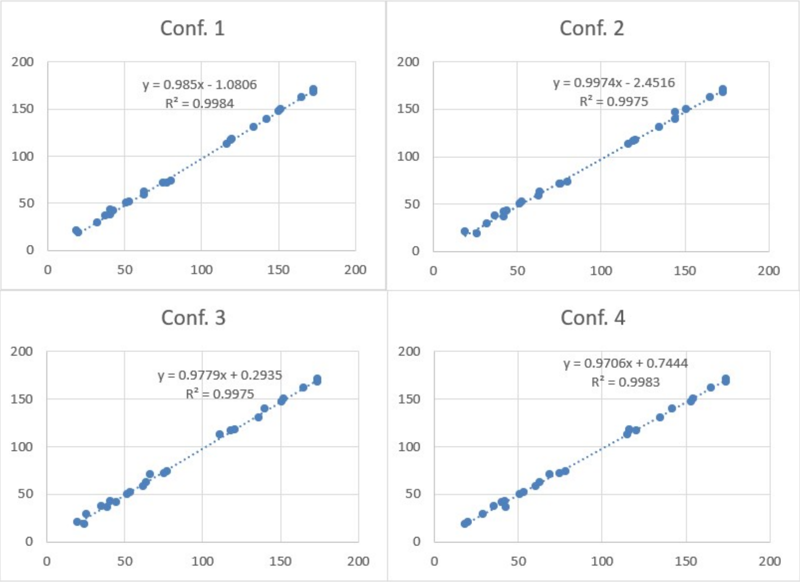

Figure S5. Correlation of experimental and calculated chemical shifts of compound **4**

Table S10. Experimental NMR data and calculated NMR data for **6**

Nuclei	SP^2	Experiment **7**	Calculation	
			Isomer 1（3*S*,4*S*,13*R*,*14R*)	Isomer 2 (3*S*,4*S*,13*S*,14*S*)
C	X	132.3	136.447	136.871
C		39.6	40.782	41.311
C		25.0	27.299	28.029
C	X	133.3	126.249	125.721
C		38.3	43.187	44.344
C		22.4	27.383	25.628
C	X	128.9	129.487	131.072
C		87.1	92.254	92.84
C	X	149.6	150.258	150.743
C		74.8	77.428	74.695
C	X	129.2	133.003	134.331
C		15.8	15.843	16.268
C		12.9	15.138	15.427
C		27.5	29.362	29.67
C		21.1	21.385	20.065
C		22.3	19.766	21.514
C		81.2	77.051	78.54
C		84.0	87.027	86.151
C	X	122.0	122.582	122.466
C	X	169.9	173.481	173.601
C		21.2	20.545	20.825
C		23.9	26.531	24.704
H		2.12	2.198	2.18
H		2.12	2.198	2.18
H		2.06	2.64	2.042
H		2.29	2.034	2.711
H	X	5.42	5.257	5.387
H		1.85	1.711	1.787
H		1.49	1.507	1.573
H		2.42	2.54	2.413
H		1.90	2.124	2.036
H	X	5.27	5.54	5.213
H		4.71	4.597	4.669
H		1.58	1.654	1.751
H		1.58	1.654	1.751
H		1.58	1.654	1.751
H		1.53	1.868	1.818
H		1.53	1.868	1.818
H		1.53	1.868	1.818
H		2.60	2.296	2.212
H		1.16	1.08	1.204
H		1.16	1.08	1.204
H		1.16	1.08	1.204
H		1.03	1.16	1.136
H		1.03	1.16	1.136
H		1.03	1.16	1.136
H		5.36	5.406	5.355
H		4.87	5.133	5.041
H	X	5.50	5.611	5.621
H		2.06	2.141	2.109
H		2.06	2.141	2.109
H		2.06	2.141	2.109
H		0.99	1.151	1.217
H		0.99	1.151	1.217

H	0.99	1.151	1.217

Functional	Solvent?	Basis Set	Type of Data
mPW1PW91	PCM	6-31+G(d,p)	Unscaled Shifts

	Isomer 1	Isomer 2	Isomer 3	Isomer 4	Isomer 5	Isomer 6
sDP4+ (H data)	0.01%	99.99%	–	–	–	–
sDP4+ (C data)	1.85%	98.15%	–	–	–	–
sDP4+ (all data)	0.00%	100.00%	–	–	–	–
uDP4+ (H data)	2.86%	97.14%	–	–	–	–
uDP4+ (C data)	72.76%	27.24%	–	–	–	–
uDP4+ (all data)	7.29%	92.71%	–	–	–	–
DP4+ (H data)	0.00%	100.00%	–	–	–	–
DP4+ (C data)	4.80%	95.20%	–	–	–	–
DP4+ (all data)	0.00%	100.00%	–	–	–	–

Figure S6. The DP4$^+$ results between calculated and experimental NMR data for **6**

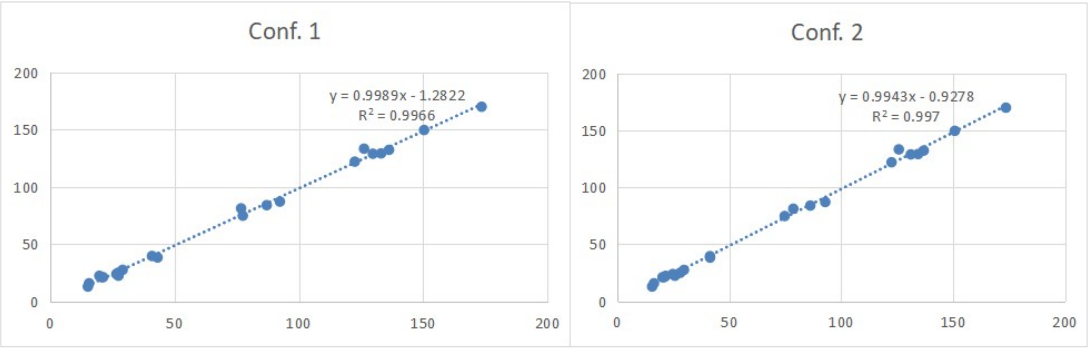

Figure S7. Correlation of experimental and calculated chemical shifts of **6**

Table S11. Experimental NMR data and calculated NMR data for **7**

Nuclei	SP^2	Experiment **7**	Calculation	
			Isomer 1（1*R*,3*R*,4*R*,6*R*)	Isomer 2（1*R*,3*R*,4*R*,6*S*)
C	X	140.4	147.444	149.291
C		40.6	42.207	43.968
C		24.7	27.603	28.426
C	X	124	125.445	127.575
C		43.3	45.165	44.831
C		69.1	76.478	73.011
C	X	123.2	127.419	124.579
C		63.1	65.341	67.688
C		34.9	37.113	39.666
C		30	30.634	32.947
C		40.3	45.187	45.122
C		33.1	36.702	31.484
C		59.3	63.978	61.776
C	X	133.9	138.799	138.761
C		16.1	20.219	17.996
C		17	20.499	16.442
C	X	148.8	154.378	155.437
C		18.8	21.155	20.172
C	X	170.1	174.705	173.656
C		19.1	22.37	18.548
C		21.5	22.767	22.324
C	X	110.8	110.823	111.229
H		2.85	3.074	2.784
H		2.27	2.561	2.317
H		1.98	2.54	2.594
H		2.25	2.729	2.435
H		2.15	2.58	2.601
H	X	5.1	5.438	5.548
H		2.4	2.408	1.54
H		1.45	2.555	2.45
H		5.66	4.927	5.585
H	X	5.23	5.662	5.422
H		2.02	2.273	2.219
H		1.98	2.217	2.286
H		1.64	1.88	1.58
H		1.55	1.769	1.802
H		2.31	2.61	2.426
H		1.43	1.699	1.748
H		1.78	2.052	1.672
H		1.72	2.109	2.168
H		1.72	2.109	2.168
H		1.72	2.109	2.168
H		1.58	2.03	1.934
H		1.58	2.03	1.934
H		1.58	2.03	1.934
H		1.66	2.067	2.024
H		1.66	2.067	2.024
H		1.66	2.067	2.024
H		1.33	1.639	1.434
H		1.33	1.639	1.434
H		1.33	1.639	1.434
H		2.03	2.448	2.603
H		2.03	2.448	2.603
H		2.03	2.448	2.603
H	X	4.71	5.097	5.115
H	X	4.69	5.198	5.175

Functional	Solvent?	Basis Set	Type of Data
mPW1PW91	PCM	6-31+G(d,p)	Unscaled Shifts

	Isomer 1	Isomer 2	Isomer 3	Isomer 4	Isomer 5	Isomer 6	Iso
sDP4+ (H data)	100.00%	0.00%	–	–	–	–	
sDP4+ (C data)	99.57%	0.43%	–	–	–	–	
sDP4+ (all data)	100.00%	0.00%	–	–	–	–	
uDP4+ (H data)	0.00%	100.00%	–	–	–	–	
uDP4+ (C data)	99.62%	0.38%	–	–	–	–	
uDP4+ (all data)	0.08%	99.92%	–	–	–	–	
DP4+ (H data)	99.47%	0.53%	–	–	–	–	
DP4+ (C data)	100.00%	0.00%	–	–	–	–	
DP4+ (all data)	100.00%	0.00%	–	–	–	–	

Figure S8. The DP4+ results between calculated and experimental NMR data for **7**

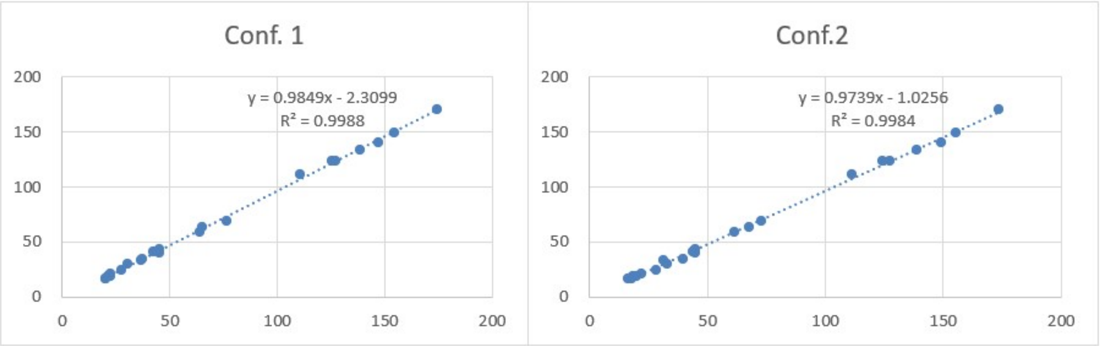

Figure S9. Correlation of experimental and calculated chemical shifts of **7**

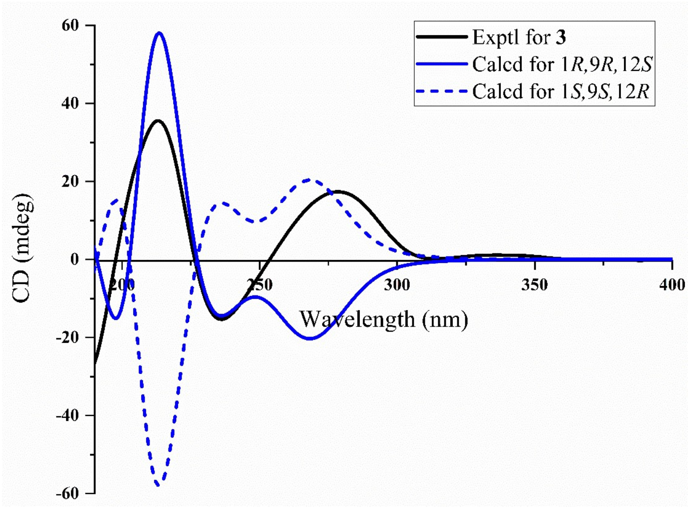

Figure S10. Experimental and calculated ECD spectra of **3a**

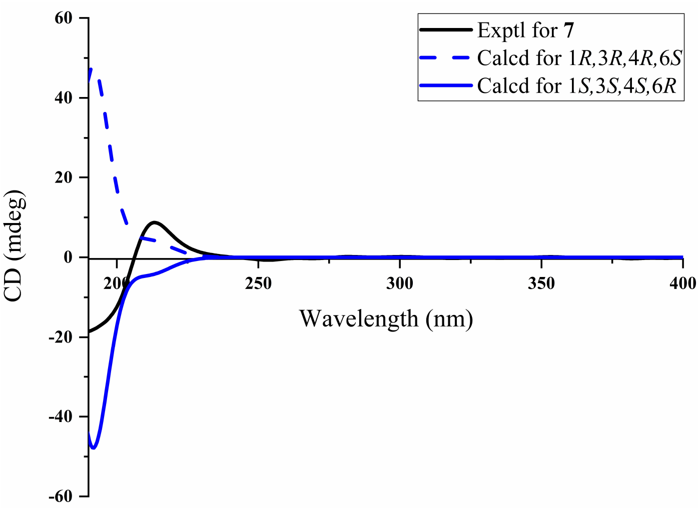

Figure S11. Experimental and calculated ECD spectra of **7b**

3. Biological assays

Table S12. Effects of samples on the anti-inflammatory effects of zebrafish.

Group	Concentration	Number of macrophages around nerve mound（mean ± SEM）	Inhibition rate
Control	—	2.33 ± 1.05	—
$CuSO_4$ model	40 μM	16.55 ± 2.15	—
Indomethacin	20 μM	10.20 ± 1.03	38.37%
**1**	20 μM	12.44 ± 0.60	24.83%
**2**	20 μM	15.10 ± 0.74	8.76%
**3**	20 μM	7.15 ± 0.71	56.80%
**4**	20 μM	13.88 ± 1.26	16.13%
**5**	20 μM	16.29 ± 0.87	1.57%
**6**	20 μM	13.80 ± 1.11	16.62%
**7**	20 μM	15.71 ± 1.17	5.08%

Table S13. Effects of samples on the anti-thrombotic effects of zebrafish.

Group	Concentration	Staining intensity of erythrocytes in heart（mean±SEM）	Area of caudal vein thrombosis（μm^2）（mean±SEM）
Control	—	457249 ±13225	8780 ±133
Arachidonic Acid model	80 μM	99571 ±13763	4130 ±154
Aspirin	22.5 μg/mL	342133 ±10896	1014 ±131
**5**	20 μM	203754 ±14796	2712 ±291
**7**	20 μM	192657 ±16477	2660 ±380

4. Computational Details

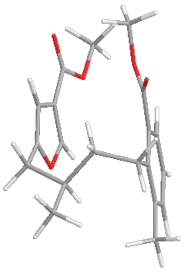

Conf.1
1a (73.73%)

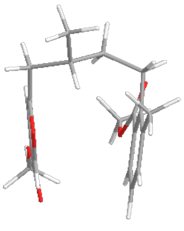

Conf.1
1b (19.11%)

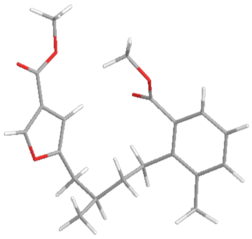

Conf.1
1c (1.21%)

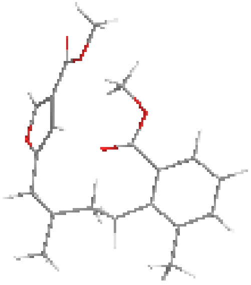

Conf.1
1d (1.09%)

Figure S12. Stable conformers of **1**

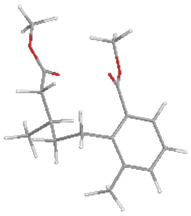

Conf.1
2a (26.77%)

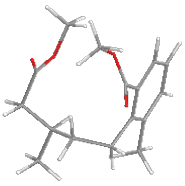

Conf.1
2b (16.58%)

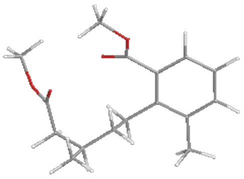

Conf.1
2c (8.31%)

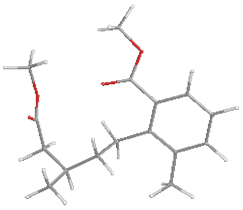

Conf.1
2d (7.55%)

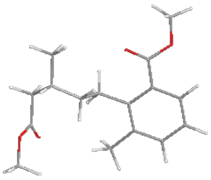

Conf.1
2e (5.44%)

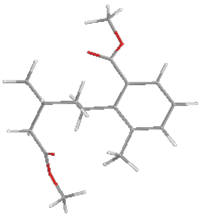

Conf.1
2f (5.42%)

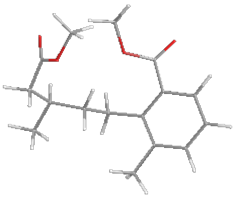

Conf.1
2g (5.35%)

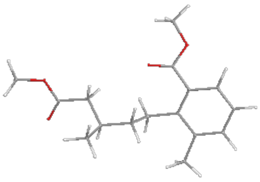

Conf.1
2h (4.62%)

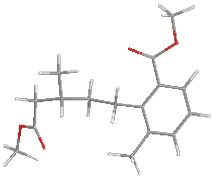

Conf.1
2i (4.50%)

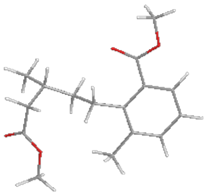

Conf.1
2j (3.14%)

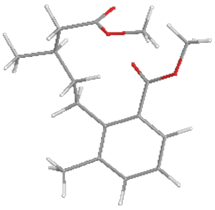

Conf.1
2k (2.83%)

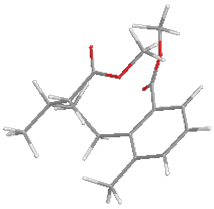

Conf.1
2l (2.66%)

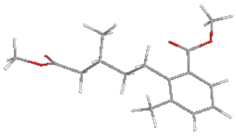

Conf.1
2m (2.50%)

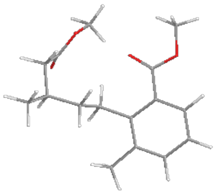

Conf.1
2n (2.34%)

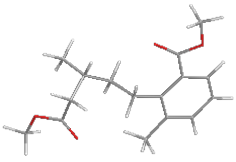

Conf.1
2o (2.00%)

Figure S13. Stable conformers of **2**

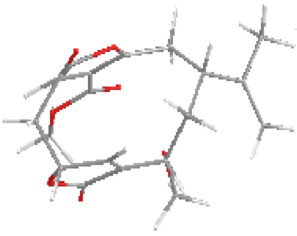

Conf.1
3a (66.78%)

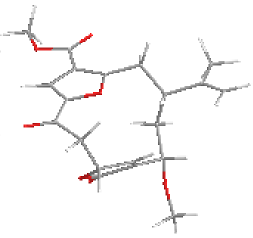

Conf.1
3b (25.79%)

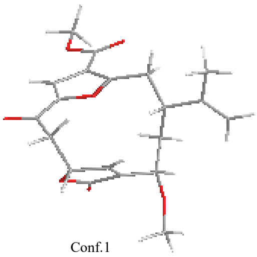

Conf.1
3c (2.71%)

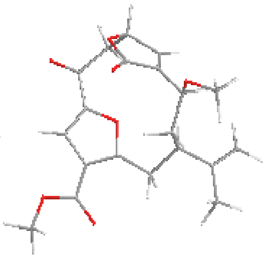

Conf.1
3d (1.71%)

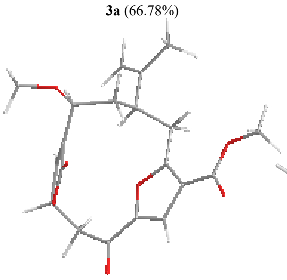

Conf.1
3e (1.21%)

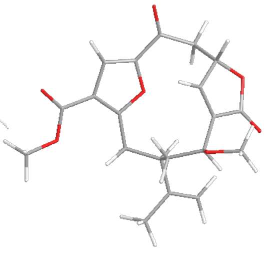

Conf.2
3a (42.14%)

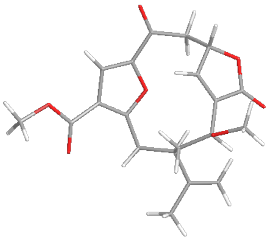

Conf.2
3b (32.78%)

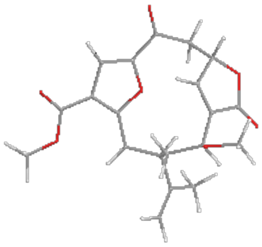

Conf.2
3c (10.95%)

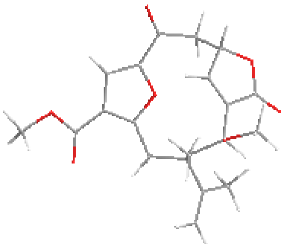

Conf.2
3d (7.89%)

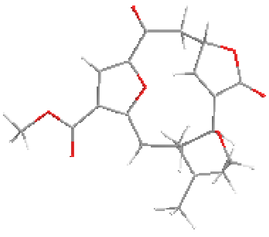

Conf.2
3e (6.24%)

Figure S14. Stable conformers of **3**

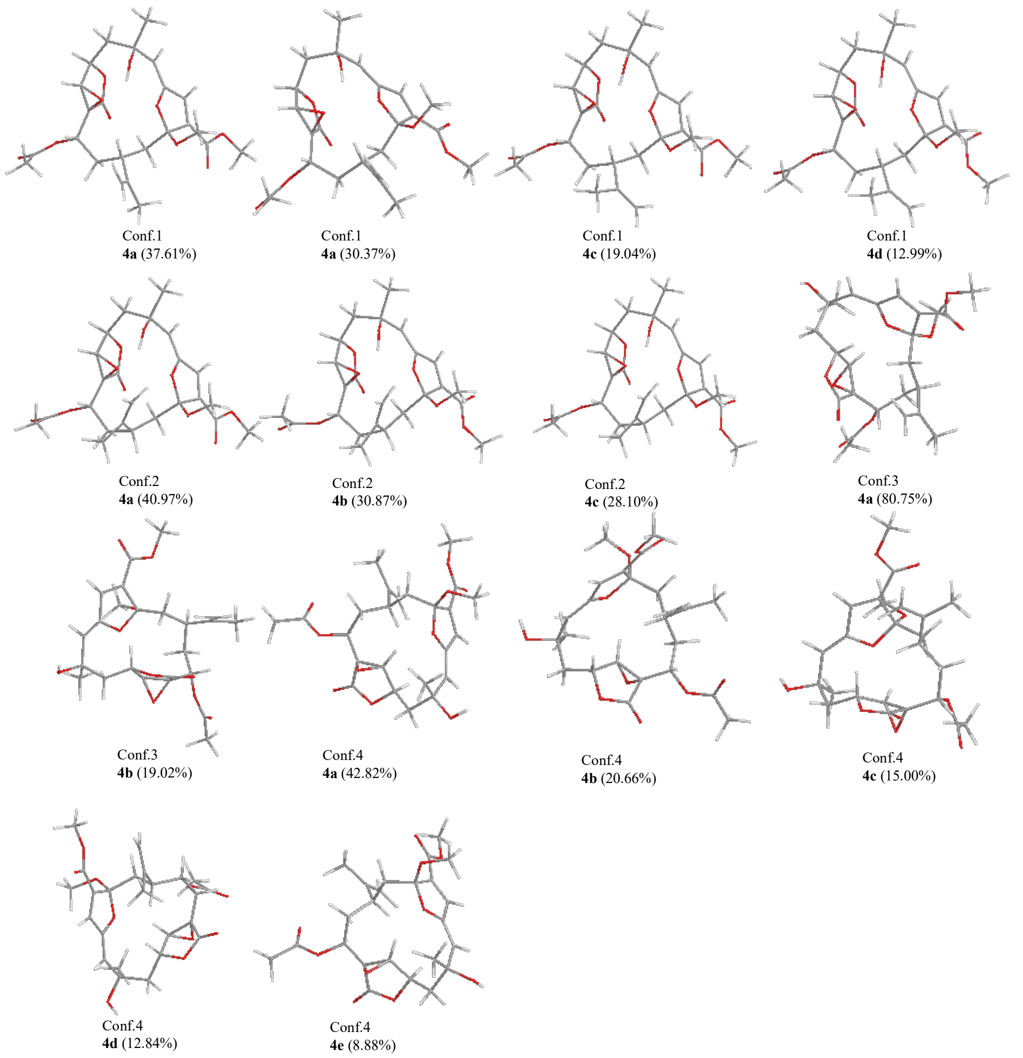

Figure S15. Stable conformers of **4**

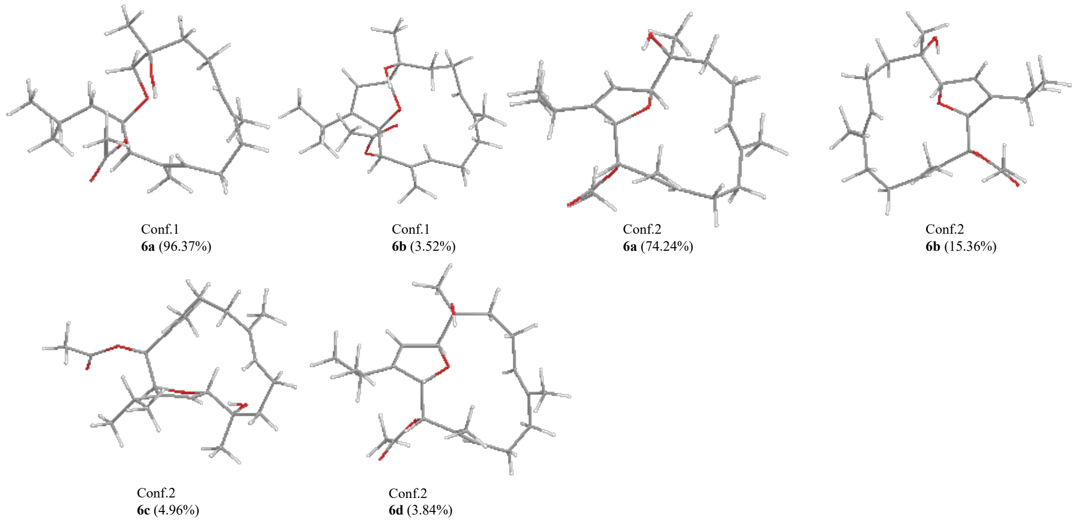

Figure S16. Stable conformers of **6**

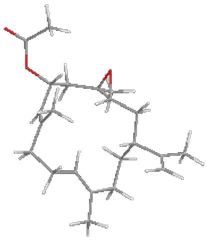

Conf.1
7a (23.55%)

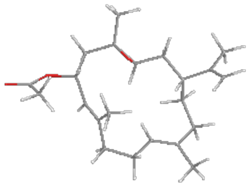

Conf.1
7b (11.32%)

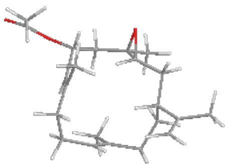

Conf.1
7c (10.46%)

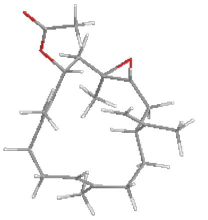

Conf.1
7d (9.31%)

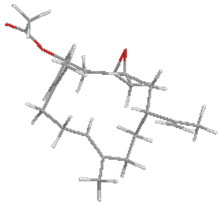

Conf.1
7e (7.75%)

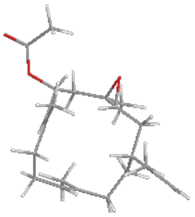

Conf.1
7f (7.39%)

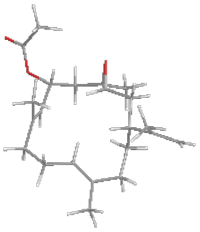

Conf.1
7g (6.02%)

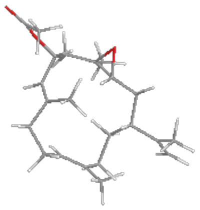

Conf.1
7h (5.70%)

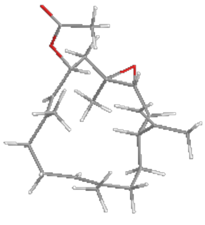

Conf.1
7i (4.03%)

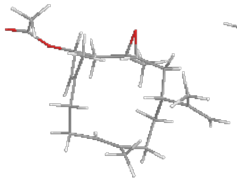

Conf.1
7j (3.36%)

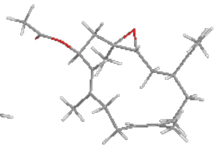

Conf.2
7a (33.03%)

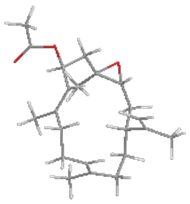

Conf.2
7b (21.30%)

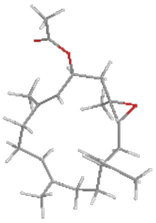

Conf.2
7c (20.39%)

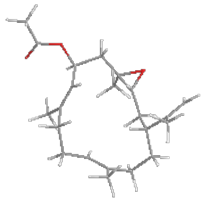

Conf.2
7d (8.19%)

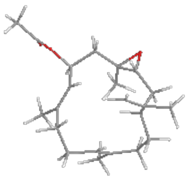

Conf.2
7e (7.48%)

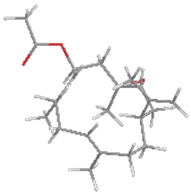

Conf.2
7f (5.42%)

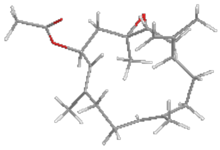

Conf.2
7g (3.51%)

Figure S17. Stable conformers of **7**

Table S14. Optimized Z-Matrixes of **1–4**, **6** and **7** in the Gas Phase (Å) at B3LYP/6-31G (d, p) level

	Con f. 1- 1a			Con f. 1- 1b		
C	-2.72993	1.54677	1.2312	-2.71237	1.47788	1.60076
C	-3.34928	1.51644	0.01249	-3.36524	1.59729	0.40527
C	-2.42824	0.88295	-0.89665	-2.47293	1.07223	-0.59869
C	-1.31707	0.57226	-0.17741	-1.3448	0.67494	0.04906
O	-1.50024	0.98823	1.13623	-1.49121	0.92872	1.40769
C	-0.05835	-0.17077	-0.44982	-0.0873	-0.01308	-0.34692
C	-0.02002	-1.52047	0.30727	-0.01861	-1.45189	0.21982
C	-1.09782	-2.4893	-0.2227	-1.09205	-2.35273	-0.42612
C	-1.77961	-3.33086	0.87711	-1.76513	-3.33628	0.55483
C	-2.58095	-2.48306	1.84722	-2.53663	-2.62716	1.6519
C	-2.09562	-2.27673	3.15902	-2.01811	-2.6152	2.96758
C	-2.79153	-1.43371	4.03416	-2.67762	-1.89333	3.96971
C	-3.9625	-0.78763	3.64772	-3.84376	-1.18011	3.70683
C	-4.45456	-0.98539	2.36543	-4.37089	-1.19147	2.42358
C	-3.78113	-1.82868	1.46561	-3.7361	-1.91153	1.39664
C	1.37658	-2.14252	0.2539	1.38527	-2.03929	0.06412
C	-4.39608	-2.02214	0.12197	-4.39385	-1.91548	0.05939
C	-0.84027	-2.95236	3.66392	-0.76388	-3.37337	3.34159
O	-5.2811	-1.05039	-0.18115	-5.34723	-0.96606	-0.04652
O	-4.17809	-2.9464	-0.64959	-4.15853	-2.68143	-0.86549
C	-5.9461	-1.18005	-1.4522	-6.06492	-0.93221	-1.29417
C	-4.70663	1.94995	-0.30221	-4.72887	2.10047	0.269
O	-5.3967	2.31084	0.79709	-5.11484	2.18372	-1.01762
C	-6.75285	2.7472	0.57708	-6.44419	2.69334	-1.24506
O	-5.18119	1.9743	-1.43106	-5.45132	2.41572	1.2076
H	-3.0174	1.91963	2.20158	-2.97742	1.73276	2.61506
H	-2.59806	0.67677	-1.94379	-2.66228	0.99683	-1.65968
H	0.02001	-0.33723	-1.53036	-0.03169	-0.03233	-1.44144
H	0.80346	0.43966	-0.14939	0.77465	0.56369	0.01385
H	-0.24498	-1.2864	1.35407	-0.23128	-1.36653	1.29154
H	-1.86895	-1.93115	-0.7601	-1.86969	-1.73558	-0.88272
H	-0.64593	-3.16986	-0.95638	-0.63971	-2.92893	-1.24416
H	-2.43203	-4.06546	0.40189	-2.4345	-3.98791	-0.00956
H	-1.01617	-3.88865	1.42327	-1.00025	-3.97498	1.001
H	-2.40273	-1.28595	5.03839	-2.26287	-1.89284	4.97446
H	-4.48653	-0.13593	4.34073	-4.33766	-0.62103	4.49611
H	-5.36462	-0.48943	2.05228	-5.27693	-0.6401	2.20683
H	1.39478	-3.10818	0.77361	1.42395	-3.06552	0.44907
H	2.12376	-1.49284	0.72575	2.1309	-1.4476	0.60904
H	1.6884	-2.31781	-0.78409	1.68343	-2.06894	-0.99209
H	-0.64439	-2.66031	4.69994	0.10936	-3.04448	2.7675
H	-0.92906	-4.0452	3.63767	-0.53724	-3.22949	4.4022
H	0.04226	-2.68786	3.07117	-0.87419	-4.45085	3.1691
H	-6.48073	-2.13154	-1.5069	-5.40105	-0.62186	-2.10494
H	-6.64394	-0.34588	-1.50059	-6.48809	-1.91423	-1.51837
H	-5.22049	-1.11556	-2.26691	-6.85984	-0.2019	-1.15102
H	-7.12885	3.02558	1.56166	-6.60298	2.61742	-2.32069
H	-6.7745	3.60737	-0.09676	-7.18294	2.0966	-0.70574
H	-7.35034	1.93263	0.15953	-6.50706	3.73651	-0.92355
	Con f. 1- 1c			Con f. 1- 1d		
C	-2.0742	1.94031	-2.21813	-3.02487	2.48472	0.27853
C	-3.10139	2.01444	-1.31752	-3.50519	1.86169	-0.84088
C	-2.57501	1.54335	-0.06094	-2.4321	1.03585	-1.33669
C	-1.27402	1.21272	-0.27968	-1.38243	1.20731	-0.48957
O	-0.96288	1.46264	-1.61129	-1.74572	2.10761	0.50358
C	-0.18814	0.65311	0.57068	-0.03979	0.58329	-0.35004
C	0.05878	-0.86992	0.40965	0.07753	-0.28234	0.92967
C	-1.14507	-1.72383	0.83386	-0.93324	-1.43895	0.906
C	-1.53289	-1.58162	2.32018	-1.23991	-2.01228	2.30355
C	-2.50391	-2.66564	2.7352	-2.45003	-2.91826	2.27472
C	-2.00457	-3.88079	3.25581	-2.2564	-4.31576	2.19177
C	-2.89147	-4.9159	3.57207	-3.36219	-5.17005	2.13911
C	-4.26384	-4.78909	3.36264	-4.66413	-4.67357	2.16122
C	-4.76384	-3.61141	2.82306	-4.86882	-3.30355	2.22736
C	-3.89534	-2.55059	2.5192	-3.77792	-2.41619	2.27869
C	0.49826	-1.23207	-1.0135	1.51452	-0.77413	1.11507
C	-4.50756	-1.30176	1.98301	-4.07568	-0.9538	2.32602
C	-0.52491	-4.09024	3.47529	-0.87048	-4.91754	2.15583
O	-5.5319	-1.56694	1.15287	-5.40179	-0.7049	2.35163
O	-4.16994	-0.1594	2.26231	-3.26005	-0.04111	2.33598
C	-6.23204	-0.42105	0.62159	-5.79099	0.68201	2.37047
C	-4.44784	2.48663	-1.63578	-4.86624	2.02069	-1.34739
O	-5.23436	2.53	-0.54453	-5.06735	1.3345	-2.48612
C	-6.60391	2.92268	-0.76274	-6.3953	1.4126	-3.04366
O	-4.83341	2.80351	-2.75427	-5.73855	2.6891	-0.80327
H	-1.98954	2.18856	-3.26562	-3.4548	3.1903	0.97328
H	-3.11256	1.44834	0.86999	-2.45838	0.38703	-2.20024
H	0.74526	1.184	0.34558	0.14288	-0.03216	-1.23882
H	-0.43605	0.8757	1.61317	0.73379	1.36239	-0.32988
H	0.88909	-1.10682	1.09019	-0.17839	0.36756	1.77761
H	-2.01502	-1.47808	0.20957	-1.87245	-1.0871	0.47159
H	-0.90649	-2.77545	0.62925	-0.56551	-2.2387	0.24784
H	-0.62805	-1.64851	2.93108	-0.37558	-2.56416	2.67884
H	-1.96538	-0.59857	2.49997	-1.41455	-1.18297	2.99026
H	-2.49417	-5.84009	3.98411	-3.19591	-6.24266	2.07829
H	-4.93532	-5.60504	3.61376	-5.51181	-5.35136	2.12049
H	-5.82802	-3.49677	2.64861	-5.87704	-2.90964	2.23488

H	0.81152	-2.28161	-1.06322	1.61429	-1.37332	2.02741
H	1.34111	-0.61222	-1.34238	2.21724	0.06482	1.18833
H	-0.31783	-1.09479	-1.7323	1.82468	-1.40025	0.26818
H	-0.12204	-3.39648	4.22373	-0.9286	-5.99809	1.99409
H	-0.32999	-5.10794	3.82628	-0.32899	-4.75167	3.09558
H	0.04961	-3.93663	2.55439	-0.25903	-4.49066	1.35278
H	-7.06119	-0.83333	0.04634	-5.32012	1.20253	3.20792
H	-5.56819	0.15686	-0.02227	-5.51958	1.16881	1.43066
H	-6.60445	0.20864	1.43314	-6.87481	0.67259	2.49004
H	-7.07072	2.90149	0.22182	-7.12829	1.01996	-2.3342
H	-6.6487	3.93043	-1.18347	-6.64149	2.44688	-3.29711
H	-7.09849	2.21726	-1.43543	-6.36732	0.7966	-3.9425
		Con f. 1- 2a			Con f. 1- 2b	
C	-0.66465	-2.24906	-3.17138	-0.5751	-2.09828	-3.61374
C	0.18681	-2.84191	-4.26728	-0.53299	-3.1832	-4.6618
C	0.12762	-2.05735	-5.58438	0.51698	-2.88293	-5.75471
C	1.25777	-2.47309	-6.54044	1.94196	-2.90512	-5.16772
C	2.68607	-2.04273	-6.1288	2.92934	-1.97114	-5.89719
C	2.86305	-0.53854	-6.10313	2.51259	-0.51391	-5.82891
C	3.09374	0.15705	-7.31214	2.06019	0.15576	-6.98703
C	3.15408	1.55547	-7.30697	1.61262	1.47985	-6.89131
C	2.97681	2.28983	-6.13568	1.58584	2.15641	-5.67403
C	2.75267	1.61939	-4.94055	2.01323	1.50372	-4.5248
C	2.71575	0.21624	-4.91744	2.48242	0.18401	-4.59923
C	-1.23644	-2.24017	-6.26079	0.37096	-3.86006	-6.92344
C	2.57723	-0.42647	-3.57853	2.95129	-0.44784	-3.33343
C	3.30035	-0.57528	-8.61712	2.03291	-0.52132	-8.33781
O	1.67202	0.20483	-2.81136	2.19041	-0.08274	-2.28519
O	3.21818	-1.3892	-3.18014	3.90451	-1.20363	-3.21541
C	1.54568	-0.2703	-1.45414	2.53201	-0.67078	-1.01407
O	-1.16489	-1.13571	-3.17463	-0.23939	-2.22391	-2.44546
O	-0.78865	-3.09754	-2.13412	-1.018	-0.93794	-4.12653
C	-1.56119	-2.62091	-1.01195	-1.10991	0.17921	-3.21621
H	1.20762	-2.88226	-3.86905	-0.30988	-4.12937	-4.1597
H	-0.11856	-3.88457	-4.41803	-1.52737	-3.25728	-5.11758
H	0.2458	-0.99356	-5.346	0.30879	-1.87177	-6.12286
H	1.24899	-3.56589	-6.6555	1.91357	-2.63482	-4.10766
H	1.03302	-2.05778	-7.52953	2.33252	-3.93082	-5.20172
H	2.93928	-2.47008	-5.15885	3.91948	-2.09445	-5.45176
H	3.3876	-2.47652	-6.84862	3.01148	-2.28195	-6.94023
H	3.34119	2.07496	-8.2434	1.27299	1.98378	-7.79259
H	3.02084	3.37489	-6.15548	1.2333	3.1824	-5.62298
H	2.62689	2.17167	-4.01552	2.00255	2.01338	-3.5678
H	-1.31123	-1.62204	-7.16316	1.14404	-3.68334	-7.68012
H	-2.05586	-1.95688	-5.59118	-0.60607	-3.75741	-7.41043
H	-1.38834	-3.28662	-6.55645	0.47129	-4.89786	-6.58033
H	4.15019	-1.26669	-8.56053	3.03039	-0.85327	-8.64968
H	2.42549	-1.1686	-8.90481	1.3846	-1.40499	-8.33771
H	3.50097	0.13448	-9.42506	1.65784	0.16735	-9.10069
H	0.72761	0.30606	-1.02383	3.54157	-0.37689	-0.71634
H	1.31579	-1.33715	-1.43835	1.79719	-0.27996	-0.3105
H	2.4735	-0.08581	-0.906	2.46249	-1.75951	-1.07267
H	-1.09321	-1.73618	-0.57363	-1.87707	-0.01731	-2.46194
H	-2.58056	-2.3836	-1.32672	-0.14919	0.35907	-2.73381
H	-1.56618	-3.44189	-0.29466	-1.39489	1.03093	-3.83388
		Con f. 1- 2c			Con f. 1- 2d	
C	-0.63059	-2.71409	-3.46906	-0.70499	-2.98739	-3.58629
C	-0.90595	-3.04073	-4.91844	-1.11815	-2.95942	-5.03871
C	0.32097	-3.38459	-5.78556	0.01301	-3.24787	-6.05232
C	1.41656	-2.30696	-5.74333	1.23738	-2.33719	-5.86304
C	0.93065	-0.89238	-6.1145	0.90666	-0.83211	-5.89499
C	2.07372	0.0988	-6.08979	2.14699	0.03376	-5.92376
C	2.71609	0.48437	-7.28545	2.7433	0.34447	-7.16719
C	3.81692	1.34821	-7.2299	3.89286	1.14085	-7.20468
C	4.31571	1.81803	-6.01604	4.46248	1.65497	-6.04051
C	3.71249	1.41692	-4.82993	3.87448	1.37512	-4.81485
C	2.59248	0.57418	-4.86539	2.73173	0.56079	-4.74949
C	0.90156	-4.76595	-5.45752	0.43019	-4.72294	-6.06777
C	1.96553	0.20673	-3.56246	2.17662	0.26151	-3.40118
C	2.24533	-0.02294	-8.62791	2.15326	-0.1483	-8.46737
O	2.8916	-0.10069	-2.6384	2.36029	1.28144	-2.54126
O	0.76586	0.1974	-3.32547	1.62723	-0.77907	-3.06759
C	2.3966	-0.43634	-1.32368	1.90941	1.05785	-1.18977
O	0.31557	-3.10695	-2.8032	0.00085	-3.83264	-3.05808
O	-1.6086	-1.94515	-2.95931	-1.24344	-1.96279	-2.89954
C	-1.51461	-1.6253	-1.55515	-0.96858	-1.93061	-1.48266
H	-1.59689	-3.89579	-4.9129	-1.90124	-3.72146	-5.15315
H	-1.46758	-2.20584	-5.34647	-1.58602	-1.99429	-5.24557
H	-0.06063	-3.42374	-6.81557	-0.42131	-3.01666	-7.03507
H	1.86857	-2.28534	-4.74428	1.7324	-2.57241	-4.91465
H	2.21715	-2.60003	-6.43491	1.96	-2.56782	-6.65533
H	0.47371	-0.91843	-7.10708	0.30323	-0.6219	-6.78484
H	0.15547	-0.57217	-5.41723	0.29226	-0.57697	-5.0303
H	4.29479	1.64862	-8.15896	4.34442	1.36508	-8.1679
H	5.17307	2.4846	-5.99633	5.35565	2.27083	-6.09195
H	4.09196	1.76347	-3.87436	4.3024	1.7678	-3.89944
H	1.70478	-5.0218	-6.15893	-0.43582	-5.37612	-6.23083
H	0.13121	-5.54339	-5.53086	0.89995	-5.01574	-5.12404
H	1.31553	-4.7958	-4.44501	1.14849	-4.90837	-6.87542
H	2.89506	0.34612	-9.4271	1.16158	0.28534	-8.64892
H	2.24905	-1.1185	-8.67188	2.79559	0.13032	-9.30793
H	1.22233	0.30388	-8.85161	2.03322	-1.23708	-8.4829
H	3.28664	-0.60918	-0.71836	2.17232	1.96494	-0.64523
H	1.81155	0.39061	-0.91381	0.82749	0.90353	-1.17202
H	1.78705	-1.3409	-1.37483	2.4154	0.19157	-0.75607
H	-2.4427	-1.10552	-1.31562	-1.38859	-2.81569	-0.99731
H	-1.42396	-2.53742	-0.96013	0.10655	-1.88195	-1.30481

H	-0.65814	-0.97313	-1.3759	-1.45876	-1.02907	-1.11459
	Con f. 1- 2e			Con f. 1- 2f		
C	-1.55713	-3.0169	-5.24032	-1.41797	-3.23823	-5.16848
C	-1.33189	-2.65694	-3.79489	-0.9689	-3.13451	-3.73303
C	0.13521	-2.8389	-3.34139	0.5527	-3.3231	-3.56873
C	1.13942	-2.12461	-4.26701	1.37144	-2.35457	-4.44407
C	0.91199	-0.60778	-4.43131	1.04997	-0.86123	-4.23419
C	2.01047	0.04129	-5.24461	1.9074	0.0273	-5.10703
C	1.89509	0.0906	-6.65278	1.47779	0.3205	-6.42195
C	2.91611	0.6736	-7.41122	2.26433	1.13734	-7.24095
C	4.04655	1.22643	-6.81138	3.46475	1.68386	-6.78872
C	4.16171	1.20438	-5.42865	3.89028	1.41516	-5.49554
C	3.15988	0.60759	-4.64466	3.12721	0.58426	-4.65741
C	0.27584	-2.41376	-1.87594	0.92819	-3.23102	-2.08601
C	3.36582	0.5817	-3.16996	3.65993	0.30124	-3.29575
C	0.6828	-0.46199	-7.36424	0.17842	-0.22601	-6.96618
O	4.09808	1.63049	-2.74548	4.42844	1.30854	-2.83599
O	2.9541	-0.27308	-2.39764	3.46543	-0.71344	-2.64021
C	4.40934	1.65341	-1.33812	5.05228	1.09101	-1.55485
O	-1.96552	-2.25494	-6.10362	-1.06041	-4.10659	-5.94948
O	-1.24366	-4.29974	-5.49172	-2.27869	-2.26287	-5.50735
C	-1.39818	-4.73791	-6.85774	-2.79496	-2.3097	-6.85494
H	-1.65922	-1.62326	-3.65834	-1.30184	-2.18193	-3.31341
H	-1.97517	-3.29772	-3.17959	-1.48894	-3.9287	-3.18099
H	0.36115	-3.91098	-3.4032	0.79009	-4.33672	-3.91684
H	2.14727	-2.28928	-3.87209	2.43496	-2.51842	-4.24142
H	1.11509	-2.59896	-5.25649	1.21484	-2.60795	-5.49871
H	-0.04701	-0.43878	-4.9284	-0.0004	-0.67966	-4.477
H	0.84722	-0.13971	-3.44837	1.18331	-0.60123	-3.18355
H	2.81738	0.69626	-8.49356	1.92462	1.34958	-8.25159
H	4.828	1.67278	-7.41945	4.06116	2.31452	-7.44159
H	5.03292	1.63176	-4.94582	4.82195	1.83066	-5.12947
H	1.30218	-2.56563	-1.52379	2.00371	-3.38906	-1.94668
H	-0.3932	-3.00091	-1.23537	0.39509	-3.98912	-1.4995
H	0.02601	-1.35624	-1.73401	0.67711	-2.25061	-1.66479
H	0.80464	-0.37592	-8.44823	-0.68681	0.13683	-6.39826
H	0.50986	-1.51646	-7.12824	0.04367	0.07829	-8.00829
H	-0.23097	0.07825	-7.08816	0.14818	-1.31994	-6.92967
H	4.97835	0.763	-1.05822	5.63946	1.9895	-1.36417
H	5.00974	2.5513	-1.19064	4.29325	0.95983	-0.77939
H	3.49177	1.70673	-0.74663	5.70042	0.21148	-1.59027
H	-1.10999	-5.78926	-6.85905	-3.43593	-1.43319	-6.94964
H	-2.43716	-4.62682	-7.17794	-1.97906	-2.26618	-7.57939
H	-0.74341	-4.16288	-7.51756	-3.37496	-3.22413	-7.00467
	Con f. 1- 2g			Con f. 1- 2h		
C	-0.66522	-3.34619	-3.22051	-1.61025	-3.27511	-3.50192
C	-0.96499	-3.65447	-4.66404	-0.28751	-2.62086	-3.81339
C	-0.78468	-2.38424	-5.53004	0.21217	-2.91704	-5.23674
C	0.68643	-1.93656	-5.53773	1.44742	-2.0697	-5.58012
C	0.88226	-0.49474	-6.04181	1.15455	-0.5639	-5.72468
C	2.31778	-0.03586	-5.90631	2.40492	0.23792	-6.00946
C	3.22082	-0.28433	-6.96612	2.82147	0.42034	-7.34757
C	4.55242	0.12983	-6.85636	3.98381	1.15064	-7.61762
C	5.01497	0.80455	-5.72724	4.74193	1.71923	-6.59503
C	4.13409	1.07031	-4.6892	4.33351	1.56312	-5.27791
C	2.79844	0.64106	-4.7614	3.17953	0.81944	-4.97825
C	-1.32246	-2.63045	-6.94174	0.52441	-4.4095	-5.39758
C	1.97571	0.99732	-3.56765	2.81635	0.65883	-3.54272
C	2.77358	-0.97258	-8.23458	2.02717	-0.14182	-8.50301
O	1.01345	0.11307	-3.26549	3.2382	1.69914	-2.79698
O	2.18412	1.99216	-2.88472	2.22018	-0.28878	-3.04928
C	0.19065	0.43442	-2.12302	2.98956	1.59962	-1.38058
O	-1.37146	-2.64793	-2.50747	-2.49961	-3.49041	-4.31112
O	0.49505	-3.87796	-2.80373	-1.72626	-3.57355	-2.19623
C	0.91045	-3.53196	-1.46427	-2.9845	-4.14875	-1.78447
H	-0.31561	-4.45856	-5.02279	-0.44431	-1.54406	-3.66881
H	-2.00562	-3.99132	-4.72183	0.45145	-2.92976	-3.06675
H	-1.38242	-1.59082	-5.06048	-0.59386	-2.65475	-5.93523
H	1.09931	-1.99568	-4.52716	2.21439	-2.21679	-4.81087
H	1.27164	-2.63362	-6.15217	1.87427	-2.43965	-6.52054
H	0.58022	-0.42386	-7.09029	0.43858	-0.42297	-6.54092
H	0.21907	0.16562	-5.47857	0.67636	-0.19119	-4.81818
H	5.23485	-0.0747	-7.67744	4.2954	1.27697	-8.65138
H	6.05161	1.1222	-5.66275	5.6423	2.28092	-6.82634
H	4.46662	1.6027	-3.80452	4.91081	1.99971	-4.47113
H	-1.23551	-1.7327	-7.56343	0.83531	-4.63479	-6.42437
H	-2.38027	-2.91786	-6.91735	-0.3492	-5.03071	-5.16896
H	-0.76459	-3.43638	-7.43576	1.33833	-4.71132	-4.72565
H	2.29865	-1.93943	-8.03609	1.03681	0.32526	-8.57642
H	2.0452	-0.36564	-8.78739	2.54883	0.03718	-9.44791
H	3.6274	-1.14778	-8.89587	1.865	-1.22106	-8.40841
H	-0.30587	1.39606	-2.27431	1.91541	1.54367	-1.18617
H	-0.53898	-0.37099	-2.06256	3.48634	0.71824	-0.96673
H	0.80052	0.47158	-1.21692	3.40718	2.50992	-0.95012
H	1.11067	-2.45909	-1.40129	-3.80632	-3.45972	-1.99584
H	0.14218	-3.81273	-0.7402	-3.15521	-5.09733	-2.30004
H	1.82502	-4.09885	-1.28998	-2.89121	-4.31134	-0.71058
	Con f. 1- 2i			Con f. 1- 2j		
C	-1.89147	-3.03682	-4.66758	-1.17097	-3.77532	-4.71084
C	-0.86667	-3.44688	-3.63693	-0.50558	-3.43519	-3.4023
C	0.57732	-3.31444	-4.13986	-0.51618	-1.91238	-3.10077
C	0.92482	-1.83888	-4.39606	0.18312	-1.0783	-4.18555
C	2.20255	-1.65053	-5.23411	1.65676	-1.46597	-4.42018
C	2.45153	-0.19509	-5.55667	2.38727	-0.45238	-5.27269
C	1.84466	0.36564	-6.70409	2.37551	-0.59228	-6.67926
C	2.04629	1.71737	-7.00192	3.036	0.34993	-7.4749
C	2.84585	2.52972	-6.19849	3.72191	1.42606	-6.91362

C	3.46119	1.98651	-5.07961	3.75595	1.56533	-5.53336
C	3.26153	0.63543	-4.74779	3.08678	0.64228	-4.7117
C	1.54356	-3.96624	-3.14557	-1.93146	-1.38555	-2.84289
C	3.91889	0.12224	-3.51357	3.12824	0.87244	-3.24104
C	0.99618	-0.47001	-7.63459	1.66699	-1.74648	-7.3482
O	5.04931	0.79817	-3.22684	4.2425	1.53095	-2.86455
O	3.51491	-0.78916	-2.80405	2.27312	0.5375	-2.43237
C	5.71904	0.41653	-2.00886	4.33633	1.861	-1.46446
O	-1.73514	-3.08499	-5.87845	-2.37683	-3.7689	-4.9079
O	-3.0377	-2.62928	-4.0976	-0.28207	-4.07435	-5.67557
C	-4.10259	-2.26217	-5.00071	-0.83227	-4.37302	-6.97577
H	-1.02531	-2.86143	-2.7246	-1.05505	-3.95602	-2.61109
H	-1.08576	-4.49297	-3.37905	0.52016	-3.81032	-3.41098
H	0.64185	-3.85213	-5.09522	0.06243	-1.79693	-2.17426
H	0.09285	-1.35062	-4.91584	-0.37692	-1.14939	-5.12765
H	1.04085	-1.31951	-3.43746	0.14165	-0.02764	-3.88012
H	3.05757	-2.07414	-4.70631	2.14961	-1.56898	-3.45109
H	2.09223	-2.21233	-6.16744	1.70331	-2.44271	-4.90712
H	1.56837	2.13778	-7.88322	3.01207	0.23309	-8.55543
H	2.98728	3.57765	-6.44652	4.22596	2.14799	-7.54951
H	4.08516	2.60486	-4.44479	4.28477	2.39607	-5.08077
H	2.57937	-3.90877	-3.49603	-1.89432	-0.33646	-2.52661
H	1.30079	-5.02513	-2.99537	-2.4321	-1.9595	-2.05398
H	1.49388	-3.46739	-2.16917	-2.5517	-1.44232	-3.74386
H	1.59585	-1.23514	-8.14441	2.10432	-2.71073	-7.06221
H	0.19022	-0.99608	-7.11124	0.6066	-1.78466	-7.08007
H	0.54136	0.1602	-8.40466	1.73564	-1.65892	-8.43662
H	6.04041	-0.62682	-2.0626	5.27669	2.40229	-1.35872
H	6.58307	1.07734	-1.93628	3.49612	2.49203	-1.16338
H	5.05822	0.55591	-1.14933	4.35045	0.9509	-0.85925
H	-3.79179	-1.42702	-5.63368	0.02598	-4.57423	-7.61631
H	-4.38412	-3.11466	-5.62428	-1.47912	-5.25225	-6.91861
H	-4.93503	-1.96649	-4.36204	-1.40067	-3.5202	-7.35424
		Con f. 1- 2k			Con f. 1- 2l	
C	-0.49036	-3.04687	-3.15943	1.00683	-2.76876	-3.78292
C	-0.95242	-3.5471	-4.50402	0.62442	-3.12366	-5.19803
C	-0.75619	-2.44137	-5.56768	-0.67698	-2.46763	-5.68707
C	0.72009	-2.0264	-5.64996	-0.69286	-0.92899	-5.59197
C	0.95816	-0.7365	-6.46167	0.35907	-0.14025	-6.40712
C	2.285	-0.09955	-6.10694	1.78153	-0.17949	-5.87574
C	3.37175	-0.15386	-7.00421	2.82405	-0.74742	-6.64214
C	4.60479	0.3964	-6.63067	4.11693	-0.81833	-6.10834
C	4.79331	0.98327	-5.38137	4.4124	-0.33417	-4.83601
C	3.73756	1.01822	-4.4787	3.40079	0.24045	-4.07829
C	2.48832	0.48876	-4.83537	2.09972	0.32687	-4.59469
C	-1.31172	-2.89445	-6.91878	-1.01413	-2.96963	-7.09502
C	1.3878	0.5906	-3.83177	1.07881	1.0014	-3.74489
C	3.24197	-0.80173	-8.36322	2.58796	-1.29562	-8.03015
O	1.85058	0.48496	-2.57028	1.21328	0.66932	-2.44773
O	0.20342	0.77304	-4.07395	0.22405	1.78272	-4.13578
C	0.87804	0.6874	-1.52168	0.2895	1.29398	-1.53303
O	-1.02236	-2.13246	-2.54738	0.23081	-2.44471	-2.89686
O	0.61531	-3.67487	-2.7276	2.32975	-2.89267	-3.58394
C	1.21263	-3.16456	-1.51558	2.80048	-2.63023	-2.24539
H	-0.40106	-4.45118	-4.7786	1.4677	-2.90383	-5.85521
H	-2.01646	-3.79626	-4.42659	0.50153	-4.21643	-5.21419
H	-1.32689	-1.5673	-5.22629	-1.47453	-2.81137	-5.01469
H	1.10554	-1.87257	-4.63658	-1.68089	-0.59484	-5.93375
H	1.31575	-2.84711	-6.07314	-0.63344	-0.64803	-4.53756
H	0.91822	-0.95525	-7.52945	0.34477	-0.48921	-7.44057
H	0.15066	-0.03234	-6.25042	0.03078	0.90324	-6.43291
H	5.43182	0.35543	-7.33501	4.90615	-1.26405	-6.70834
H	5.75778	1.40402	-5.1122	5.42285	-0.4	-4.44333
H	3.86776	1.46679	-3.50022	3.61022	0.63598	-3.09064
H	-1.23145	-2.09784	-7.66677	-1.9663	-2.54864	-7.43925
H	-2.36949	-3.17201	-6.83892	-1.10373	-4.06253	-7.11078
H	-0.76115	-3.76617	-7.29528	-0.24372	-2.6918	-7.82307
H	2.92305	-1.84785	-8.28639	2.17469	-0.53698	-8.70515
H	2.5068	-0.28621	-8.99317	3.52774	-1.65046	-8.4635
H	4.20127	-0.78296	-8.88893	1.88669	-2.13789	-8.02446
H	1.43446	0.57701	-0.59084	0.55657	0.91061	-0.54842
H	0.45078	1.69108	-1.59356	0.39831	2.38095	-1.56399
H	0.0875	-0.06181	-1.58746	-0.73653	1.01521	-1.78608
H	2.01519	-3.86136	-1.27313	3.88632	-2.70555	-2.30033
H	1.61977	-2.16654	-1.69657	2.50201	-1.63042	-1.92651
H	0.47829	-3.1309	-0.70781	2.4011	-3.37655	-1.55306
		Con f. 1- 2m			Con f. 1- 2n	
C	-2.14149	-3.67698	-4.11003	-0.97509	-2.7201	-3.76804
C	-1.40082	-2.37985	-4.32421	-0.11883	-3.84999	-4.27925
C	0.10956	-2.56677	-4.5322	0.41957	-3.62792	-5.70804
C	0.72878	-1.23564	-4.98516	1.49252	-2.52506	-5.77712
C	2.21246	-1.34534	-5.38518	0.96421	-1.08298	-5.63465
C	2.77347	-0.02104	-5.85314	2.06141	-0.05296	-5.79017
C	2.65693	0.33619	-7.21592	2.42351	0.39333	-7.08129
C	3.156	1.56698	-7.65514	3.44385	1.34007	-7.22469
C	3.78213	2.45367	-6.78029	4.10491	1.8742	-6.11933
C	3.92134	2.10628	-5.44412	3.73759	1.46649	-4.84431
C	3.41439	0.88295	-4.9733	2.73181	0.50085	-4.67567
C	0.76674	-3.11397	-3.25872	0.98898	-4.94795	-6.24036
C	3.56971	0.58645	-3.5218	2.40885	0.0797	-3.284
C	2.01819	-0.59322	-8.22112	1.71259	-0.10842	-8.31582
O	4.65153	1.19613	-2.99726	2.53829	1.09603	-2.40979
O	2.82743	-0.108	-2.84069	2.0762	-1.04279	-2.92982
C	4.84685	1.0252	-1.57933	2.29791	0.77014	-1.02568
O	-1.82548	-4.75641	-4.58595	-1.89478	-2.20658	-4.3871
O	-3.23567	-3.50916	-3.34776	-0.62296	-2.33743	-2.52962
C	-4.04882	-4.68297	-3.13591	-1.35164	-1.22565	-1.9708

H	-1.84839	-1.91426	-5.21397	0.70439	-4.03135	-3.58228
H	-1.60421	-1.70838	-3.48313	-0.75867	-4.74246	-4.27506
H	0.24392	-3.30129	-5.33788	-0.42777	-3.33494	-6.34256
H	0.15712	-0.84953	-5.83872	2.24804	-2.70799	-5.00344
H	0.62907	-0.49596	-4.182	2.01044	-2.61108	-6.73984
H	2.79414	-1.71849	-4.54211	0.19365	-0.91285	-6.39391
H	2.30605	-2.08364	-6.18841	0.48481	-0.95168	-4.66617
H	3.05271	1.8304	-8.70477	3.71772	1.6679	-8.22432
H	4.16026	3.40587	-7.14084	4.89484	2.60746	-6.2537
H	4.40735	2.78386	-4.7519	4.23478	1.87683	-3.97253
H	1.82737	-3.33652	-3.41308	1.333	-4.8352	-7.27498
H	0.28333	-4.04204	-2.93284	0.23605	-5.74477	-6.21795
H	0.69172	-2.3866	-2.44043	1.8448	-5.27792	-5.63711
H	2.59754	-1.51764	-8.34021	1.74801	-1.19977	-8.40156
H	1.00465	-0.88748	-7.92772	0.65305	0.17725	-8.31233
H	1.95708	-0.1121	-9.20181	2.16725	0.31316	-9.21719
H	5.74155	1.60046	-1.34066	2.98279	-0.01364	-0.69228
H	3.98616	1.40959	-1.02582	2.47888	1.69429	-0.47656
H	4.99783	-0.03085	-1.34109	1.26431	0.44166	-0.88886
H	-4.87821	-4.3519	-2.51069	-0.94423	-1.08625	-0.9696
H	-4.41966	-5.06686	-4.08981	-1.19344	-0.32879	-2.57551
H	-3.47143	-5.45734	-2.62439	-2.41911	-1.45405	-1.91871
		Con f. 1- 2o				
C	-0.90485	-3.77975	-5.06673			
C	-0.08873	-3.6594	-3.8069			
C	-0.1135	-2.22646	-3.20841			
C	0.41317	-1.15359	-4.17333			
C	1.84352	-1.41644	-4.68449			
C	2.43114	-0.21145	-5.38423			
C	2.22513	-0.04594	-6.77291			
C	2.7537	1.07488	-7.42246			
C	3.49484	2.03485	-6.73491			
C	3.72017	1.87437	-5.37512			
C	3.18635	0.76668	-4.6948			
C	-1.50455	-1.8471	-2.68946			
C	3.43521	0.67399	-3.22935			
C	1.45408	-1.06194	-7.58225			
O	4.5872	1.27812	-2.87537			
O	2.70812	0.13839	-2.40361			
C	4.88114	1.29766	-1.46441			
O	-0.48457	-3.58473	-6.19814			
O	-2.18583	-4.10191	-4.8165			
C	-3.05975	-4.17068	-5.96334			
H	-0.47109	-4.36379	-3.0622			
H	0.93774	-3.94079	-4.05473			
H	0.57225	-2.26129	-2.35095			
H	-0.27066	-1.05029	-5.02512			
H	0.39801	-0.19419	-3.64543			
H	2.47277	-1.7056	-3.83968			
H	1.82934	-2.26139	-5.37698			
H	2.58118	1.19235	-8.48936			
H	3.89462	2.89863	-7.25811			
H	4.29573	2.61045	-4.82595			
H	-1.46525	-0.88691	-2.16197			
H	-1.88987	-2.60091	-1.99273			
H	-2.22636	-1.74756	-3.50853			
H	1.95141	-2.03976	-7.58482			
H	0.44406	-1.22181	-7.19208			
H	1.3652	-0.73193	-8.62161			
H	4.98596	0.27845	-1.08362			
H	5.82497	1.83577	-1.37449			
H	4.09114	1.81758	-0.91619			
H	-3.09512	-3.20358	-6.47138			
H	-2.71573	-4.94147	-6.65763			
H	-4.04106	-4.42802	-5.56466			
		Conf.1 **-3**a			Conf.1 **-3**b	
C	-0.7387	-0.5216	1.1624	0.3844	2.3709	3.6779
C	-1.9171	0.0793	1.4062	-0.9472	2.7913	3.6705
C	-2.8321	-0.7434	0.8733	-1.4989	2.3185	4.8751
C	-2.1297	-1.8013	0.4389	-0.5064	1.6241	5.5472
O	-0.93	-1.6198	0.6432	0.6419	1.688	4.8285
C	0.6481	0.0439	1.3032	1.5486	2.5038	2.7074
C	1.7355	-0.6206	0.4238	2.423	1.2312	2.6046
C	1.3558	-0.9085	-1.0565	1.6504	0.0065	2.0333
C	0.674	0.1668	-1.931	1.9971	-1.3179	2.7402
C	-0.8304	-0.0244	-2.0385	1.4182	-1.3773	4.1554
C	-1.4128	-1.105	-2.5807	1.9728	-1.1202	5.3473
C	-2.9139	-1.0365	-2.4669	0.9427	-1.2786	6.427
O	-3.1147	0.2319	-1.8836	-0.1931	-1.7426	5.7218
C	-1.8693	0.7562	-1.6391	0.0291	-1.7558	4.3954
C	-2.5493	-2.6662	-0.527	-0.5284	0.8848	6.8056
C	-3.523	-2.1487	-1.5889	0.6781	0.0321	7.2238
C	3.0814	0.0799	0.6251	3.7582	1.4704	1.8964
C	3.5836	0.1723	2.0512	3.7011	2.0478	0.4892
C	3.8385	0.5839	-0.3646	4.9352	1.1888	2.4839
O	-2.0774	-3.779	-0.6494	-1.5044	0.9235	7.5533
O	-1.7637	1.8298	-1.0824	-0.8311	-2.0392	3.5657
O	1.0652	1.4473	-1.4955	1.5515	-2.3855	1.9034
C	0.9876	2.4337	-2.5025	2.0583	-3.6757	2.2345
H	-3.3805	-1.0249	-3.48	1.2586	-2.0598	7.1216
C	-2.1864	1.2946	1.9578	-1.6184	3.5559	2.623
O	-1.2902	1.9743	2.4169	-1.0623	3.9418	1.5941
O	-3.4819	1.7457	1.9966	-2.9143	3.7822	2.9298
C	-3.7128	3.0713	2.4105	-3.7468	4.5113	2.0367
H	-3.9041	-0.5478	0.7494	-2.5202	2.4469	5.2105
H	0.9044	-0.0392	2.3849	2.1713	3.3367	3.0345
H	0.6326	1.1234	1.0306	1.1938	2.7786	1.7151

H	1.9076	-1.6371	0.8693	2.6908	1.0002	3.6371
H	2.2816	-1.2598	-1.572	0.5718	0.154	2.1005
H	0.7359	-1.8322	-1.1096	1.839	-0.0934	0.9647
H	1.0603	0.0218	-2.9711	3.0865	-1.3752	2.8128
H	-0.8853	-1.9624	-3.0189	2.9888	-0.8099	5.5476
H	-3.7663	-3.007	-2.2613	1.5604	0.6697	7.2216
H	-4.4944	-1.842	-1.1413	0.513	-0.2298	8.2678
H	4.6404	0.5148	2.1133	4.6963	2.1433	0.053
H	2.9793	0.8982	2.6391	3.1162	1.4129	-0.1756
H	3.5378	-0.8226	2.5486	3.2577	3.0434	0.4969
H	3.5603	0.5469	-1.4249	4.9872	0.7797	3.4827
H	4.8012	1.0704	-0.1486	5.8737	1.3621	1.9769
H	1.1777	3.4221	-2.0278	1.7561	-3.9898	3.2345
H	1.7725	2.2524	-3.2704	1.67	-4.4099	1.5286
H	-0.0165	2.4474	-2.9777	3.1474	-3.7032	2.1757
H	-4.8012	3.2773	2.3036	-3.3619	5.5195	1.8744
H	-3.4305	3.1942	3.48	-3.8184	4.0115	1.0693
H	-3.1515	3.779	1.7602	-4.7524	4.5957	2.4488
		Conf.1 -**3c**			Conf.1 -**3d**	
C	0.43118	2.36376	3.68978	0.3818	2.3389	3.6753
C	-0.90585	2.73101	3.68759	-0.9459	2.7715	3.6571
C	-1.46708	2.26594	4.91463	-1.4994	2.3416	4.877
C	-0.45692	1.64144	5.59264	-0.5122	1.659	5.5686
O	0.70321	1.71355	4.8426	0.6352	1.6898	4.8462
C	1.55701	2.52573	2.73084	1.5449	2.4299	2.6986
C	2.40615	1.23358	2.61696	2.4091	1.1477	2.6371
C	1.55438	0.06404	2.09376	1.6264	-0.0894	2.1088
C	1.97318	-1.27557	2.7183	1.9785	-1.3956	2.8595
C	1.40688	-1.37151	4.10893	1.3952	-1.3976	4.27
C	1.98867	-1.13457	5.28938	1.9488	-1.1115	5.456
C	0.97678	-1.20564	6.3902	0.9138	-1.2275	6.5363
O	-0.25572	-1.55005	5.70844	-0.2245	-1.7003	5.8413
C	-0.03157	-1.60706	4.35976	0.0007	-1.7527	4.5162
C	-0.44636	0.89083	6.83788	-0.5386	0.9592	6.8495
C	0.7964	0.098	7.22183	0.6609	0.1093	7.2928
C	3.71038	1.4779	1.8671	3.7445	1.3508	1.9179
C	4.56993	2.59526	2.40928	3.6897	1.8908	0.4959
C	4.13785	0.73576	0.83925	4.9198	1.0696	2.5088
O	-1.44653	0.88555	7.55656	-1.513	1.0301	7.5971
O	-0.93707	-1.82383	3.57854	-0.8656	-2.0354	3.6921
O	1.48666	-2.31551	1.87624	1.5421	-2.5499	2.1409
C	1.9364	-3.60774	2.2633	2.3507	-2.9363	1.0327
H	1.20578	-2.02862	7.07393	1.2195	-1.9912	7.2545
C	-1.5959	3.45229	2.61343	-1.6119	3.5092	2.5872
O	-1.07243	3.85789	1.58407	-1.0539	3.8582	1.5461
O	-2.90292	3.60133	2.88401	-2.9054	3.7563	2.8883
C	-3.68054	4.29212	1.88675	-3.7327	4.4645	1.9738
H	-2.49139	2.36206	5.24337	-2.5188	2.49	5.21
H	2.18348	3.3552	3.07933	2.1749	3.2684	2.9965
H	1.14873	2.81703	1.76072	1.1901	2.6743	1.6984
H	2.71025	0.99602	3.6461	2.6777	0.9503	3.6763
H	0.49603	0.23355	2.31883	0.5497	0.064	2.1898
H	1.61792	-0.01812	1.00574	1.791	-0.1995	1.0396
H	3.06961	-1.33457	2.77536	3.0637	-1.4562	2.9799
H	3.0306	-0.89964	5.47513	2.9684	-0.81	5.6509
H	1.69028	0.7209	7.11568	1.5486	0.739	7.2703
H	0.67937	-0.15966	8.27573	0.4949	-0.1192	8.3444
H	4.14545	3.58093	2.18064	3.2608	2.8926	0.4785
H	4.65607	2.53174	3.50218	3.0931	1.247	-0.1496
H	5.57695	2.5623	1.98208	4.6844	1.9604	0.0535
H	3.57256	-0.0902	0.4204	4.9701	0.6867	3.518
H	5.10332	0.93296	0.37852	5.8587	1.2172	1.9943
H	1.54336	-3.90321	3.24602	1.9456	-3.8478	0.593
H	1.56674	-4.30781	1.50939	2.3745	-2.1805	0.2478
H	3.03477	-3.65411	2.2928	3.3755	-3.1485	1.3412
H	-3.29724	5.30517	1.73926	-3.3404	5.4645	1.7815
H	-3.65899	3.74478	0.94082	-3.8085	3.9367	1.0218
H	-4.6949	4.32359	2.28446	-4.7375	4.5687	2.3835
		Conf.1 -**3e**			Conf.2 -**3a**	
C	0.2013	2.3473	3.7295	8.08387	-6.60818	1.6323
C	-1.1379	2.7516	3.8211	8.02738	-5.22151	1.64778
C	-1.6029	2.237	5.0421	8.75685	-4.78862	2.79598
C	-0.5589	1.5515	5.6396	9.22024	-5.91696	3.41466
O	0.5392	1.6478	4.85	8.81693	-7.02268	2.68678
C	1.3097	2.5114	2.6966	7.50281	-7.68558	0.78256
C	2.2437	1.283	2.5738	6.9528	-8.85104	1.64149
C	1.5121	0.0189	2.0357	5.77949	-8.37927	2.53513
C	1.9626	-1.2857	2.72	5.67978	-9.17304	3.84009
C	1.4532	-1.3758	4.1596	6.91848	-8.98489	4.68314
C	2.0474	-1.0892	5.3252	7.45937	-7.84793	5.13689
C	1.0746	-1.2923	6.4493	8.748	-8.13707	5.84285
O	-0.0661	-1.8168	5.7964	8.83064	-9.5841	5.83942
C	0.0972	-1.8235	4.4616	7.78794	-10.0942	5.11655
C	-0.489	0.7943	6.8861	9.88701	-6.15335	4.68833
C	0.7807	0.0106	7.248	10.0193	-7.59903	5.13861
C	3.5395	1.5822	1.8168	6.55454	-10.049	0.7974
C	3.4042	2.1445	0.409	5.69548	-9.79898	-0.41509
C	4.749	1.3641	2.3638	6.93845	-11.2798	1.15444
O	-1.4348	0.7685	7.6723	10.28853	-5.21191	5.3718
O	-0.7833	-2.1538	3.6714	7.67689	-11.2907	4.9187
O	1.5357	-2.3748	1.9014	4.51079	-8.70815	4.51735
C	2.124	-3.6368	2.2054	4.17233	-9.49821	5.65156
H	1.4587	-2.0517	7.1336	8.728	-7.80706	6.8858
C	-1.9435	3.535	2.8868	7.35436	-4.30216	0.72232
O	-3.1391	3.7722	3.069	7.33395	-3.08914	0.87838
O	-1.2303	3.9644	1.828	6.76831	-4.92471	-0.31156
C	-1.8489	4.7446	0.8124	6.07437	-4.07613	-1.25114

H	-2.6039	2.3425	5.4415	8.89376	-3.76907	3.12641
H	1.904	3.3823	2.9739	8.28271	-8.068	0.10999
H	0.9009	2.7384	1.714	6.72406	-7.24745	0.15699
H	2.5602	1.0728	3.5968	7.76877	-9.17461	2.29384
H	0.4319	0.1088	2.157	4.82771	-8.46955	2.00119
H	1.653	-0.0691	0.9588	5.89753	-7.32082	2.79459
H	3.0557	-1.2846	2.7419	5.5706	-10.2443	3.62052
H	3.0543	-0.727	5.4795	7.07445	-6.84303	5.011
H	1.6264	0.6941	7.1968	10.20365	-8.23805	4.27099
H	0.6831	-0.2484	8.3011	10.86424	-7.67126	5.82674
H	2.9111	3.1163	0.4258	6.24382	-9.23597	-1.18178
H	4.3777	2.2871	-0.0622	5.35989	-10.739	-0.86388
H	2.8305	1.4752	-0.2313	4.80864	-9.20298	-0.16435
H	4.8566	0.9663	3.3627	7.55846	-11.4517	2.03174
H	5.6593	1.5796	1.8225	6.63668	-12.1584	0.58776
H	1.8846	-3.9666	3.2172	4.94535	-9.44537	6.4306
H	1.7429	-4.39	1.5157	4.02397	-10.5512	5.37252
H	3.2093	-3.6071	2.0969	3.23744	-9.09539	6.05032
H	-2.6671	4.1984	0.3401	5.66221	-4.75422	-1.998
H	-2.2474	5.6756	1.219	5.27424	-3.52815	-0.74759
H	-1.1213	4.9968	0.041	6.77283	-3.37486	-1.7142
		Conf.2 **-3b**			Conf.2 **-3c**	
C	8.10594	-6.75426	1.54346	8.07199	-6.60807	1.62146
C	8.02199	-5.37071	1.49473	8.05237	-5.22046	1.6381
C	8.73712	-4.87122	2.626	8.78424	-4.80793	2.79261
C	9.21778	-5.96297	3.29674	9.21375	-5.94892	3.41313
O	8.83967	-7.10837	2.61738	8.78732	-7.04191	2.68016
C	7.53217	-7.86931	0.74147	7.46031	-7.67724	0.78061
C	6.96814	-8.98589	1.65613	6.89043	-8.82252	1.6576
C	5.79655	-8.45969	2.52052	5.73435	-8.32197	2.54825
C	5.68293	-9.1869	3.86288	5.60631	-9.12746	3.84365
C	6.91599	-8.96161	4.70529	6.85423	-8.98455	4.68246
C	7.45358	-7.80497	5.1103	7.42166	-7.86714	5.15236
C	8.74037	-8.06011	5.83267	8.71496	-8.19375	5.83265
O	8.82464	-9.50567	5.89447	8.77111	-9.64208	5.79744
C	7.78617	-10.0491	5.19026	7.71002	-10.1178	5.07804
C	9.87225	-6.13091	4.58743	9.87031	-6.2093	4.68744
C	10.0126	-7.55211	5.10763	9.98483	-7.66268	5.12
C	6.56361	-10.2221	0.87301	6.52037	-10.0305	0.80497
C	5.70588	-10.0276	-0.35038	7.44268	-11.2121	0.93804
C	6.94355	-11.4356	1.28884	5.46624	-10.0306	-0.01694
O	10.25287	-5.15391	5.23197	10.28187	-5.28175	5.38387
O	7.67879	-11.2533	5.04369	7.57574	-11.3073	4.85307
O	4.51096	-8.68153	4.50497	4.4554	-8.63016	4.52692
C	4.15477	-9.41235	5.67275	4.09646	-9.41272	5.66023
H	8.71708	-7.68333	6.85957	8.71678	-7.88605	6.8825
C	7.30155	-4.5932	0.48062	7.41221	-4.28553	0.70536
O	6.72303	-5.0655	-0.48895	7.41702	-3.07227	0.86092
O	7.34936	-3.27687	0.74244	6.82695	-4.89572	-0.33632
C	6.66897	-2.42053	-0.19917	6.16514	-4.03271	-1.28572
H	8.85662	-3.83794	2.91754	8.94597	-3.79248	3.12458
H	8.31657	-8.28999	0.09778	8.22565	-8.08452	0.10653
H	6.76391	-7.45488	0.08626	6.6814	-7.23654	0.15654
H	7.77962	-9.28141	2.32752	7.7085	-9.13668	2.31212
H	4.84583	-8.56622	1.9877	4.78403	-8.37123	2.00872
H	5.92443	-7.39098	2.72868	5.89054	-7.27301	2.82589
H	5.569	-10.2674	3.69708	5.46134	-10.193	3.6139
H	7.06685	-6.80758	4.93826	7.05373	-6.85306	5.05159
H	10.20293	-8.23286	4.27399	10.15132	-8.29377	4.24314
H	10.85513	-7.58586	5.80162	10.83666	-7.75444	5.79729
H	4.81899	-9.42019	-0.12908	7.4751	-11.5598	1.97984
H	6.25572	-9.50083	-1.14129	7.13773	-12.0472	0.29939
H	5.37037	-10.9872	-0.75585	8.47257	-10.9332	0.67373
H	7.56448	-11.5673	2.17248	4.80496	-9.17202	-0.11128
H	6.63843	-12.3395	0.76531	5.226	-10.8956	-0.63119
H	4.00718	-10.4778	5.44539	3.91212	-10.4592	5.3781
H	3.21563	-8.98782	6.03758	4.87459	-9.38806	6.43543
H	4.91694	-9.32172	6.45905	3.17724	-8.98071	6.06472
H	5.60366	-2.66243	-0.22936	6.88539	-3.34737	-1.73921
H	6.81986	-1.40744	0.17321	5.7484	-4.70197	-2.03803
H	7.10426	-2.53124	-1.19546	5.37042	-3.46668	-0.79363
		Conf.2 **-3d**			Conf.2 **-3e**	
C	8.10617	-6.76085	1.53554	8.06351	-6.74825	1.57049
C	8.04422	-5.37609	1.49001	7.99665	-5.36474	1.49855
C	8.75522	-4.89066	2.63028	8.75956	-4.85469	2.59323
C	9.21229	-5.99155	3.30283	9.24928	-5.94069	3.26672
O	8.8241	-7.12851	2.61579	8.83181	-7.09294	2.62285
C	7.51524	-7.87181	0.73806	7.45169	-7.86874	0.80489
C	6.92034	-8.96422	1.66594	6.93952	-8.98389	1.74996
C	5.75901	-8.40576	2.5136	5.80021	-8.4669	2.66317
C	5.61157	-9.13426	3.85239	5.75332	-9.20651	4.00841
C	6.85207	-8.95311	4.69476	7.00109	-8.94323	4.80632
C	7.4142	-7.8149	5.11841	7.54584	-7.77281	5.1581
C	8.701	-8.10789	5.82586	8.85482	-8.00135	5.84872
O	8.7608	-9.55619	5.85421	8.95041	-9.44438	5.94532
C	7.70772	-10.0657	5.14658	7.89517	-10.0127	5.2883
C	9.85384	-6.17623	4.59787	9.94973	-6.09378	4.5349
C	9.97854	-7.60378	5.10608	10.10154	-7.50749	5.07227
C	6.54801	-10.2126	0.87532	6.51436	-10.2305	0.99435
C	7.45804	-11.3931	1.08231	5.60373	-10.0552	-0.19333
C	5.50202	-10.2479	0.04392	6.92107	-11.4373	1.40483
O	10.23836	-5.20846	5.2539	10.36219	-5.10955	5.1482
O	7.57911	-11.2642	4.97155	7.79518	-11.2205	5.17046
O	4.45896	-8.58683	4.49321	4.64004	-8.7736	4.80153
C	4.07485	-9.30366	5.66097	3.41807	-9.41488	4.45371
H	8.68958	-7.75495	6.86138	8.86006	-7.59777	6.86559
C	7.34535	-4.58604	0.47076	7.25414	-4.59569	0.4942

O	6.7689	-5.04831	-0.50477	6.61873	-5.07778	-0.43408
O	7.41059	-3.27092	0.73522	7.35299	-3.2744	0.71398
C	6.75179	-2.4032	-0.21128	6.65784	-2.42372	-0.22174
H	8.88768	-3.86	2.92554	8.90363	-3.81807	2.86083
H	8.29471	-8.31843	0.10669	8.20331	-8.28819	0.12215
H	6.75516	-7.45895	0.07202	6.6496	-7.459	0.18828
H	7.72711	-9.25099	2.34697	7.78227	-9.26645	2.38783
H	4.81422	-8.47958	1.96736	4.83635	-8.57303	2.15521
H	5.92	-7.3435	2.73202	5.93024	-7.39888	2.87266
H	5.4579	-10.21	3.68404	5.67535	-10.2881	3.83616
H	7.04502	-6.80725	4.96923	7.15129	-6.78261	4.96479
H	10.16354	-8.27853	4.26654	10.26207	-8.20165	4.24343
H	10.8203	-7.65194	5.80012	10.96702	-7.53293	5.73788
H	7.15257	-12.2588	0.48589	4.72056	-9.45482	0.06026
H	8.49344	-11.138	0.81558	6.11395	-9.52925	-1.01093
H	7.47491	-11.6839	2.14165	5.26138	-11.0214	-0.57649
H	4.84941	-9.39009	-0.10305	7.57908	-11.5558	2.26319
H	5.2599	-11.1429	-0.52495	6.60208	-12.3483	0.90245
H	3.15628	-8.83926	6.02948	3.49399	-10.505	4.57602
H	3.88033	-10.3612	5.43192	3.11205	-9.19698	3.42176
H	4.84291	-9.24783	6.44468	2.65608	-9.02919	5.13665
H	7.19376	-2.51986	-1.20394	5.58521	-2.63164	-0.19805
H	5.68309	-2.62822	-0.25072	6.85684	-1.40588	0.11317
H	6.91548	-1.39288	0.16326	7.04451	-2.57659	-1.23244
	Conf.1 -4a			Conf.1 -4b		
C	2.89081	-0.16275	-0.483	2.87689	0.27548	-0.70778
C	2.72931	1.12661	-0.8354	2.52237	1.528	-1.05109
C	1.69874	1.71909	-0.00361	1.47047	1.98586	-0.16493
O	1.306	0.77915	0.93056	1.25309	1.00645	0.78544
C	1.84448	-0.53461	0.56046	1.94699	-0.22578	0.39285
C	1.18403	2.95654	-0.12626	0.79746	3.14804	-0.25302
C	0.18921	3.6758	0.76018	-0.22969	3.73833	0.68952
C	-1.14937	4.00758	0.03234	-1.64097	3.88668	0.04246
C	-2.02747	2.91336	-0.5751	-2.40124	2.68377	-0.51567
C	-2.52436	1.79076	0.31194	-2.69888	1.51166	0.39585
C	-2.11442	0.51034	-0.30309	-2.15214	0.29271	-0.23783
C	-2.71063	-0.85535	-0.06751	-2.55123	-1.1367	0.03031
C	0.74068	-1.37727	-0.08041	0.93386	-1.2185	-0.18103
C	-0.44641	-1.79319	0.81551	-0.13897	-1.77516	0.78294
C	-1.70379	-2.01931	-0.08681	-1.40885	-2.16464	-0.04295
C	-0.16793	-3.02585	1.66121	0.3448	-2.95771	1.60608
C	-0.47493	-3.03597	2.96262	0.17548	-2.9741	2.93235
C	0.42917	-4.22825	0.97717	0.98928	-4.10645	0.87462
O	-1.43809	1.03913	0.85199	-1.49705	0.91487	0.88184
O	-1.30119	2.23206	-1.65487	-1.65108	2.09663	-1.63359
C	-1.42491	0.89572	-1.59332	-1.58663	0.75697	-1.56229
O	-3.68073	-1.05184	-1.12247	-3.54467	-1.46692	-0.96822
O	-1.04812	0.16794	-2.4872	-1.15978	0.07937	-2.47304
O	2.29758	-1.17366	1.70845	2.53867	-0.78094	1.52142
C	3.41193	-0.55804	2.36409	3.59627	-0.01432	2.10892
C	-4.87029	-1.66639	-0.91476	-4.64933	-2.19692	-0.68138
C	-5.25166	-2.08487	0.47836	-4.90486	-2.61844	0.73903
O	-5.58314	-1.82893	-1.88813	-5.39311	-2.45841	-1.60904
O	-0.03567	2.99566	1.99803	-0.28875	3.04496	1.93898
C	0.8186	5.02045	1.16666	0.23993	5.15737	1.05682
C	3.80931	-1.15466	-1.04226	3.91501	-0.5138	-1.37654
O	3.85085	-2.32684	-0.68554	4.59196	-0.11498	-2.31524
O	4.61369	-0.64123	-1.98832	4.05314	-1.73612	-0.8321
C	5.5531	-1.56041	-2.58393	5.05361	-2.58623	-1.43015
H	3.25921	1.66857	-1.60853	2.93919	2.12441	-1.85328
H	1.58659	3.54691	-0.94475	1.07514	3.78073	-1.09166
H	-0.92606	4.70706	-0.78096	-1.55856	4.60425	-0.78142
H	-1.77732	4.54917	0.74988	-2.29166	4.34556	0.79635
H	-2.8887	3.38973	-1.05252	-3.34176	3.04026	-0.94559
H	-3.40348	1.90528	0.93741	-3.55366	1.51219	1.06423
H	-3.20281	-0.80663	0.90483	-2.99079	-1.14472	1.02853
H	1.20797	-2.26377	-0.51719	1.49126	-2.04081	-0.63713
H	0.37838	-0.79008	-0.9235	0.45026	-0.69908	-1.00734
H	-0.66502	-0.97895	1.50826	-0.41496	-0.99084	1.49027
H	-2.25393	-2.90483	0.24653	-1.82541	-3.108	0.32467
H	-1.40683	-2.20035	-1.12378	-1.15139	-2.31896	-1.09478
H	-0.8871	-2.15906	3.45821	-0.27111	-2.13414	3.46087
H	-0.32354	-3.9195	3.57955	0.48016	-3.82564	3.53757
H	-0.09937	-4.46708	0.04485	1.98618	-3.82773	0.50876
H	1.47805	-4.04521	0.70869	1.10538	-4.97741	1.52714
H	0.39862	-5.10971	1.62528	0.40258	-4.40807	-0.00285
H	3.55499	-1.11276	3.29365	3.84498	-0.51723	3.04572
H	4.32062	-0.63072	1.75556	4.48055	0.00357	1.46145
H	3.20475	0.49211	2.59291	3.27208	1.00992	2.31835
H	-6.19861	-2.62317	0.42509	-5.7899	-3.25554	0.75205
H	-4.4915	-2.72795	0.93198	-4.05478	-3.16563	1.15795
H	-5.37655	-1.20448	1.11924	-5.08638	-1.74041	1.36936
H	-0.21556	2.06226	1.79016	-0.35608	2.09423	1.74311
H	1.03286	5.63957	0.29057	0.31915	5.79155	0.16899
H	0.13394	5.56703	1.82474	-0.4713	5.61489	1.75349
H	1.75332	4.83949	1.70675	1.2203	5.10631	1.54095
H	6.10495	-0.97244	-3.3172	5.0145	-3.51691	-0.86452
H	6.23064	-1.9566	-1.82319	4.8178	-2.76658	-2.48195
H	5.0228	-2.38151	-3.07296	6.04112	-2.12624	-1.34348
	Conf.1 -4c			Conf.1 -4d		
C	2.90106	-0.24041	-0.4882	2.91126	0.16834	-0.70116
C	2.76599	1.0568	-0.82288	2.60415	1.43926	-1.02221
C	1.73955	1.6561	0.00823	1.5522	1.9124	-0.14493
O	1.30343	0.7037	0.90855	1.27119	0.91321	0.7665
C	1.84404	-0.61003	0.54566	1.94897	-0.32773	0.37366
C	1.26898	2.91403	-0.07875	0.93816	3.10895	-0.19965
C	0.29427	3.63787	0.82717	-0.06771	3.71721	0.75549

C	-1.03772	4.02068	0.11247	-1.47035	3.93874	0.11104
C	-1.93421	2.96336	-0.53264	-2.27018	2.7811	-0.48619
C	-2.47352	1.83164	0.31809	-2.63245	1.60072	0.39127
C	-2.0695	0.55824	-0.31319	-2.11768	0.3778	-0.26012
C	-2.70423	-0.79703	-0.13272	-2.57853	-1.0408	-0.04264
C	0.74377	-1.45312	-0.10684	0.92469	-1.29298	-0.23204
C	-0.4722	-1.84562	0.75465	-0.18846	-1.81849	0.69618
C	-1.72897	-1.98471	-0.17036	-1.477	-2.10773	-0.14727
C	-0.31095	-3.11049	1.58751	0.16607	-3.05304	1.51525
C	0.628	-4.04143	1.39165	1.15919	-3.90045	1.22884
C	-1.3267	-3.24885	2.69432	-0.72642	-3.26918	2.71145
O	-1.41862	1.04521	0.8739	-1.46757	0.94443	0.8917
O	-1.20585	2.29252	-1.61712	-1.52402	2.19236	-1.60497
C	-1.33931	0.95695	-1.57741	-1.50578	0.85047	-1.56102
O	-3.65255	-0.9335	-1.21717	-3.57749	-1.29469	-1.05822
O	-0.94218	0.23998	-2.47126	-1.08307	0.17877	-2.47802
O	2.29287	-1.2383	1.70231	2.51085	-0.89999	1.50968
C	3.39357	-0.60508	2.3641	3.55951	-0.14582	2.12838
C	-4.87065	-1.50345	-1.05472	-4.70795	-1.997	-0.8058
C	-5.29976	-1.94657	0.31648	-4.98439	-2.46906	0.59485
O	-5.56606	-1.6097	-2.0482	-5.4563	-2.19358	-1.74573
O	0.05697	2.93409	2.0494	-0.15679	2.99491	1.9867
C	0.95914	4.9556	1.26351	0.45815	5.10602	1.15964
C	3.81674	-1.23552	-1.04642	3.94521	-0.63395	-1.36083
O	3.84906	-2.40972	-0.69594	4.65064	-0.23597	-2.27879
O	4.63042	-0.72181	-1.98466	4.0451	-1.86662	-0.83232
C	5.56896	-1.64287	-2.57848	5.04139	-2.72877	-1.41968
H	3.31753	1.60159	-1.5787	3.0598	2.04105	-1.79884
H	1.6966	3.51565	-0.87598	1.2576	3.75584	-1.01217
H	-0.79938	4.74114	-0.67781	-1.35723	4.67632	-0.69133
H	-1.65504	4.55137	0.84712	-2.10638	4.39926	0.87628
H	-2.77663	3.47114	-1.01114	-3.18829	3.18529	-0.92237
H	-3.36852	1.94936	0.92002	-3.50329	1.6186	1.03831
H	-3.2185	-0.7669	0.82879	-3.02526	-1.06412	0.95225
H	1.20887	-2.34307	-0.53821	1.46762	-2.12376	-0.68896
H	0.40506	-0.86241	-0.95735	0.47651	-0.74863	-1.06274
H	-0.66419	-1.03671	1.46632	-0.4287	-1.03324	1.4194
H	-2.31513	-2.86694	0.10285	-1.93536	-3.05062	0.16508
H	-1.40786	-2.13462	-1.20494	-1.20809	-2.22612	-1.20066
H	1.40351	-3.95795	0.6363	1.84188	-3.75725	0.39712
H	0.66625	-4.9308	2.01718	1.33634	-4.77827	1.84664
H	-1.20158	-2.44147	3.42941	-0.60396	-2.44794	3.43139
H	-2.35447	-3.16723	2.31646	-1.78765	-3.27764	2.42878
H	-1.23162	-4.20581	3.21683	-0.50303	-4.21047	3.22328
H	3.54353	-1.16102	3.29188	3.79712	-0.67043	3.05613
H	4.30582	-0.65908	1.75886	4.45222	-0.11157	1.49321
H	3.16775	0.44044	2.59681	3.23164	0.87306	2.35768
H	-4.57851	-2.63935	0.76072	-4.15745	-3.06692	0.99016
H	-5.39672	-1.08312	0.98444	-5.13369	-1.61369	1.26347
H	-6.26888	-2.43882	0.22788	-5.89324	-3.07161	0.57873
H	-0.1704	2.01653	1.81771	-0.2778	2.05501	1.76499
H	1.19139	5.58769	0.40141	0.56388	5.75899	0.2884
H	0.28893	5.50606	1.93309	-0.23523	5.57362	1.8675
H	1.88803	4.7374	1.79995	1.4349	5.00308	1.64286
H	6.241	-2.04448	-1.81568	4.82556	-2.89043	-2.47881
H	5.03787	-2.46011	-3.07308	6.03579	-2.29015	-1.30514
H	6.12708	-1.05473	-3.30688	4.97122	-3.66588	-0.86783
	Conf.2 -4**a**			Conf.2 -4**b**		
C	2.94108	-0.50825	-0.41401	2.9977	0.04766	-0.69133
C	2.8691	0.76467	-0.84356	2.65684	1.28664	-1.08931
C	1.91086	1.48344	-0.02589	1.65163	1.81401	-0.18766
O	1.52295	0.65344	1.00889	1.51324	0.92671	0.86323
C	1.9012	-0.73154	0.68682	2.10199	-0.36838	0.48001
C	1.42853	2.71499	-0.27112	0.92419	2.92946	-0.37836
C	0.44436	3.54562	0.51972	-0.13717	3.55519	0.49695
C	-0.8354	3.89021	-0.30288	-1.51769	3.65828	-0.22187
C	-1.73982	2.79892	-0.87588	-2.25359	2.42002	-0.73428
C	-2.27737	1.7373	0.05765	-2.50434	1.27535	0.22193
C	-1.92496	0.41505	-0.49785	-1.97881	0.03756	-0.3934
C	-2.6029	-0.89841	-0.20077	-2.364	-1.38542	-0.06916
C	0.70512	-1.4767	0.07831	1.00206	-1.29584	-0.05572
C	-0.49201	-2.11866	0.85242	0.02159	-2.16399	0.79567
C	-1.70467	-2.146	-0.13529	-1.23209	-2.42835	-0.09899
C	-0.92399	-1.7363	2.26492	-0.36732	-1.87859	2.24371
C	-0.46757	-0.72976	3.01559	-0.04172	-0.81371	2.98203
C	-1.9661	-2.67504	2.83691	-1.17164	-2.99916	2.87007
O	-1.21691	0.96539	0.62222	-1.27609	0.70162	0.66753
O	-1.02754	2.04034	-1.91434	-1.51035	1.80698	-1.84429
C	-1.21377	0.71276	-1.80001	-1.44971	0.46618	-1.74513
O	-3.54822	-1.08997	-1.28238	-3.31875	-1.81386	-1.07056
O	-0.85479	-0.06889	-2.65458	-1.03263	-0.23299	-2.6435
O	2.34436	-1.37541	1.83081	2.7303	-0.93711	1.57462
C	3.54087	-0.84881	2.41705	3.86209	-0.22164	2.08502
C	-4.82451	-1.49394	-1.07911	-4.65784	-1.81801	-0.86155
C	-5.30795	-1.72549	0.32507	-5.19674	-1.45862	0.4947
O	-5.51904	-1.64361	-2.06781	-5.35891	-2.13832	-1.80377
O	0.11812	2.96627	1.78635	-0.25529	2.91643	1.77149
C	1.12644	4.88105	0.86672	0.30673	4.9928	0.82035
C	3.76272	-1.59598	-0.94541	3.97565	-0.80395	-1.37535
O	3.73998	-2.74457	-0.51853	4.62167	-0.4631	-2.35787
O	4.55902	-1.19674	-1.95212	4.09746	-2.00945	-0.79245
C	5.40885	-2.21229	-2.52482	5.03973	-2.91519	-1.40304
H	3.40614	1.21158	-1.67061	3.03726	1.82062	-1.95135
H	1.83818	3.2042	-1.15062	1.16384	3.48778	-1.27947
H	-0.53677	4.51925	-1.14878	-1.40598	4.32872	-1.0812
H	-1.46936	4.51032	0.34208	-2.20122	4.15439	0.47741
H	-2.579	3.28117	-1.38552	-3.20856	2.74127	-1.16035

H	-3.14448	1.91454	0.68534	-3.32214	1.29684	0.93443
H	-3.12432	-0.76485	0.74675	-2.81823	-1.37556	0.9209
H	1.13859	-2.30476	-0.49136	1.50897	-2.01671	-0.70476
H	0.33246	-0.79138	-0.67807	0.44452	-0.66088	-0.73862
H	-0.22107	-3.17798	0.95303	0.50461	-3.14844	0.85753
H	-2.37127	-2.97303	0.12759	-1.70064	-3.37195	0.19547
H	-1.33163	-2.35331	-1.14255	-0.90324	-2.55123	-1.1351
H	0.26818	-0.01618	2.67557	0.529	0.02218	2.60556
H	-0.84264	-0.59347	4.02829	-0.35577	-0.75645	4.02266
H	-2.93435	-2.56788	2.33158	-2.17445	-3.08176	2.43128
H	-1.66572	-3.72487	2.71874	-0.68362	-3.97161	2.71924
H	-2.127	-2.48162	3.90187	-1.29424	-2.84035	3.94578
H	4.41003	-1.02543	1.77338	4.70107	-0.25006	1.38049
H	3.44282	0.22237	2.61827	3.60342	0.81804	2.30767
H	3.67359	-1.38682	3.35777	4.14708	-0.73321	3.00648
H	-5.28132	-0.79523	0.9032	-4.89901	-0.44584	0.78315
H	-6.33526	-2.08844	0.27756	-6.28497	-1.5185	0.45569
H	-4.68413	-2.46248	0.84059	-4.82442	-2.15177	1.2571
H	-0.09714	2.03019	1.63467	-0.29324	1.95725	1.61751
H	1.42289	5.42189	-0.03683	0.42586	5.58578	-0.09122
H	0.43959	5.5103	1.44389	-0.43983	5.47711	1.45978
H	2.01862	4.69026	1.47142	1.26272	4.97091	1.35291
H	5.96908	-1.70783	-3.31197	4.99797	-3.8224	-0.80057
H	6.08854	-2.61056	-1.76705	4.74797	-3.12659	-2.43485
H	4.80469	-3.02103	-2.94378	6.04482	-2.48672	-1.38081
		Conf.2 -4c			Conf.3 -4a	
C	2.9713	-0.23424	-0.66879	-3.20245	-0.21406	0.08574
C	2.79009	1.04184	-1.05423	-3.36678	1.08587	-0.21634
C	1.83389	1.67407	-0.16759	-2.14642	1.80031	0.1091
O	1.55592	0.79198	0.8596	-1.28577	0.91814	0.73038
C	2.00658	-0.55708	0.477	-1.79604	-0.45636	0.62219
C	1.26248	2.87889	-0.34586	-1.84799	3.07418	-0.21356
C	0.27469	3.61964	0.52641	-0.55972	3.85583	0.01071
C	-1.07251	3.90025	-0.20814	0.58268	3.42865	-0.96567
C	-1.93836	2.76674	-0.75737	0.80531	1.93458	-1.01414
C	-2.35415	1.65201	0.17703	1.44513	1.25552	0.17053
C	-1.95884	0.36714	-0.43588	2.28394	0.18156	-0.34873
C	-2.52764	-0.99481	-0.12697	2.48654	-1.16986	0.31245
C	0.82516	-1.35737	-0.08894	-0.93896	-1.2772	-0.37544
C	-0.27372	-2.10235	0.73684	0.09536	-2.27175	0.18959
C	-1.54681	-2.18038	-0.16757	1.25671	-1.70374	1.05629
C	-0.63109	-1.79442	2.18808	0.65618	-3.18616	-0.89456
C	-0.18876	-0.78451	2.94256	0.62051	-2.89994	-2.20114
C	-1.56467	-2.82017	2.79614	1.30745	-4.45171	-0.3944
O	-1.21579	0.9346	0.65319	2.87556	1.37476	0.23411
O	-1.24171	2.07844	-1.85346	1.65841	1.53217	-2.14239
C	-1.34975	0.73988	-1.77004	2.319	0.38454	-1.84378
O	-3.545	-1.22401	-1.13314	3.5224	-1.05679	1.31873
O	-1.00455	0.00317	-2.66898	2.84953	-0.30189	-2.68821
O	2.54539	-1.19841	1.57998	-1.73202	-1.05688	1.87544
C	3.72977	-0.60617	2.12676	-2.55464	-0.47906	2.89662
C	-4.76069	-1.74328	-0.83871	4.77382	-0.82655	0.86706
C	-5.1013	-2.05598	0.59179	5.74518	-0.68677	2.00167
O	-5.52426	-1.9209	-1.77025	5.04331	-0.73786	-0.31778
O	0.06336	2.98498	1.79067	-0.82078	5.24812	-0.24307
C	0.88681	4.98803	0.87449	-0.13363	3.82241	1.48297
C	3.86808	-1.18292	-1.33642	-0.14582	1.44545	-1.2067
O	4.57622	-0.90626	-2.29616	-4.15413	-1.31596	-0.07518
O	3.83953	-2.39979	-0.76547	-3.90699	-2.47582	0.23151
C	4.69418	-3.39831	-1.36002	-5.32895	-0.91669	-0.58747
H	3.25606	1.53962	-1.89565	-6.32239	-1.94917	-0.76361
H	1.59097	3.41955	-1.22929	-4.23754	1.55491	-0.65642
H	-0.86774	4.56914	-1.05158	-2.64428	3.62505	-0.70614
H	-1.70449	4.46017	0.49156	0.29451	3.75197	-1.97292
H	-2.83403	3.20587	-1.2064	1.49921	3.9585	-0.68574
H	-3.18924	1.76057	0.86125	0.93889	1.17496	1.11799
H	-2.97778	-0.91749	0.86237	2.82314	-1.87624	-0.44852
H	1.26814	-2.13366	-0.72047	-1.63529	-1.87835	-0.96614
H	0.36245	-0.66904	-0.79039	-0.49315	-0.5691	-1.07537
H	0.0717	-3.14376	0.78132	-0.46575	-2.91446	0.87825
H	-2.13843	-3.05837	0.10938	1.62434	-2.52059	1.68501
H	-1.23234	-2.33008	-1.20464	0.873	-0.93979	1.73667
H	0.47504	-0.01348	2.58158	0.17096	-1.99522	-2.59799
H	-0.50053	-0.70649	3.98259	1.05257	-3.57941	-2.93265
H	-2.56756	-2.77493	2.35346	1.63329	-5.08732	-1.22369
H	-1.19609	-3.84183	2.63219	2.18653	-4.23585	0.22727
H	-1.67262	-2.66303	3.87367	0.61364	-5.02709	0.2331
H	4.57808	-0.70491	1.43983	-3.61902	-0.62508	2.68198
H	3.56903	0.45021	2.36278	-2.29601	-1.00673	3.81663
H	3.94252	-1.15779	3.04463	-2.34495	0.58846	3.01596
H	-5.11649	-1.14069	1.19405	6.76235	-0.62563	1.61232
H	-6.09032	-2.51501	0.6148	5.51146	0.22524	2.56306
H	-4.37091	-2.74047	1.03423	5.65274	-1.53351	2.68887
H	-0.09393	2.04028	1.62202	-1.04859	5.34016	-1.18452
H	1.08841	5.57321	-0.0276	-0.88214	4.35712	2.0764
H	0.19707	5.55201	1.51243	-0.06587	2.80579	1.86989
H	1.82628	4.84242	1.41689	0.83438	4.3184	1.60981
H	4.52405	-4.30443	-0.77886	-7.19224	-1.44248	-1.18119
H	4.421	-3.55705	-2.40619	-5.95618	-2.71321	-1.45393
H	5.74048	-3.09013	-1.29111	-6.56985	-2.40494	0.19844
		Conf.3 -4b			Conf.4 -4a	
C	-3.26661	0.05094	-0.06522	2.78957	-0.37376	0.4729
C	-3.33224	1.36085	-0.36163	2.62345	0.78118	1.14166
C	-2.08461	1.9942	0.01848	1.93625	1.73332	0.28896
O	-1.30447	1.05245	0.65783	1.71979	1.13321	-0.93641
C	-1.89585	-0.28641	0.5153	2.26465	-0.23182	-0.94551
C	-1.69614	3.25277	-0.26604	1.48218	2.94663	0.65931

C	-0.37529	3.94923	0.03599	0.59882	3.94456	-0.06577
C	0.78567	3.47366	-0.89547	-0.85003	3.92071	0.52186
C	0.91676	1.96992	-0.97601	-1.28462	2.51874	0.90145
C	1.46037	1.22068	0.21476	-1.327	1.49021	-0.19604
C	2.24995	0.10748	-0.30008	-2.46618	0.61802	0.06685
C	2.34184	-1.27121	0.32939	-2.49351	-0.86596	-0.16092
C	-1.05539	-1.14529	-0.46494	1.15971	-1.19681	-1.38869
C	-0.11011	-2.21477	0.11936	0.00458	-1.37593	-0.37068
C	1.06039	-1.74006	1.0283	-1.37114	-1.33856	-1.08776
C	0.41939	-3.15145	-0.96163	0.17333	-2.61013	0.50516
C	0.46408	-2.83732	-2.26149	0.36308	-2.47584	1.8235
C	0.94231	-4.47595	-0.46422	0.10804	-3.96743	-0.14588
O	2.89122	1.24461	0.34033	-2.47365	1.5455	-1.05868
O	1.79206	1.54344	-2.07825	-2.63651	2.48164	1.46744
C	2.36319	0.34743	-1.78588	-3.23746	1.29825	1.17313
O	3.35606	-1.25583	1.36442	-3.7972	-1.16056	-0.72491
O	2.88482	-0.35006	-2.62682	-4.24334	0.91987	1.72931
O	-1.90522	-0.89972	1.76377	3.24857	-0.32251	-1.94079
C	-2.73463	-0.28968	2.76008	4.41457	0.4891	-1.76221
C	4.63092	-1.08953	0.95254	-4.28274	-2.40215	-0.50884
C	5.57835	-1.04683	2.11498	-5.64119	-2.56663	-1.12405
O	4.93725	-0.97885	-0.22147	-3.67428	-3.25729	0.11092
O	-0.53919	5.36028	-0.19489	1.07164	5.26975	0.26115
C	-0.0253	3.85682	1.52571	0.59075	3.79742	-1.59091
H	-0.05341	1.54685	-1.22266	-0.62689	2.1266	1.68135
C	-4.34148	-0.91834	-0.3102	3.37281	-1.59337	1.04413
O	-5.41585	-0.64492	-0.82773	3.70659	-1.71507	2.21463
O	-4.01254	-2.14997	0.11418	3.4883	-2.57045	0.13187
C	-5.0051	-3.17795	-0.08661	3.94071	-3.8475	0.62554
H	-4.1585	1.88191	-0.82895	2.90922	0.98482	2.16608
H	-2.43488	3.86155	-0.77973	1.72489	3.23365	1.67952
H	0.56916	3.84	-1.90614	-0.85896	4.54332	1.4215
H	1.71855	3.93686	-0.55732	-1.54539	4.35348	-0.20452
H	0.90996	1.15068	1.13808	-0.41626	1.15741	-0.66613
H	2.64923	-1.98021	-0.44174	-2.41961	-1.35968	0.81212
H	-1.76382	-1.68929	-1.09506	0.78232	-0.79551	-2.33435
H	-0.53628	-0.4577	-1.13397	1.63744	-2.15009	-1.62007
H	-0.73461	-2.82604	0.78152	0.0404	-0.53087	0.31891
H	1.35837	-2.59226	1.64711	-1.33047	-0.66449	-1.94924
H	0.70722	-0.96805	1.71609	-1.62986	-2.32634	-1.47582
H	0.10855	-1.89046	-2.65531	0.40061	-1.49842	2.29862
H	0.87094	-3.53548	-2.98985	0.49142	-3.33871	2.47347
H	0.17496	-5.00625	0.11568	0.76995	-4.03592	-1.01776
H	1.25593	-5.11823	-1.29305	-0.90797	-4.18577	-0.49885
H	1.80441	-4.3452	0.20305	0.3908	-4.75571	0.55842
H	-2.48796	0.76913	2.8858	5.0113	0.35077	-2.66616
H	-3.79685	-0.39518	2.51292	4.15237	1.54768	-1.66126
H	-2.52422	-0.82568	3.68759	4.99761	0.17024	-0.89095
H	5.39723	-0.13125	2.68996	-6.33291	-1.83576	-0.69138
H	5.40415	-1.89808	2.7802	-5.58537	-2.37328	-2.20071
H	6.60758	-1.05312	1.75357	-6.00823	-3.57818	-0.94564
H	-0.71635	5.48808	-1.14316	1.94762	5.37045	-0.15101
H	-0.77043	4.42024	2.09639	1.61506	3.80512	-1.97947
H	-0.0356	2.82864	1.88708	0.11101	2.87694	-1.92804
H	0.96222	4.29193	1.71213	0.04708	4.64613	-2.01745
H	-5.22606	-3.28745	-1.15127	3.265	-4.20845	1.4049
H	-4.55685	-4.08987	0.30704	3.92099	-4.51262	-0.23733
H	-5.91879	-2.93255	0.46046	4.95563	-3.76325	1.02184
		Conf.4 -**4**b			Conf.4 -**4**c	
C	2.78268	-0.55849	0.21562	-2.66312	0.44442	0.30121
C	2.70442	0.56398	0.95243	-2.44176	1.6425	-0.26919
C	2.0145	1.58775	0.18565	-1.12736	2.11493	0.12166
O	1.69875	1.0585	-1.05084	-0.57211	1.17899	0.96994
C	2.18527	-0.322	-1.15863	-1.49357	0.06611	1.19249
C	1.63955	2.80063	0.63707	-0.48676	3.21379	-0.3213
C	0.76703	3.8726	0.01079	0.944	3.65475	-0.05824
C	-0.64399	3.89366	0.68462	1.93433	2.98099	-1.05756
C	-1.12837	2.49675	1.02209	1.68873	1.49754	-1.2225
C	-1.27269	1.52773	-0.11953	2.08389	0.53814	-0.12732
C	-2.43812	0.69644	0.15657	2.51463	-0.69035	-0.77915
C	-2.54917	-0.76957	-0.14757	2.19093	-2.07883	-0.28738
C	1.02586	-1.23643	-1.5664	-0.7614	-1.25485	0.91627
C	-0.09105	-1.39129	-0.50083	-0.40755	-1.55261	-0.55878
C	-1.49256	-1.24221	-1.14892	0.78643	-2.53603	-0.70987
C	0.04412	-2.67242	0.30994	-1.59628	-2.10493	-1.34064
C	0.3168	-2.61362	1.61939	-2.12017	-1.41506	-2.36006
C	-0.15115	-3.98793	-0.39762	-2.13913	-3.45671	-0.95127
O	-2.45511	1.68069	-0.91977	3.4729	0.15758	-0.09253
O	-2.4515	2.49213	1.65315	2.36362	0.96272	-2.41347
C	-3.12141	1.35314	1.33269	2.62278	-0.35695	-2.24671
O	-3.88845	-0.96749	-0.66917	2.27299	-2.06671	1.15424
O	-4.11571	0.99253	1.92051	2.91995	-1.09061	-3.16312
O	3.10158	-0.40101	-2.21612	-1.81252	0.00846	2.56122
C	4.31774	0.33901	-2.06344	-2.52026	1.13018	3.09899
C	-4.43069	-2.19196	-0.49563	3.49394	-2.25578	1.71129
C	-5.8172	-2.25514	-1.06541	3.427	-2.10108	3.20162
O	-3.84593	-3.10792	0.05628	4.49188	-2.51003	1.06428
O	1.32829	5.15605	0.36489	1.05387	5.05337	-0.39248
C	0.66143	3.7992	-1.51618	1.37263	3.47781	1.40334
H	-0.45275	2.03773	1.74847	0.6262	1.33885	-1.41365
C	3.3385	-1.8617	0.5908	-3.84742	-0.40783	0.17684
O	3.51661	-2.7782	-0.20071	-4.05168	-1.39697	0.86881
O	3.6219	-1.94344	1.89985	-4.68907	0.01697	-0.77849
C	4.13326	-3.21482	2.35207	-5.86703	-0.78997	-0.98288
H	3.05472	0.71079	1.96623	-3.10002	2.18293	-0.93725
H	1.95312	3.02632	1.65336	-1.03674	3.83254	-1.02455
H	-0.56744	4.47018	1.61134	1.78011	3.45453	-2.03276

H	-1.35623	4.39665	0.02267	2.96266	3.18011	-0.7383
H	-0.40242	1.17929	-0.65035	1.57133	0.53293	0.82099
H	-2.46003	-1.32077	0.79275	2.92414	-2.79263	-0.66883
H	0.62411	-0.81404	-2.49268	0.14375	-1.21922	1.52521
H	1.46353	-2.20291	-1.82451	-1.38113	-2.0553	1.32693
H	0.02623	-0.58399	0.22397	-0.13832	-0.61113	-1.04314
H	-1.45196	-0.52255	-1.97254	0.5852	-3.46568	-0.16715
H	-1.81886	-2.19175	-1.57945	0.86225	-2.80159	-1.77062
H	0.44244	-1.66444	2.13477	-1.73167	-0.44267	-2.65388
H	0.4232	-3.51363	2.22128	-2.96009	-1.79853	-2.93547
H	0.43402	-4.04364	-1.32355	-2.41368	-3.49749	0.10882
H	-1.20325	-4.13083	-0.67574	-1.39644	-4.2477	-1.11614
H	0.14176	-4.82605	0.24198	-3.02887	-3.70401	-1.53855
H	4.84611	0.23729	-3.01363	-2.57026	0.9581	4.17631
H	4.1197	1.39922	-1.87274	-1.98797	2.0684	2.90917
H	4.93855	-0.0706	-1.2589	-3.53724	1.19602	2.69657
H	-6.44976	-1.50189	-0.58363	2.59477	-2.68284	3.60924
H	-5.78649	-2.02649	-2.13625	4.36903	-2.41911	3.65043
H	-6.23663	-3.24993	-0.90965	3.24556	-1.04653	3.44053
H	2.18381	5.22764	-0.09362	0.52957	5.54539	0.26322
H	1.65989	3.77125	-1.96623	0.7045	4.05503	2.05337
H	0.1116	2.92375	-1.86574	1.33491	2.43988	1.73324
H	0.14246	4.69556	-1.86979	2.39316	3.85366	1.52897
H	3.40026	-4.00274	2.16141	-5.58267	-1.80659	-1.26581
H	5.07299	-3.44925	1.84587	-6.47555	-0.81306	-0.07528
H	4.29545	-3.0948	3.42308	-6.41003	-0.30566	-1.79423
		Conf.4 **-4d**			Conf.4 **-4e**	
C	-2.6727	0.62739	0.02279	2.78108	-0.56333	0.21286
C	-2.28668	1.80721	-0.49599	2.70881	0.56161	0.94631
C	-1.00019	2.17704	0.06273	2.02168	1.58601	0.17762
O	-0.61942	1.18831	0.94461	1.69938	1.05329	-1.05592
C	-1.63457	0.1334	1.01838	2.18261	-0.32817	-1.1612
C	-0.24949	3.24671	-0.26038	1.65615	2.80283	0.62423
C	1.15481	3.5933	0.1886	0.78346	3.87522	-0.00232
C	2.22464	2.909	-0.73035	-0.62797	3.89345	0.68301
C	1.91719	1.45625	-1.01927	-1.11801	2.49698	1.01722
C	2.1008	0.40851	0.05128	-1.27109	1.52935	-0.12433
C	2.51948	-0.81175	-0.62424	-2.4368	0.70019	0.15776
C	2.02855	-2.1956	-0.27509	-2.55202	-0.76567	-0.14584
C	-0.96201	-1.21514	0.72675	1.02103	-1.24085	-1.56628
C	-0.4674	-1.39383	-0.72019	-0.09515	-1.39115	-0.49944
C	0.6386	-2.47493	-0.86943	-1.4968	-1.2415	-1.14704
C	-1.57956	-1.72194	-1.71522	0.03828	-2.67038	0.31454
C	-2.48889	-2.67726	-1.49558	0.31045	-2.60868	1.62397
C	-1.54022	-0.93639	-2.99757	-0.15808	-3.9875	-0.38976
O	3.44207	-0.08519	0.22965	-2.45722	1.68448	-0.91842
O	2.70179	0.93908	-2.15007	-2.43831	2.49404	1.65633
C	2.83505	-0.40523	-2.04291	-3.11254	1.35726	1.33788
O	1.95734	-2.29347	1.16296	-3.89186	-0.96029	-0.66719
O	3.18373	-1.10868	-2.96493	-4.10489	0.99829	1.93017
O	-2.08669	0.03669	2.34297	3.0982	-0.4118	-2.21915
C	-2.78541	1.17468	2.8589	4.31503	0.3279	-2.07
C	3.09483	-2.61772	1.82428	-4.43646	-2.1839	-0.49486
C	2.87614	-2.57669	3.3077	-5.82237	-2.24448	-1.06636
O	4.13816	-2.89495	1.26393	-3.85385	-3.10119	0.05714
O	1.24808	5.0163	-0.03053	1.40561	5.15653	0.22715
C	1.40864	3.32371	1.67672	0.66731	3.79301	-1.52529
H	0.88023	1.38747	-1.35236	-0.44034	2.03354	1.73907
C	-3.93217	-0.04999	-0.31174	3.33343	-1.86722	0.5907
O	-4.62517	0.22926	-1.28048	3.50838	-2.78617	-0.19863
O	-4.25225	-0.99357	0.58689	3.61742	-1.94668	1.89984
C	-5.45443	-1.74192	0.31629	4.12598	-3.21826	2.35464
H	-2.82629	2.40535	-1.21962	3.06235	0.7102	1.95876
H	-0.67424	3.91606	-1.00274	1.97386	3.02287	1.6418
H	2.21589	3.44537	-1.68457	-0.55143	4.46152	1.61655
H	3.2171	3.02328	-0.27966	-1.34014	4.40672	0.02873
H	1.47047	0.39085	0.92632	-0.40421	1.17796	-0.65891
H	2.73085	-2.94795	-0.64042	-2.46421	-1.31676	0.79468
H	-0.12989	-1.29191	1.42963	0.61932	-0.81918	-2.4929
H	-1.67196	-2.0027	0.99102	1.45614	-2.2088	-1.82317
H	-0.04741	-0.44145	-1.04706	0.024	-0.58198	0.22307
H	0.28764	-3.42523	-0.45222	-1.45575	-0.52335	-1.97198
H	0.79961	-2.64495	-1.94035	-1.82447	-2.19139	-1.57576
H	-2.51905	-3.25432	-0.57501	0.43699	-1.65834	2.13701
H	-3.24337	-2.91757	-2.2408	0.41552	-3.50725	2.22822
H	-0.5493	-1.00801	-3.46737	0.42826	-4.04661	-1.31473
H	-1.71733	0.12943	-2.80109	-1.21001	-4.12956	-0.66887
H	-2.28913	-1.28451	-3.71583	0.13276	-4.82435	0.25246
H	-3.74379	1.3232	2.34921	4.93598	-0.07903	-1.26419
H	-2.96834	0.95627	3.91314	4.84283	0.22199	-3.02006
H	-2.18062	2.08423	2.77889	4.11793	1.389	-1.88342
H	1.97648	-3.13881	3.57626	-6.4539	-1.48918	-0.58648
H	3.74677	-2.98525	3.82248	-5.78952	-2.01727	-2.13745
H	2.7232	-1.5357	3.61571	-6.24428	-3.23813	-0.91006
H	2.13877	5.29063	0.24893	1.49838	5.27627	1.18836
H	0.67316	3.8691	2.27671	1.66142	3.74602	-1.97985
H	2.41043	3.68216	1.94121	0.09571	2.92637	-1.85767
H	1.34425	2.26701	1.93683	0.16093	4.69549	-1.88188
H	-6.32228	-1.07765	0.31569	3.39102	-4.00489	2.16618
H	-5.37332	-2.24999	-0.64774	5.06488	-3.45606	1.84848
H	-5.52979	-2.46754	1.12591	4.28906	-3.09623	3.4253
		Conf.1 **-6a**			Conf.1 **-6b**	
C	-2.5041	6.7137	-3.207	-2.5478	6.3915	-3.0914
C	-1.643	7.6187	-4.0836	-1.1268	5.8419	-2.9761
C	-0.4393	8.235	-3.3401	-0.0702	6.8713	-2.5131
C	-0.8615	8.9274	-2.052	-0.4258	7.495	-1.1747
C	-5.7487	7.1756	-1.1472	-5.0835	8.1846	-1.7416

C	-4.6169	6.3528	-1.8089	-4.904	6.689	-2.0992
C	-3.7137	7.1152	-2.7626	-3.4575	6.2126	-2.106
C	-4.3793	7.8193	0.9776	-4.2064	8.0283	0.7308
C	-2.9109	9.0242	2.3696	-2.789	9.0238	2.3238
C	-5.3865	8.3014	-0.1325	-5.3112	8.5663	-0.2494
C	-0.5627	8.5837	-0.7767	-0.2067	8.7603	-0.7487
C	-1.8859	5.3604	-2.8831	-2.8284	7.1482	-4.3827
C	0.4114	7.4699	-0.4035	0.6697	9.7389	-1.5264
C	-2.2411	9.872	3.4481	-2.144	9.6934	3.5344
C	-1.2024	9.0758	4.2584	-1.1606	8.7597	4.263
C	-3.2385	10.5751	4.3888	-3.1649	10.2823	4.527
O	-4.7779	9.3655	-0.8394	-5.3569	9.9829	-0.2414
O	-3.0732	7.7065	0.4248	-2.9356	8.2148	0.1532
C	-1.2084	9.2751	0.4373	-0.8002	9.2794	0.5866
C	-2.1154	8.3265	1.2776	-1.9815	8.4288	1.1793
C	-4.2039	8.7415	2.1639	-4.0801	8.7694	2.0485
O	-1.9696	10.4359	0.0673	-1.0317	10.703	0.5909
C	-1.4127	11.6558	0.0225	-1.9825	11.3262	-0.1398
C	-2.3929	12.7197	-0.4245	-1.9879	12.8224	0.0966
O	-0.2506	11.9362	0.3084	-2.7776	10.8111	-0.9225
C	-6.6907	8.8889	0.4446	-6.6998	8.0996	0.229
H	-2.259	8.4271	-4.4825	-0.8336	5.4099	-3.9343
H	-1.3033	7.0587	-4.9562	-1.1279	5.0026	-2.2777
H	0.2952	7.454	-3.1591	0.8963	6.3758	-2.4144
H	0.0584	8.9659	-3.9786	0.0465	7.6443	-3.2731
H	-1.5661	9.7284	-2.2255	-1.0104	6.8288	-0.5588
H	-6.3752	7.5964	-1.936	-4.2279	8.7378	-2.1353
H	-6.3947	6.4617	-0.6341	-5.9341	8.5581	-2.3148
H	-4.035	5.8263	-1.0519	-5.4987	6.0395	-1.4568
H	-5.0852	5.5699	-2.4074	-5.2903	6.5226	-3.1056
H	-4.0751	8.0922	-3.0548	-3.1567	5.677	-1.216
H	-4.6988	6.842	1.3435	-4.3585	6.9612	0.9053
H	-2.5647	4.7006	-2.3445	-2.7355	6.4817	-5.2404
H	-1.597	4.846	-3.7999	-3.8245	7.5871	-4.4142
H	-0.997	5.4893	-2.2658	-2.1169	7.9657	-4.5018
H	-0.136	6.5918	-0.0595	1.3491	10.2626	-0.8531
H	1.051	7.1531	-1.2239	1.2817	9.2457	-2.281
H	1.0738	7.7953	0.3994	0.0537	10.4784	-2.0379
H	-1.7029	10.6632	2.9225	-1.5683	10.5366	3.1487
H	-0.3985	8.7019	3.6237	-0.3459	8.4444	3.6108
H	-0.7388	9.6934	5.0286	-0.7074	9.2521	5.1243
H	-1.6585	8.218	4.7544	-1.6611	7.8601	4.6239
H	-3.917	11.2226	3.8318	-3.8091	11.0164	4.0411
H	-2.7246	11.2003	5.1199	-2.6691	10.7875	5.3567
H	-3.8446	9.8549	4.9398	-3.8056	9.5072	4.9493
H	-3.8389	9.1332	-0.8757	-4.4661	10.2801	-0.4979
H	-0.3942	9.5847	1.0963	0.0187	9.1681	1.2973
H	-1.5018	7.5407	1.7202	-1.5859	7.4642	1.5031
H	-5.0396	9.0771	2.7597	-4.9399	9.0194	2.6521
H	-3.2391	12.7528	0.2598	-1.0168	13.2423	-0.159
H	-1.9097	13.6952	-0.4377	-2.7503	13.2968	-0.5193
H	-2.7568	12.4912	-1.4247	-2.2022	13.0302	1.1434
H	-7.2061	8.165	1.076	-6.7771	7.013	0.251
H	-6.5073	9.7805	1.0447	-6.9188	8.4618	1.2338
H	-7.3793	9.1845	-0.3479	-7.4919	8.4709	-0.4222
	Conf.2 -**6**a			Conf.2 -**6**b		
C	3.61738	-0.80546	-0.62845	-4.5435	-7.8723	4.3876
C	3.49201	-2.28035	-0.28892	-3.4677	-6.8623	3.9975
C	2.55375	-2.68102	0.86092	-2.4851	-7.3475	2.9133
C	1.12739	-2.31798	0.5461	-3.1752	-7.608	1.5841
C	2.2656	2.55103	0.63865	-7.2002	-9.933	3.4882
C	3.32238	1.66736	-0.05156	-7.0005	-8.6322	4.3044
C	3.14963	0.17873	0.15575	-5.806	-7.7737	3.9146
C	0.14025	1.4438	-0.2653	-6.8304	-9.2594	0.9827
C	-2.10293	0.86964	0.0833	-6.2799	-9.83	-1.2287
C	0.90796	2.7621	-0.06887	-7.8168	-9.8335	2.0607
C	0.18602	-1.88604	1.39266	-3.1083	-8.707	0.7973
C	4.36944	-0.56164	-1.91398	-4.0739	-8.9555	5.35
C	0.36829	-1.74506	2.88155	-2.1946	-9.8965	1.075
C	-3.59606	0.69083	0.02316	-6.204	-10.0776	-2.7321
C	-4.18326	1.10201	-1.3295	-7.476	-9.6513	-3.4895
C	-4.26992	1.45746	1.17721	-5.8337	-11.5324	-3.0713
O	0.11622	3.62916	0.76779	-8.1994	-11.154	1.7149
O	0.02344	0.81102	1.02551	-5.6323	-10.0216	0.9919
C	-1.13721	-1.36363	0.87729	-3.9821	-8.8676	-0.4607
C	-1.25357	0.17198	1.12565	-5.0705	-9.983	-0.3164
C	-1.29281	1.56991	-0.71486	-7.2914	-9.3986	-0.4566
O	-1.23816	-1.59941	-0.54749	-4.5826	-7.6247	-0.8692
C	-2.20038	-2.41379	-1.01415	-3.9606	-6.791	-1.7207
C	-2.11796	-2.53536	-2.50894	-4.7664	-5.5383	-1.9942
O	-3.01671	-2.98233	-0.30793	-2.8687	-6.9768	-2.2535
C	1.07088	3.48605	-1.40155	-9.1352	-9.0321	2.0827
H	4.50034	-2.6695	-0.07948	-2.9158	-6.5802	4.8956
H	3.1674	-2.81178	-1.19677	-3.9435	-5.9395	3.6596
H	2.65168	-3.76844	0.99779	-1.7288	-6.5793	2.7471
H	2.88332	-2.22313	1.7999	-1.9537	-8.2245	3.2772
H	0.87293	-2.36732	-0.5103	-3.8575	-6.8171	1.3058
H	2.0684	2.15755	1.64332	-6.2494	-10.4662	3.4389
H	2.68417	3.55513	0.77614	-7.8436	-10.5791	4.0892
H	4.29736	1.96058	0.36754	-6.8546	-8.9152	5.3472
H	3.37943	1.91642	-1.11638	-7.9038	-8.0226	4.3284
H	2.63334	-0.09516	1.07273	-6.0362	-6.9693	3.2293
H	0.71803	0.7941	-0.93995	-6.6061	-8.2149	1.2055
H	5.3764	-0.99992	-1.8611	-3.2016	-9.4688	4.945
H	3.86418	-1.05188	-2.75808	-3.8001	-8.5166	6.3096
H	4.48223	0.49777	-2.15582	-4.8271	-9.7187	5.5385
H	-0.52754	-2.08338	3.41821	-1.6552	-10.1814	0.1712

H	0.53434	-0.69736	3.16526	-2.7838	-10.7518	1.4072
H	1.21656	-2.32974	3.24528	-1.4445	-9.7001	1.8381
H	-3.81029	-0.37647	0.17451	-5.3935	-9.4435	-3.0983
H	-3.72677	0.53487	-2.1489	-7.6916	-8.5933	-3.3346
H	-5.26358	0.91889	-1.34821	-7.3711	-9.8048	-4.5641
H	-4.02195	2.16939	-1.52342	-8.3469	-10.2191	-3.1593
H	-3.87738	1.14564	2.1516	-4.8529	-11.8006	-2.6781
H	-4.10032	2.53596	1.07285	-6.5594	-12.2332	-2.6562
H	-5.35151	1.27839	1.17582	-5.7991	-11.6944	-4.1493
H	-0.14223	3.08619	1.53456	-7.3843	-11.6101	1.473
H	-1.98218	-1.85636	1.36394	-3.3235	-9.2004	-1.2664
H	-1.6099	0.30978	2.15189	-4.5881	-10.9494	-0.4671
H	-1.5848	2.12977	-1.59474	-8.2931	-9.1494	-0.7727
H	-3.0259	-3.00192	-2.89419	-5.7419	-5.8062	-2.3961
H	-1.25361	-3.15816	-2.76933	-4.2478	-4.9091	-2.7157
H	-1.96943	-1.55269	-2.96552	-4.9033	-4.9777	-1.0714
H	0.09559	3.76403	-1.81288	-9.6803	-9.1212	1.1428
H	1.65651	4.40155	-1.26282	-9.8024	-9.3858	2.8696
H	1.58561	2.85637	-2.13359	-8.9575	-7.9701	2.2506
	Conf.2 -**6**c			Conf.2 -**6**d		
C	-5.3783	-7.642	4.1105	-4.2584	-8.2648	4.3067
C	-4.7785	-6.3823	3.4896	-3.3149	-7.0841	4.0902
C	-3.5435	-6.6074	2.5861	-2.3561	-7.2545	2.8953
C	-3.8284	-7.3404	1.2794	-3.1084	-7.4917	1.5946
C	-8.1603	-9.9909	3.2496	-7.9398	-9.0333	3.3549
C	-7.3808	-9.2459	4.3659	-6.5842	-9.3168	4.0515
C	-6.6708	-7.977	3.9065	-5.543	-8.2184	3.8941
C	-6.3193	-9.8516	1.3991	-7.1133	-9.0386	0.8577
C	-6.3391	-8.5879	-0.5726	-6.2746	-9.8644	-1.1813
C	-7.3433	-10.7561	2.1628	-8.1689	-9.5558	1.9056
C	-3.402	-8.5645	0.8861	-3.1159	-8.6012	0.8201
C	-4.4305	-8.439	4.9969	-3.6431	-9.454	5.0306
C	-2.5268	-9.4855	1.7295	-2.2279	-9.8175	1.0681
C	-6.5886	-7.5853	-1.6979	-6.0182	-10.2996	-2.6215
C	-7.6302	-6.5071	-1.3407	-7.2327	-10.1058	-3.5499
C	-6.9603	-8.2705	-3.0258	-5.4891	-11.7419	-2.7177
O	-6.585	-11.744	2.8365	-8.0644	-10.9673	1.9557
O	-5.602	-10.5766	0.4061	-5.8444	-9.6335	1.1057
C	-3.775	-9.1515	-0.4834	-4.0677	-8.7592	-0.3783
C	-5.2547	-9.6605	-0.6321	-5.1719	-9.833	-0.1353
C	-6.9467	-8.7214	0.6149	-7.389	-9.4108	-0.587
O	-3.4025	-8.2155	-1.5025	-4.6738	-7.5164	-0.7737
C	-3.1797	-8.6059	-2.7686	-4.0931	-6.7121	-1.6795
C	-2.7887	-7.4537	-3.6695	-4.8884	-5.4462	-1.9209
O	-3.2777	-9.7521	-3.2015	-3.0438	-6.9316	-2.2807
C	-8.2852	-11.5353	1.2247	-9.6284	-9.2694	1.4938
H	-4.4999	-5.7174	4.3086	-2.7479	-6.9067	5.0053
H	-5.5404	-5.8408	2.9256	-3.9042	-6.1771	3.9413
H	-3.1354	-5.6343	2.3097	-1.7489	-6.3562	2.7775
H	-2.7498	-7.0837	3.1571	-1.6564	-8.0565	3.1177
H	-4.4474	-6.7801	0.5911	-3.7833	-6.6843	1.3471
H	-8.8317	-10.7029	3.7326	-8.7177	-9.4696	3.9844
H	-8.8296	-9.2831	2.7599	-8.1444	-7.9622	3.3867
H	-6.7176	-9.9454	4.873	-6.1709	-10.2777	3.7427
H	-8.0986	-8.9213	5.1204	-6.7616	-9.3865	5.1261
H	-7.2775	-7.3243	3.2938	-5.8829	-7.3266	3.3854
H	-5.5947	-9.4667	2.1126	-7.0335	-7.9528	0.9396
H	-3.7699	-7.774	5.554	-2.8367	-9.8802	4.4333
H	-4.9516	-9.0537	5.729	-3.2351	-9.1449	5.9932
H	-3.8144	-9.0995	4.3889	-4.3618	-10.2498	5.2225
H	-2.2296	-9.0652	2.6861	-1.7863	-10.1648	0.1335
H	-3.0589	-10.4145	1.9382	-2.8169	-10.6313	1.492
H	-1.6097	-9.729	1.1922	-1.4022	-9.6266	1.7493
H	-5.6485	-7.0551	-1.8538	-5.231	-9.6412	-2.9956
H	-7.3306	-5.9471	-0.4539	-7.5514	-9.0629	-3.5708
H	-7.7524	-5.7866	-2.1502	-7.0004	-10.3919	-4.5764
H	-8.6085	-6.9464	-1.141	-8.0834	-10.7064	-3.2252
H	-6.1647	-8.9266	-3.3793	-4.5334	-11.8554	-2.2056
H	-7.8623	-8.8748	-2.9207	-6.189	-12.4513	-2.274
H	-7.1431	-7.5398	-3.8145	-5.3306	-12.0405	-3.7547
H	-5.9912	-12.1199	2.1752	-7.1158	-11.1483	1.9262
H	-3.1355	-10.0282	-0.6093	-3.4683	-9.1279	-1.2139
H	-5.3336	-10.2177	-1.5659	-4.6904	-10.8093	-0.0714
H	-7.7481	-8.114	1.0067	-8.3546	-9.2726	-1.049
H	-3.5988	-6.7281	-3.7146	-5.8842	-5.6981	-2.2811
H	-2.5847	-7.8169	-4.6755	-4.3901	-4.8269	-2.665
H	-1.8955	-6.9665	-3.2825	-4.9769	-4.8819	-0.9944
H	-7.7302	-12.1234	0.493	-9.8979	-9.766	0.5618
H	-8.926	-12.2237	1.7772	-10.3345	-9.6241	2.2456
H	-8.9339	-10.8581	0.6692	-9.7982	-8.2009	1.3614
	Con f. 1- **7**i			Con f. 1- **7**j		
C	0.56257	2.14566	-0.67639	1.23172	1.50909	0.76314
C	-0.25917	2.9912	-1.62258	0.53869	2.83232	0.50144
C	-1.75112	3.10382	-1.22821	0.09664	3.1358	-0.94327
C	-2.423	1.77105	-1.02152	-0.87494	2.11925	-1.49712
C	2.52191	-1.08659	-1.06467	1.82722	-1.63687	-1.017
C	2.15397	0.20943	-0.33271	2.44879	-0.55906	-0.12068
C	1.34358	1.16542	-1.15927	1.76471	0.77151	-0.22372
C	0.47849	-2.48813	-0.42605	-0.26947	-1.75143	0.55523
C	-3.84193	0.02093	0.1038	-2.9887	0.8368	-1.74301
C	-3.09399	-1.17051	-0.5089	-2.48901	-0.52748	-1.23356
C	-1.69667	-1.40517	0.09572	-2.54018	-0.68958	0.29369
C	-1.01635	-2.6455	-0.5385	-1.7541	-1.9454	0.7473
C	1.29593	-1.88404	-1.49085	0.54872	-2.30746	-0.53816
C	-3.17209	1.38702	0.02492	-2.17673	2.00335	-1.19457
C	0.46133	2.49759	0.7831	1.23289	1.12515	2.22105
C	-3.47829	2.25929	1.21695	-2.91608	2.94335	-0.27971

C	-1.7161	-1.49234	1.61361	-3.94006	-0.71995	0.89084
C	-2.60453	-2.53119	2.25007	-4.0481	-0.16575	2.28815
O	1.36706	-3.3178	-1.21137	0.61438	-2.876	0.80219
O	3.41267	0.88645	0.02802	3.8093	-0.426	-0.67651
C	3.99742	0.72357	1.22929	4.84689	0.06473	0.02609
O	5.05186	1.30945	1.42388	5.91236	0.18352	-0.5601
H	0.83491	-2.35185	0.59646	0.08983	-0.83615	1.01499
C	0.6979	-1.56132	-2.84559	-0.04397	-3.23205	-1.57811
C	3.33661	-0.14011	2.27205	4.65769	0.42755	1.47199
C	-0.96361	-0.67059	2.35641	-5.00272	-1.22691	0.25624
H	0.15476	4.00996	-1.65448	-0.34777	2.87492	1.14833
H	-0.17645	2.58176	-2.63614	1.19638	3.63969	0.85968
H	-2.25722	3.65049	-2.03796	0.97578	3.19608	-1.59546
H	-1.84252	3.73012	-0.33703	-0.34877	4.1382	-0.94154
H	-2.27371	1.05902	-1.8347	-0.45063	1.37415	-2.1695
H	3.11887	-1.70674	-0.3884	2.57583	-2.42755	-1.15236
H	3.14405	-0.84197	-1.9334	1.6422	-1.20598	-2.00841
H	1.62375	-0.03034	0.58719	2.52562	-0.92786	0.9018
H	1.40614	1.03682	-2.23692	1.70579	1.13531	-1.24685
H	-4.8371	0.0905	-0.36133	-2.94379	0.83034	-2.84007
H	-4.0358	-0.19674	1.16131	-4.04369	0.96327	-1.47539
H	-2.97663	-1.0343	-1.59094	-1.45055	-0.6502	-1.55694
H	-3.71152	-2.06897	-0.38107	-3.0556	-1.334	-1.71539
H	-1.08677	-0.53453	-0.16811	-2.02504	0.17694	0.73167
H	-1.31141	-2.72882	-1.58792	-2.12362	-2.82747	0.2155
H	-1.34084	-3.5679	-0.04303	-1.92746	-2.12126	1.81674
H	0.68237	3.56469	0.92171	1.80367	0.22142	2.44165
H	-0.55467	2.33525	1.15823	1.64313	1.94401	2.82701
H	1.15282	1.93067	1.4096	0.20275	0.96861	2.56906
H	-3.08676	1.79634	2.13327	-2.30501	3.78342	0.06116
H	-3.06941	3.26916	1.14315	-3.80409	3.35045	-0.78344
H	-4.56529	2.34797	1.3559	-3.28512	2.41023	0.60742
H	-2.51469	-2.51346	3.34081	-3.32305	-0.64213	2.96175
H	-3.65889	-2.36694	1.99428	-3.81796	0.90876	2.29627
H	-2.35507	-3.5431	1.9067	-5.04929	-0.30547	2.70854
H	0.08342	-0.65703	-2.80731	-0.89249	-3.79485	-1.18413
H	1.49325	-1.39819	-3.58139	-0.3781	-2.66214	-2.45194
H	0.07423	-2.38519	-3.19893	0.71511	-3.94863	-1.91429
H	3.99221	-0.18435	3.14278	4.30037	-0.43067	2.0502
H	2.3715	0.28211	2.57294	5.61474	0.76281	1.87371
H	3.15723	-1.15366	1.90057	3.91917	1.22845	1.56819
H	-0.32588	0.08179	1.90217	-4.94614	-1.62414	-0.75364
H	-0.95864	-0.72419	3.44357	-5.97992	-1.2625	0.73347
		Con f. 2- 7a			Con f. 2- 7b	
C	1.28073	2.00385	0.16428	1.16775	1.59041	0.13212
C	0.30343	2.86777	0.93547	0.54349	2.587	1.08904
C	-0.81353	3.54122	0.10233	-0.7018	3.35646	0.58819
C	-1.69775	2.57435	-0.63655	-1.9376	2.50493	0.53668
C	2.1287	-1.61755	0.06182	1.91614	-2.05859	-0.14975
C	2.60936	-0.16172	0.05211	2.41489	-0.61338	-0.14825
C	1.6163	0.79337	0.64047	1.55678	0.38183	0.57683
C	-0.42774	-1.61493	-0.07721	-0.60477	-2.5731	-0.11923
C	-3.6749	1.1802	-1.18935	-3.85865	1.21458	-0.34819
C	-4.01792	-0.15488	-0.49255	-3.55083	-0.22935	-0.79626
C	-2.81697	-0.82521	0.20505	-2.43068	-0.88324	0.029
C	-1.66533	-1.10014	-0.77777	-2.00442	-2.2455	-0.56916
C	0.86261	-1.79097	-0.75425	0.60837	-2.25941	-0.89486
C	-2.93648	2.16838	-0.31056	-2.67781	2.14877	-0.52528
C	1.86103	2.62312	-1.08003	1.39963	2.09908	-1.26605
C	-3.68543	2.62136	0.91688	-2.40124	2.57252	-1.94534
C	-3.23882	-2.07496	0.95893	-2.78346	-1.0251	1.50241
C	-3.57348	-3.30422	0.15204	-4.11134	-1.65132	1.8455
O	0.06086	-2.95032	-0.38364	0.11872	-3.63038	-0.79699
O	3.81322	-0.08769	0.89981	3.70097	-0.69466	0.57254
C	4.99895	-0.33115	0.31678	4.62609	0.24348	0.31336
O	5.1307	-0.60777	-0.86564	4.4902	1.12339	-0.5218
H	-0.38901	-1.39865	0.99062	-0.48254	-2.60916	0.96386
C	0.99004	-1.54221	-2.23924	0.54087	-1.65804	-2.28039
C	6.12595	-0.2177	1.30557	5.83428	0.07155	1.19211
C	-3.30554	-2.07561	2.29544	-1.93506	-0.6254	2.45759
H	-0.15447	2.27598	1.7363	0.30957	2.09246	2.03933
H	0.87724	3.67301	1.41993	1.3205	3.33545	1.30954
H	-1.39685	4.1684	0.78428	-0.87046	4.17978	1.29722
H	-0.3505	4.22428	-0.6218	-0.48795	3.82278	-0.37747
H	-1.26032	2.15638	-1.54444	-2.24643	2.11438	1.50733
H	1.93531	-1.91999	1.09682	1.81071	-2.41079	0.88218
H	2.92316	-2.25841	-0.33813	2.6863	-2.67373	-0.63269
H	2.91076	0.12654	-0.95282	2.63752	-0.29459	-1.16446
H	1.1453	0.4519	1.5617	1.33615	0.10881	1.60899
H	-3.0879	0.98623	-2.09368	-4.71396	1.58461	-0.93047
H	-4.62151	1.63066	-1.5232	-4.16812	1.20445	0.70338
H	-4.44581	-0.83391	-1.24078	-3.24695	-0.22911	-1.85015
H	-4.79937	0.01257	0.2585	-4.4668	-0.83041	-0.73962
H	-2.43703	-0.11168	0.94742	-1.55774	-0.22374	-0.03576
H	-1.40254	-0.1647	-1.28008	-2.05222	-2.19932	-1.6607
H	-1.97692	-1.80263	-1.55769	-2.68867	-3.04348	-0.25459
H	2.2151	3.63994	-0.86519	2.04472	1.4523	-1.8617
H	2.69786	2.05638	-1.49337	0.44614	2.20035	-1.7972
H	1.09886	2.71729	-1.86392	1.85199	3.09894	-1.23798
H	-4.68492	2.98947	0.64715	-1.59161	3.30158	-2.02573
H	-3.83974	1.78446	1.61098	-2.13572	1.70906	-2.57028
H	-3.16916	3.41263	1.46607	-3.30047	3.01517	-2.39562
H	-3.97105	-4.10116	0.78856	-4.19517	-1.83971	2.92054
H	-4.31368	-3.08762	-0.62854	-4.94509	-1.00033	1.552
H	-2.68304	-3.69293	-0.35894	-4.25879	-2.60306	1.31913
H	1.16554	-0.47802	-2.4354	0.46673	-0.56642	-2.22712
H	1.83575	-2.10471	-2.65189	1.44712	-1.9073	-2.84543

H	0.08266	-1.84139	-2.76902	-0.31937	-2.0357	-2.83677
H	5.9985	-0.96525	2.09637	6.17284	-0.96893	1.1786
H	6.11133	0.76995	1.7783	6.63436	0.73322	0.85641
H	7.07998	-0.37538	0.80027	5.56528	0.3203	2.22548
H	-3.04709	-1.1939	2.87915	-0.9809	-0.1648	2.21474
H	-3.62388	-2.9538	2.85422	-2.16378	-0.74891	3.51464
	Con f. 2- **7c**			Con f. 2- **7d**		
C	1.49216	1.6785	0.01436	-1.53607	1.6272	-0.00601
C	0.64106	2.62853	0.83004	-0.7116	2.60074	-0.82561
C	-0.40862	3.42861	0.02067	0.34914	3.41636	-0.04954
C	-1.38553	2.54494	-0.70277	1.37634	2.57662	0.6579
C	1.84023	-1.98782	-0.45712	-1.9295	-2.04324	0.19605
C	2.5186	-0.6344	-0.2333	-2.6217	-0.67998	0.12481
C	1.64974	0.40371	0.41063	-1.76594	0.39181	-0.48124
C	-0.72215	-2.18225	-0.50885	0.6172	-1.77145	0.2683
C	-3.47295	1.28383	-1.15932	3.53303	1.45832	1.14897
C	-3.88388	0.0406	-0.34274	3.9961	0.16228	0.44849
C	-2.66787	-0.74512	0.19625	2.85031	-0.66363	-0.17581
C	-2.08855	-1.67215	-0.89467	1.82772	-1.09795	0.88244
C	0.53604	-1.90675	-1.23231	-0.64138	-1.99564	0.99395
C	-2.65224	2.26762	-0.35426	2.64651	2.33657	0.2915
C	2.1377	2.28735	-1.20328	-2.0933	2.16875	1.28413
C	-3.3359	2.82973	0.86753	3.29005	2.87168	-0.9622
C	-3.0071	-1.48683	1.47719	3.41255	-1.80682	-1.00709
C	-4.0606	-2.56274	1.4103	3.92651	-1.42007	-2.37125
O	-0.1176	-3.20235	-1.34443	0.25708	-3.10653	0.71326
O	3.63001	-0.95573	0.68268	-3.78101	-0.91635	-0.75471
C	4.70888	-0.15699	0.67715	-4.84264	-0.10407	-0.62593
O	4.85476	0.78982	-0.07928	-4.92141	0.78794	0.20366
H	-0.61922	-2.40619	0.55435	0.5348	-1.66045	-0.8133
C	0.56708	-1.1054	-2.51279	-0.76793	-1.62226	2.45193
C	5.6992	-0.57678	1.72839	-5.90527	-0.43273	-1.63787
C	-2.40183	-1.17026	2.6287	3.46274	-3.07439	-0.58079
H	0.13339	2.07755	1.62985	-0.22161	2.06447	-1.6463
H	1.31211	3.35555	1.31289	-1.40494	3.3232	-1.2839
H	-0.9206	4.10137	0.71536	0.82234	4.10025	-0.761
H	0.11253	4.06849	-0.70301	-0.16233	4.04778	0.68882
H	-0.99063	2.04728	-1.58978	1.03031	2.10899	1.58049
H	1.65401	-2.45767	0.5146	-1.70416	-2.37982	-0.82183
H	2.53772	-2.63221	-1.00713	-2.62045	-2.7679	0.64437
H	2.95642	-0.27637	-1.16312	-3.00939	-0.39517	1.10125
H	1.13411	0.06714	1.31034	-1.3135	0.11054	-1.43227
H	-2.91044	0.97549	-2.04872	3.01084	1.21333	2.08013
H	-4.39121	1.77343	-1.51565	4.4343	2.02057	1.43511
H	-4.50678	-0.6111	-0.96825	4.53224	-0.46243	1.17479
H	-4.51307	0.35886	0.49638	4.71954	0.417	-0.33397
H	-1.89046	-0.01446	0.44813	2.32021	0.00427	-0.87064
H	-2.04566	-1.13318	-1.84517	1.49114	-0.20962	1.4245
H	-2.75705	-2.52814	-1.04983	2.28706	-1.75975	1.62502
H	2.61667	3.23911	-0.93849	-2.57219	3.14135	1.10962
H	2.89238	1.64827	-1.66107	-2.83002	1.51051	1.7454
H	1.38368	2.51887	-1.96599	-1.29127	2.34182	2.01251
H	-4.37048	3.11652	0.63767	2.65494	3.57686	-1.50415
H	-3.38897	2.07704	1.6662	4.23729	3.3761	-0.7267
H	-2.8251	3.70451	1.27788	3.53785	2.05407	-1.65245
H	-4.27087	-2.97593	2.40189	4.36271	-2.27465	-2.89855
H	-5.00006	-2.17603	0.99469	3.11217	-1.01459	-2.98784
H	-3.74886	-3.39031	0.76018	4.6898	-0.63354	-2.30934
H	0.64115	-0.03395	-2.3002	-1.07479	-0.57495	2.55611
H	1.43635	-1.39294	-3.11634	-1.52529	-2.24538	2.94231
H	-0.3315	-1.2795	-3.10888	0.18129	-1.754	2.97703
H	5.27421	-0.38574	2.72076	-5.53953	-0.1821	-2.64047
H	6.62446	-0.00995	1.61378	-6.80864	0.14136	-1.42602
H	5.90189	-1.64979	1.65546	-6.12671	-1.50444	-1.62393
H	-1.6404	-0.394	2.67747	3.07886	-3.38374	0.38682
H	-2.64789	-1.67143	3.56304	3.8927	-3.85907	-1.20015
	Con f. 2- 7e			Con f. 2- 7f		
C	1.17793	1.73936	0.81062	1.15787	1.62534	0.08035
C	0.2239	2.49652	1.7051	0.52318	2.63575	1.01593
C	-0.85693	3.30277	0.93791	-0.71786	3.40052	0.49089
C	-1.86864	2.39269	0.3022	-1.97099	2.57235	0.50724
C	1.74081	-1.82586	-0.30013	1.84435	-2.04278	-0.19886
C	2.2242	-0.41665	0.04524	2.36274	-0.60335	-0.17403
C	1.27913	0.40052	0.87441	1.50646	0.40703	0.5334
C	-0.78094	-2.32994	-0.41412	-0.66497	-2.61508	-0.17244
C	-3.09549	1.12512	-1.48637	-3.88486	1.20424	-0.27301
C	-3.67794	0.15966	-0.44553	-3.64064	-0.25949	-0.69254
C	-2.61821	-0.76651	0.19349	-2.48046	-0.91689	0.07409
C	-2.17856	-1.8952	-0.77029	-2.07386	-2.26451	-0.57203
C	0.43882	-1.8416	-1.08359	0.52906	-2.21568	-0.93948
C	-2.08023	2.14649	-0.99969	-2.68623	2.09614	-0.52405
C	2.00253	2.59596	-0.11986	1.4514	2.13605	-1.30624
C	-1.34867	2.83635	-2.12356	-2.35432	2.33961	-1.97474
C	-3.07396	-1.30486	1.53824	-2.76077	-1.07921	1.56939
C	-4.36167	-2.08738	1.58291	-1.69899	-0.59021	2.51996
O	-0.05698	-3.19188	-1.32614	0.0654	-3.59946	-0.94474
O	3.46501	-0.57593	0.82963	3.63007	-0.71152	0.57555
C	4.63505	-0.45651	0.1809	4.58976	0.19329	0.32398
O	4.73277	-0.23121	-1.0161	4.49893	1.06758	-0.52315
H	-0.66467	-2.6325	0.62801	-0.5191	-2.74667	0.89991
C	0.38444	-0.91952	-2.28055	0.42894	-1.51662	-2.27676
C	5.79701	-0.63346	1.11878	5.77514	-0.00839	1.22716
C	-2.3621	-1.06416	2.6466	-3.88444	-1.64861	2.02029
H	-0.27	1.805	2.39808	0.27918	2.15694	1.97155
H	0.80706	3.20298	2.31339	1.29911	3.38656	1.23169
H	-1.36093	3.95127	1.66786	-0.86124	4.2648	1.15433
H	-0.3776	3.9639	0.21036	-0.51138	3.80737	-0.50306

H	-2.45317	1.83314	1.03083	-2.31876	2.31683	1.50923
H	1.62614	-2.4079	0.62081	1.74352	-2.41403	0.82681
H	2.51906	-2.30761	-0.9057	2.60522	-2.65582	-0.69881
H	2.4983	0.10672	-0.86709	2.61302	-0.28448	-1.18338
H	0.66499	-0.14598	1.58857	1.24761	0.13519	1.5565
H	-2.6253	0.54883	-2.29615	-4.75127	1.58319	-0.8338
H	-3.92377	1.66258	-1.97353	-4.15188	1.23007	0.78996
H	-4.4564	-0.44547	-0.9269	-3.41469	-0.29768	-1.76512
H	-4.18109	0.73359	0.34238	-4.5639	-0.8341	-0.55139
H	-1.73047	-0.15796	0.3827	-1.61678	-0.24874	-0.02746
H	-2.213	-1.54393	-1.80426	-2.14454	-2.1876	-1.66083
H	-2.85988	-2.75221	-0.70611	-2.76181	-3.05867	-0.25757
H	1.41176	2.91768	-0.98672	2.11631	1.4875	-1.87737
H	2.3252	3.50864	0.39616	0.52379	2.24868	-1.87883
H	2.89563	2.09056	-0.4941	1.91093	3.13164	-1.25379
H	-2.06259	3.24873	-2.85042	-1.60807	3.12541	-2.11395
H	-0.70734	3.65329	-1.78517	-1.96932	1.42867	-2.4526
H	-0.72434	2.12076	-2.67622	-3.25586	2.62603	-2.53294
H	-4.5819	-2.43454	2.59742	-1.97808	-0.76589	3.56435
H	-5.20738	-1.47639	1.2416	-0.73867	-1.08732	2.33462
H	-4.32627	-2.96485	0.92455	-1.51798	0.48205	2.3851
H	-0.48495	-1.12914	-2.90651	0.3594	-0.43194	-2.14593
H	0.33768	0.12664	-1.95998	1.32155	-1.72692	-2.87824
H	1.28429	-1.04607	-2.89441	-0.44385	-1.85346	-2.83934
H	5.75879	-1.63077	1.57033	6.08348	-1.05836	1.2249
H	5.73517	0.09983	1.92992	6.60048	0.62781	0.90363
H	6.73493	-0.50934	0.57545	5.49388	0.25332	2.254
H	-1.43914	-0.48857	2.61825	-4.66096	-2.01157	1.35081
H	-2.67806	-1.43155	3.62132	-4.06746	-1.77865	3.0849
		Con f. 2- 7g				
C	-1.32641	2.02022	0.04576			
C	-0.31702	2.77847	-0.78322			
C	0.80122	3.45529	0.05233			
C	1.66137	2.44759	0.7629			
C	-2.10625	-1.60298	0.83025			
C	-2.47591	-0.12478	0.68138			
C	-1.50612	0.69908	-0.1196			
C	0.36509	-2.26906	0.59687			
C	3.54584	0.86823	1.12632			
C	3.72146	-0.39633	0.25796			
C	2.36277	-0.97907	-0.19978			
C	1.82687	-1.99146	0.83519			
C	-0.73667	-1.79974	1.45868			
C	2.86274	1.98988	0.37447			
C	-2.09615	2.85088	1.04366			
C	3.58512	2.46701	-0.86223			
C	2.43697	-1.55063	-1.60402			
C	3.32166	-2.74976	-1.82769			
O	-0.3188	-3.19481	1.47957			
O	-3.86481	-0.01875	0.18252			
C	-4.13229	-0.31212	-1.09889			
O	-3.29964	-0.69268	-1.90664			
H	0.1119	-2.43495	-0.45162			
C	-0.47992	-1.04888	2.74533			
C	-5.59019	-0.10031	-1.40696			
C	1.75532	-0.98126	-2.60618			
H	0.14589	2.10837	-1.51661			
H	-0.84407	3.56456	-1.34377			
H	1.39381	4.07818	-0.62489			
H	0.34123	4.13542	0.78059			
H	1.21792	2.00305	1.65504			
H	-2.13056	-2.09799	-0.14334			
H	-2.86099	-2.07707	1.47012			
H	-2.58856	0.30424	1.67632			
H	-0.92176	0.17514	-0.87127			
H	2.97014	0.61846	2.02632			
H	4.54123	1.19587	1.46017			
H	4.28597	-1.14844	0.82298			
H	4.3319	-0.14455	-0.61629			
H	1.64885	-0.1487	-0.23788			
H	1.98617	-1.59599	1.84238			
H	2.39039	-2.93083	0.77319			
H	-2.37609	3.81384	0.59878			
H	-3.01168	2.36641	1.3892			
H	-1.48058	3.07926	1.92405			
H	3.23536	3.44193	-1.21158			
H	4.66472	2.54083	-0.67804			
H	3.45478	1.75675	-1.6905			
H	3.38572	-3.0066	-2.88984			
H	4.33856	-2.569	-1.4558			
H	2.94485	-3.63002	-1.29094			
H	-0.3665	0.02444	2.55621			
H	-1.32038	-1.1849	3.43646			
H	0.42506	-1.40961	3.23861			
H	-5.8479	0.95442	-1.25944			
H	-6.20839	-0.69093	-0.72276			
H	-5.79612	-0.38916	-2.4387			
H	1.11285	-0.11865	-2.43937			
H	1.81524	-1.35543	-3.62651			

4. The 1D and 2D NMR spectra of 1–7

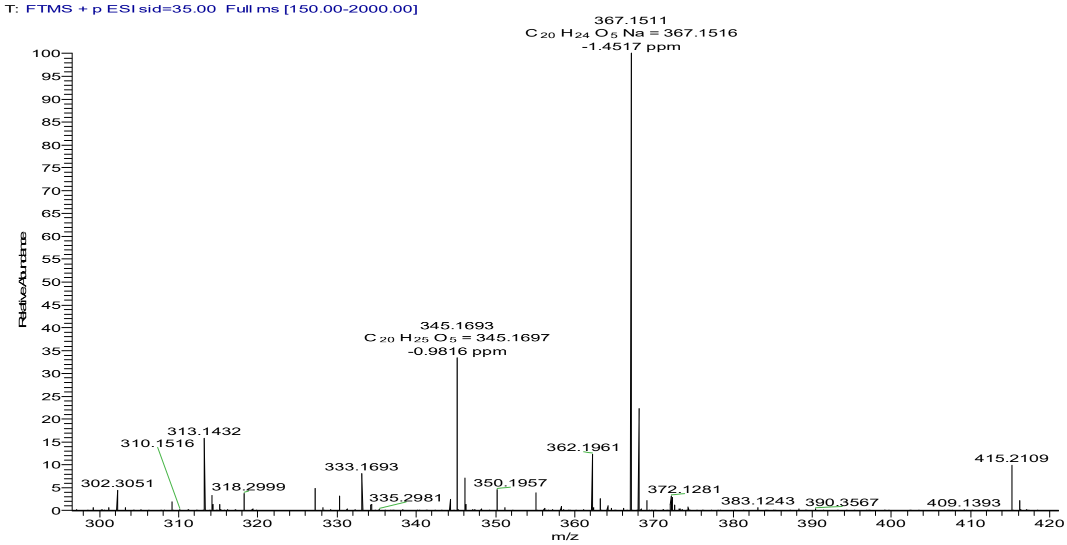

Figure S18. The positive HRESIMS spectrum of **1**

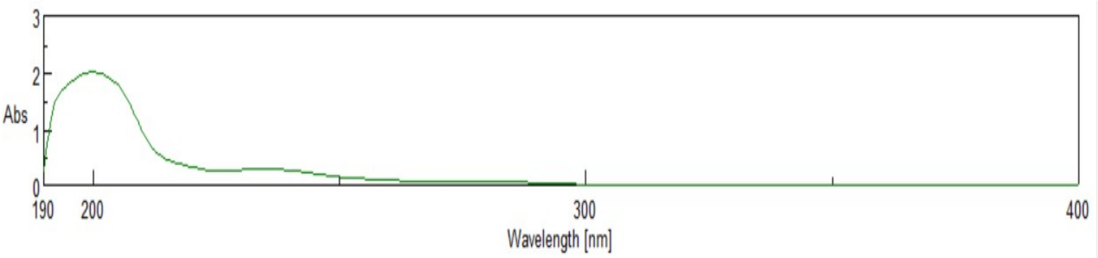

Figure S19. UV spectrum of compound **1**

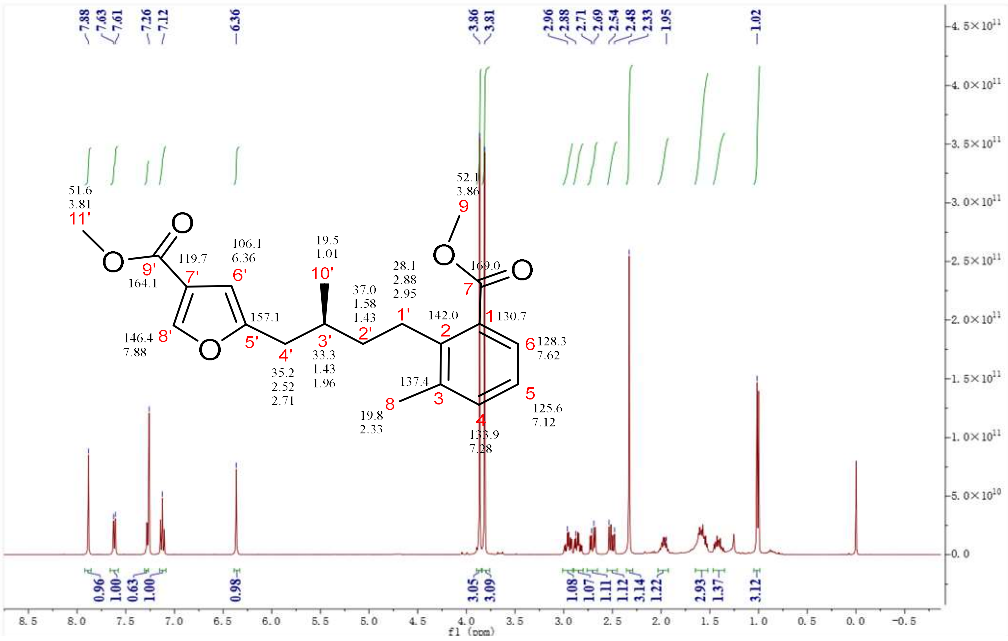

Figure S20. ^{1}H NMR spectrum (400 MHz, $CDCl_3$) of **1**

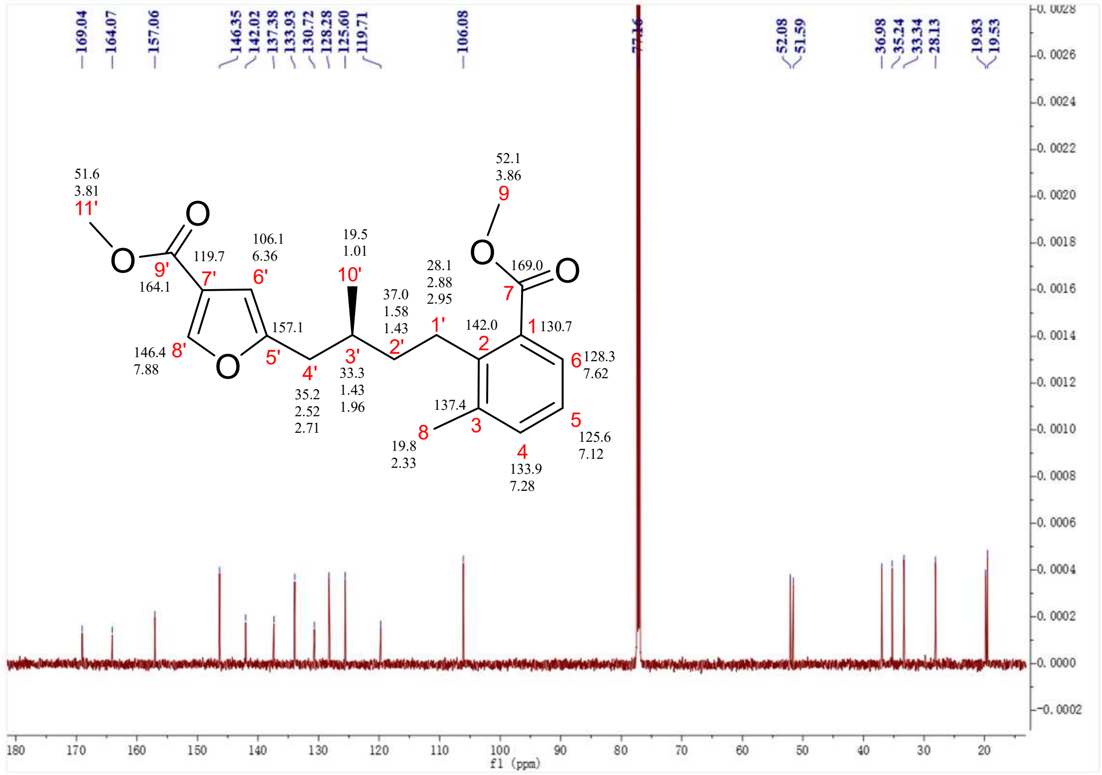

Figure S21. ^{13}C NMR spectrum 125 MHz, $CDCl_3$) of **1**

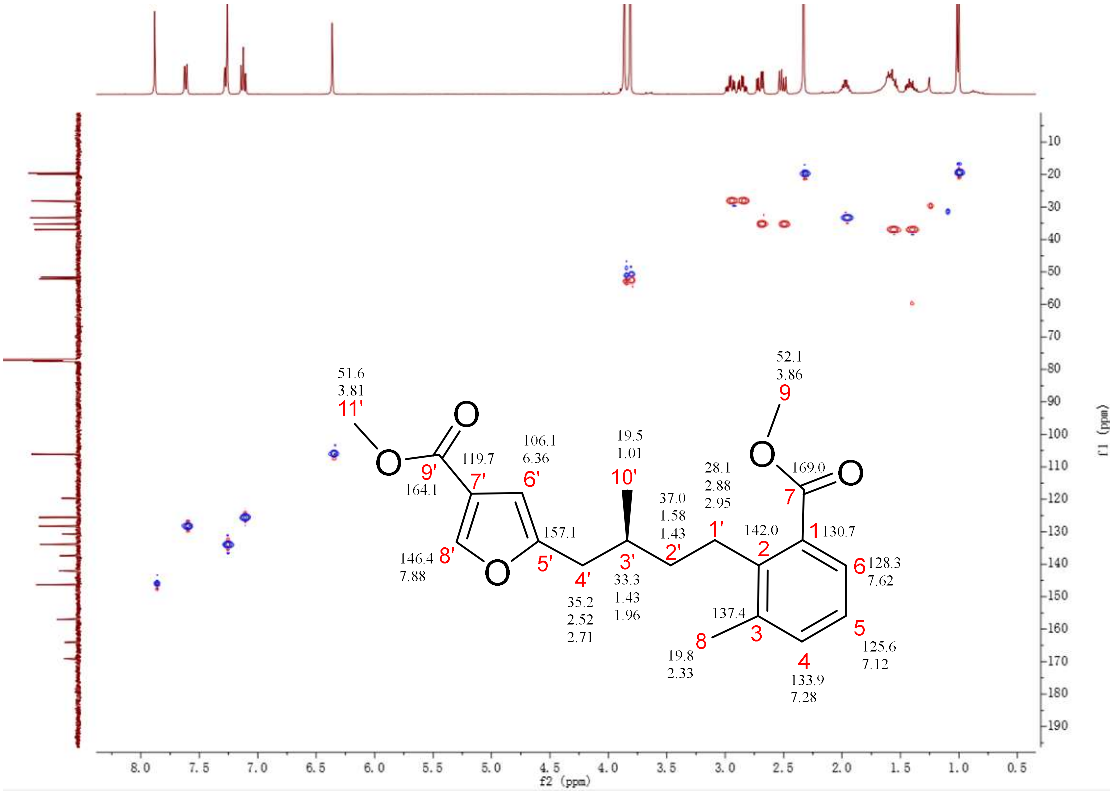

Figure S22. HSQC (500 M Hz, $CDCl_3$) of **1**

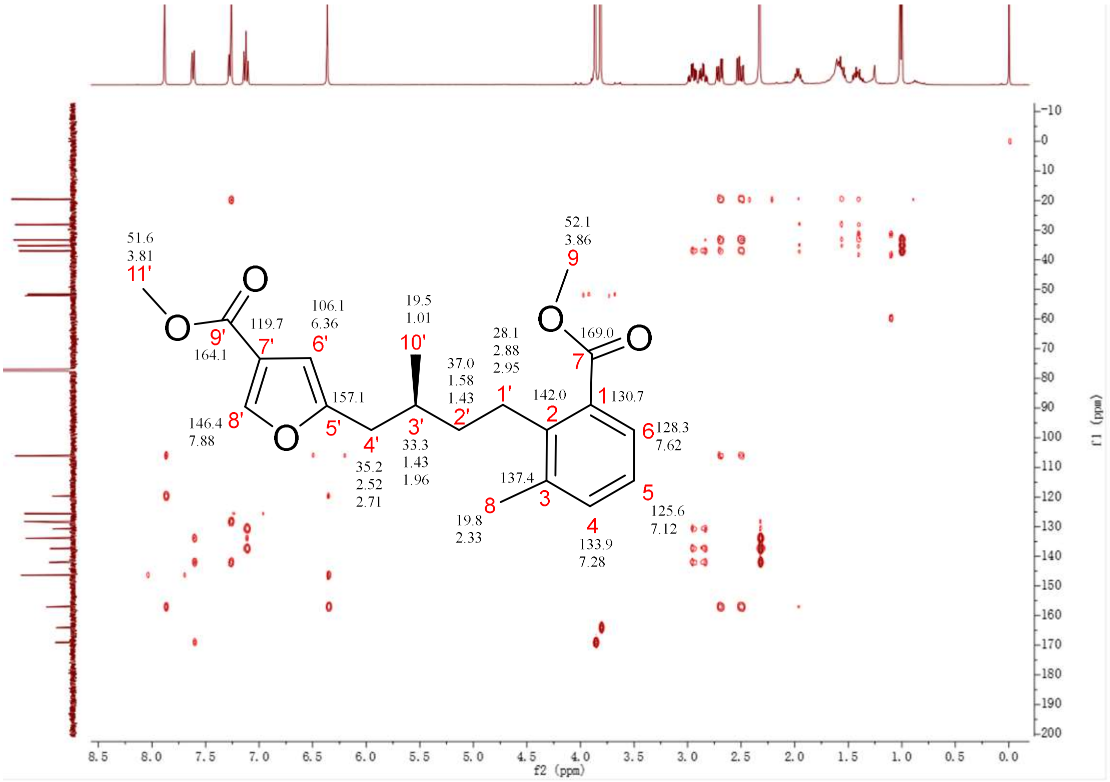

Figure S23. HMBC (125 MHz, $CDCl_3$) of **1**

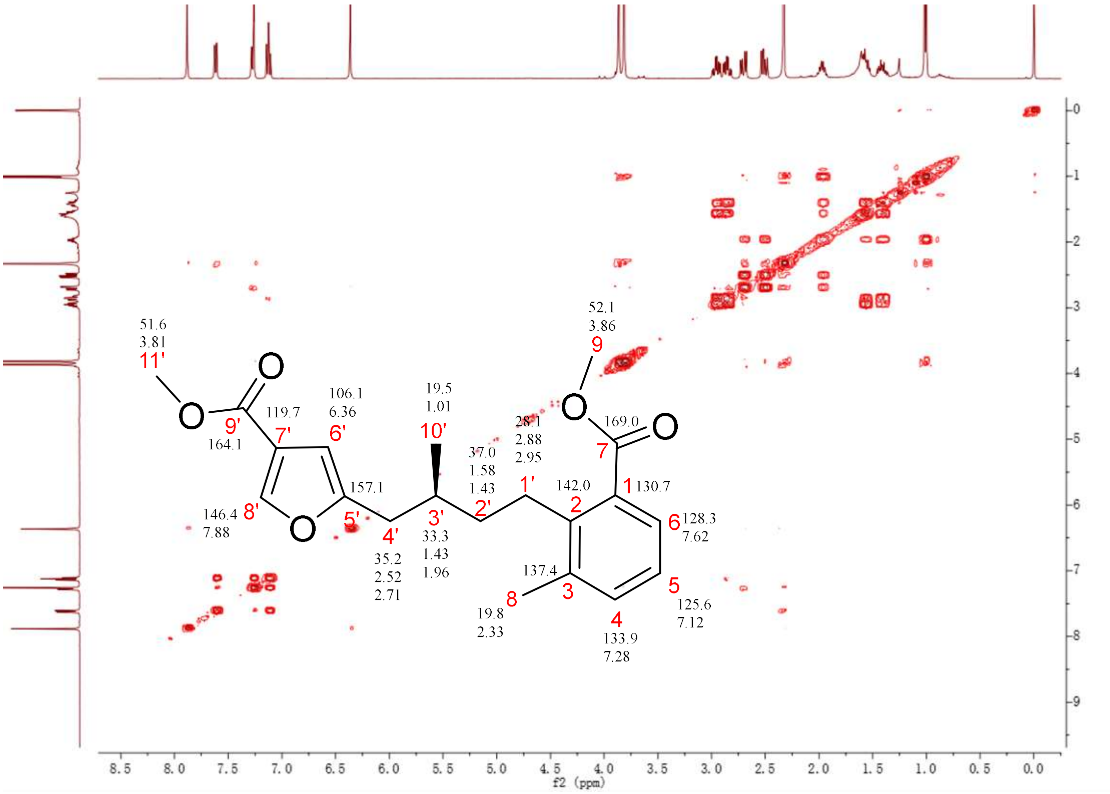

Figure S24. ^1H-^1H COSY (500 MHz, $CDCl_3$) of **1**

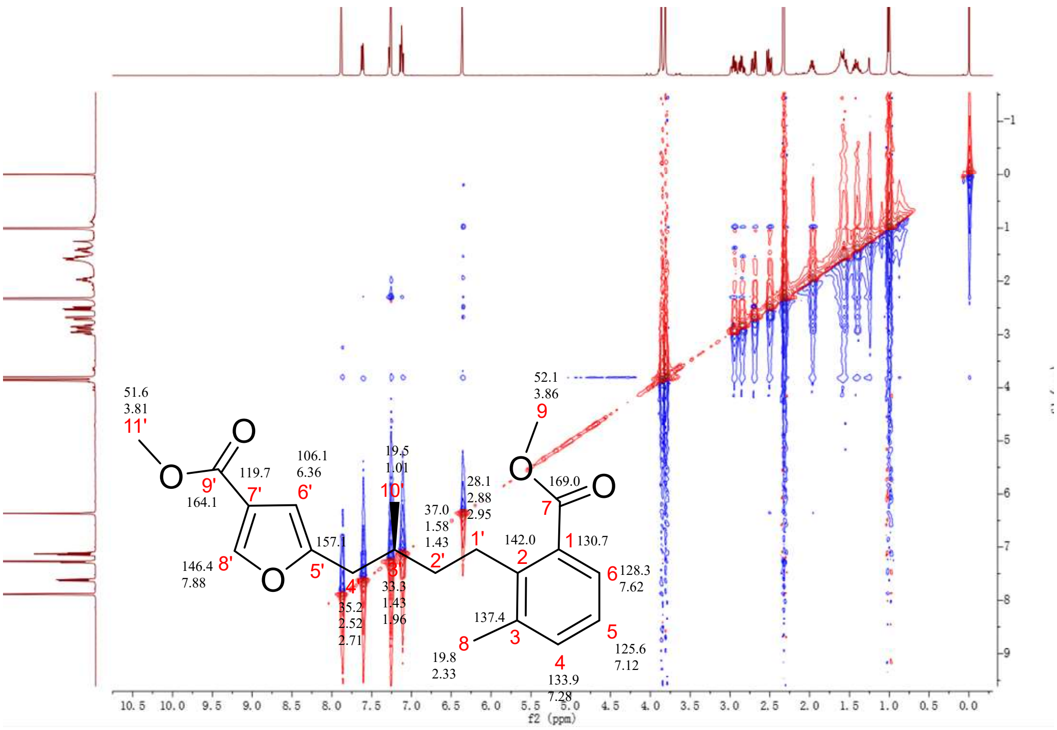

Figure S25. NOESY (500 MHz, $CDCl_3$) of **1**

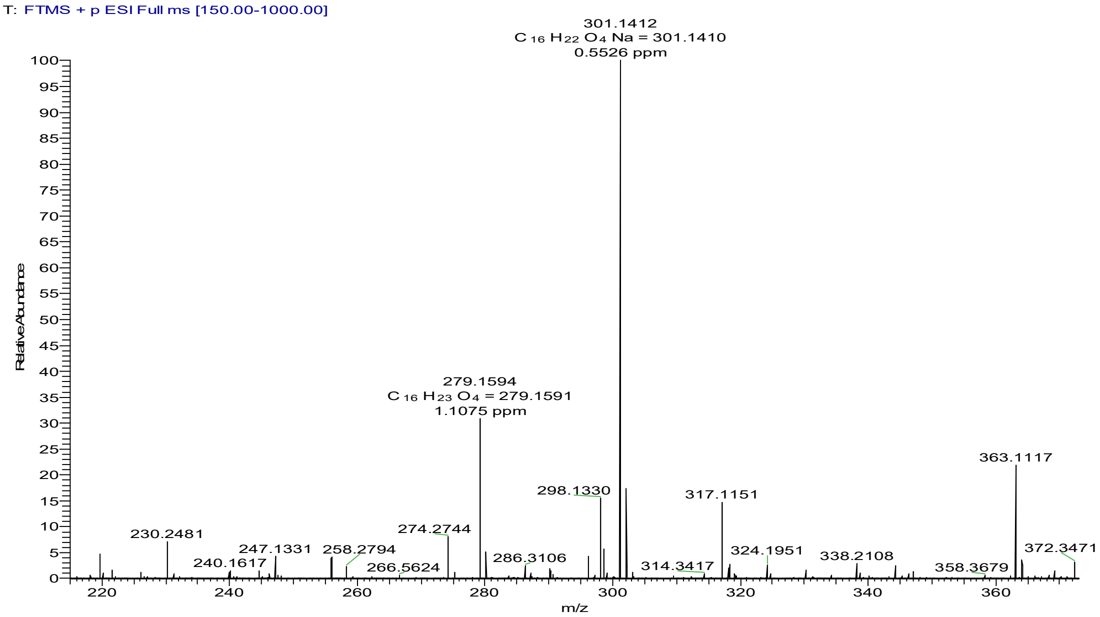

Figure S26. The positive HRESIMS spectrum of **2**

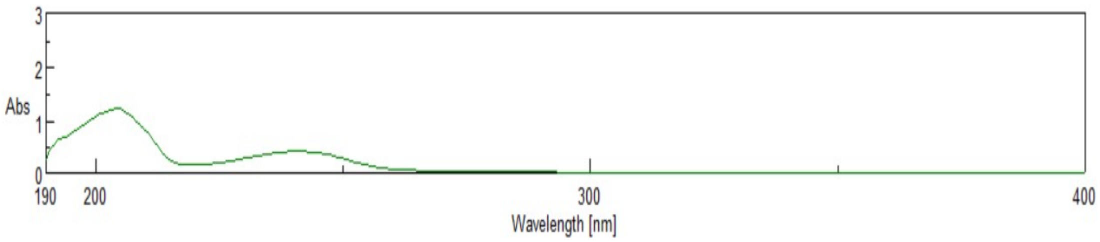

Figure S27. UV spectrum of **2**

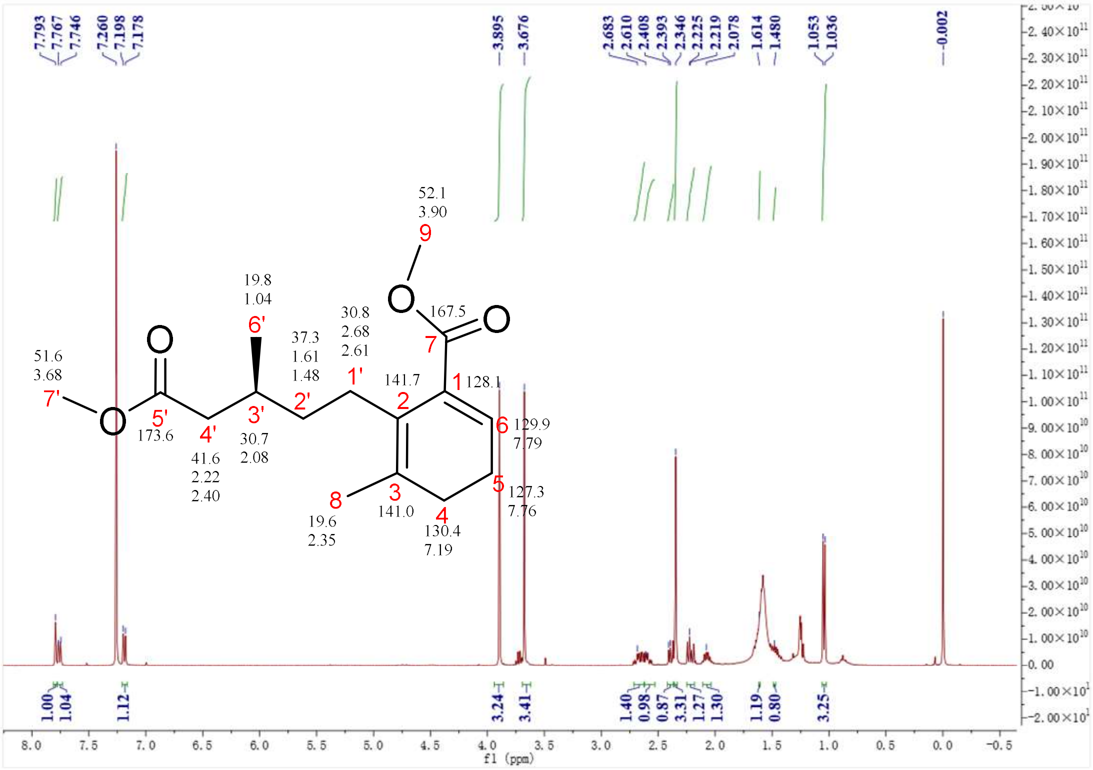

Figure S28. ^{1}H NMR spectrum (500 MHz, $CDCl_3$) of **2**

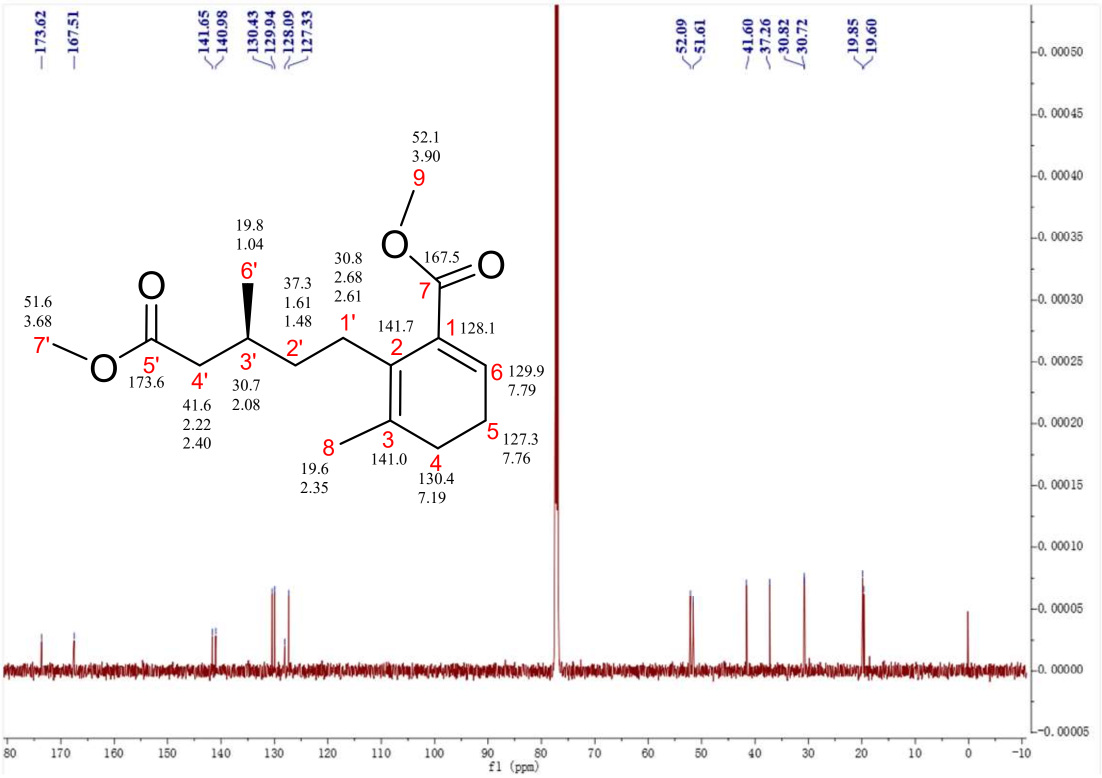

Figure S29. ^{13}C NMR spectrum (125 MHz, $CDCl_3$) of **2**

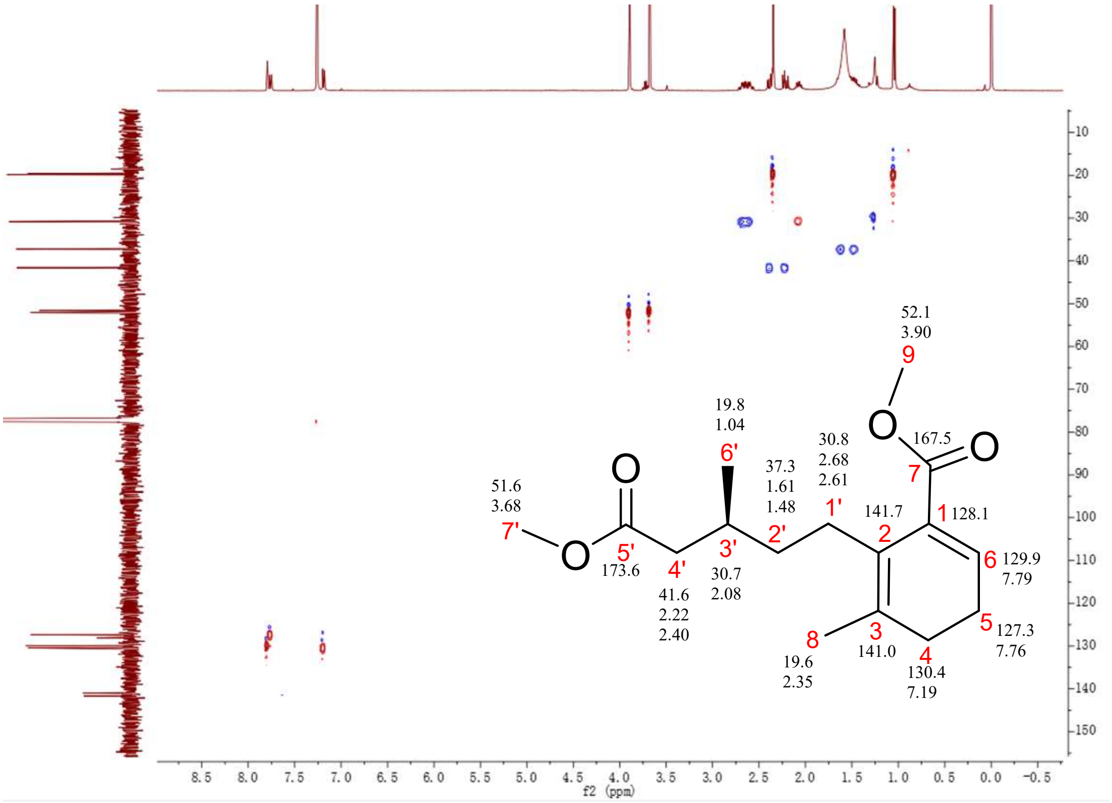

Figure S30. HSQC (500 M Hz, $CDCl_3$) of **2**

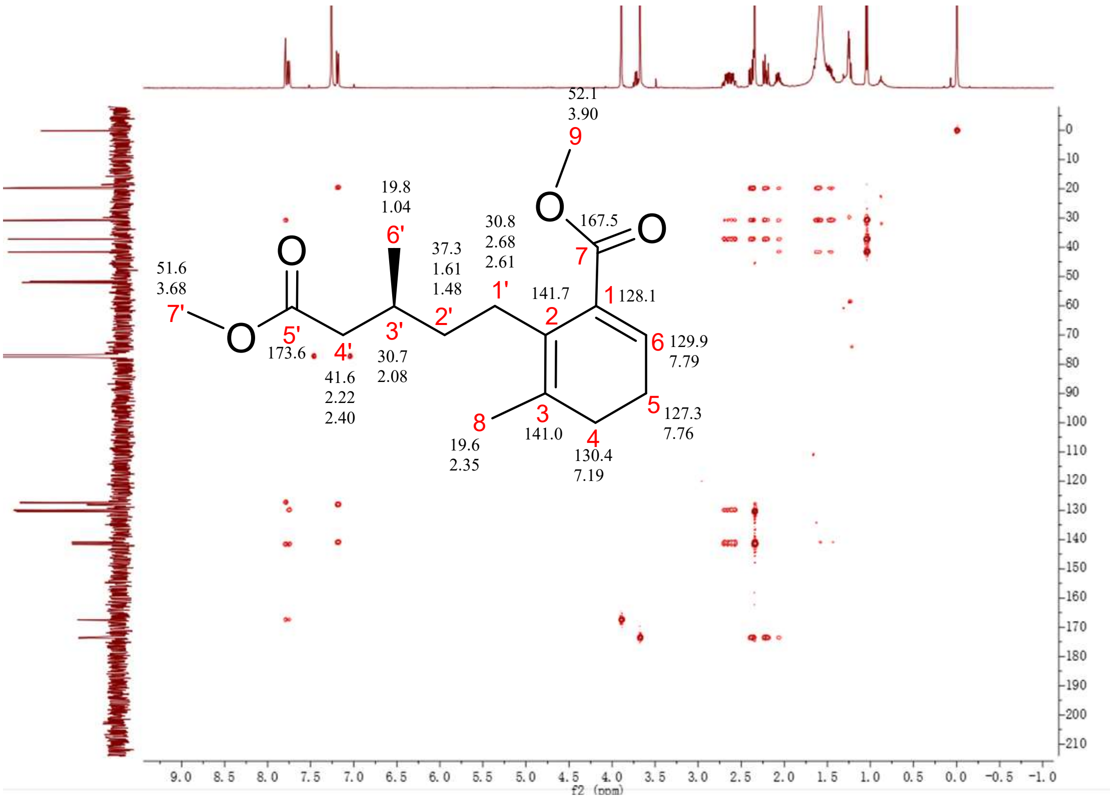

Figure S31. HMBC (125 MHz, $CDCl_3$) of **2**

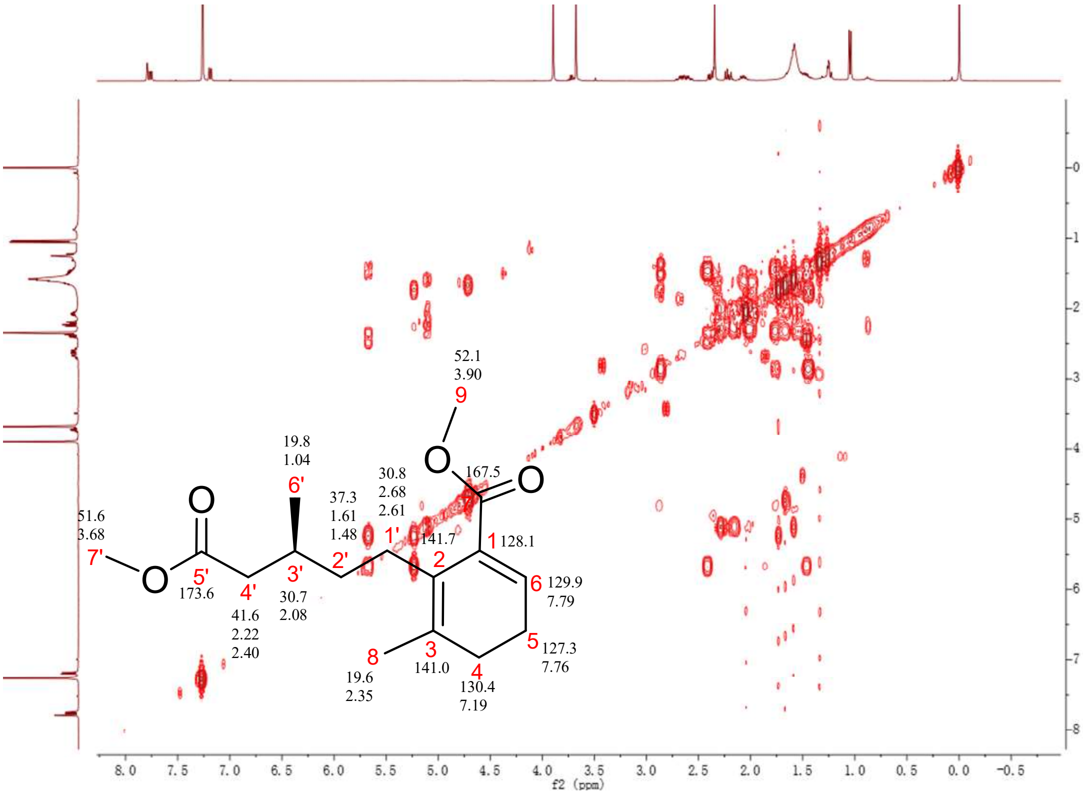

Figure S32. ${}^{1}H$-${}^{1}H$ COSY (500 MHz, $CDCl_3$) of **2**

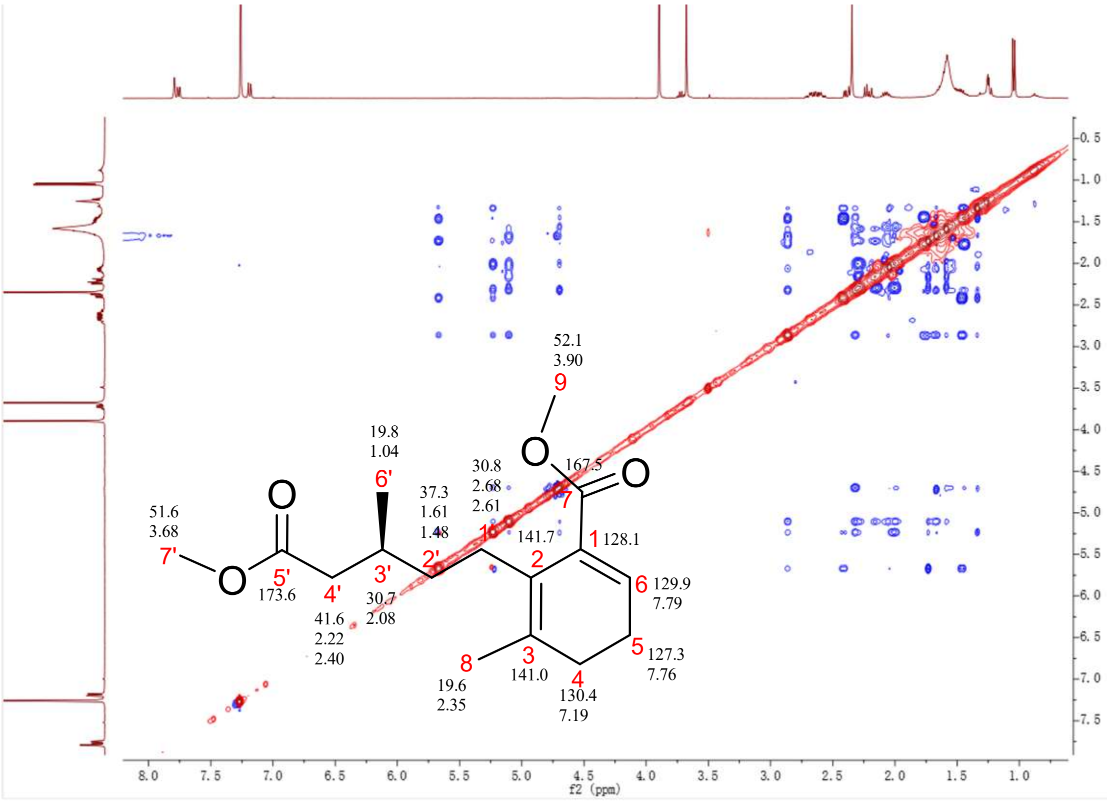

Figure S33. NOESY (500 MHz, $CDCl_3$) of **2**

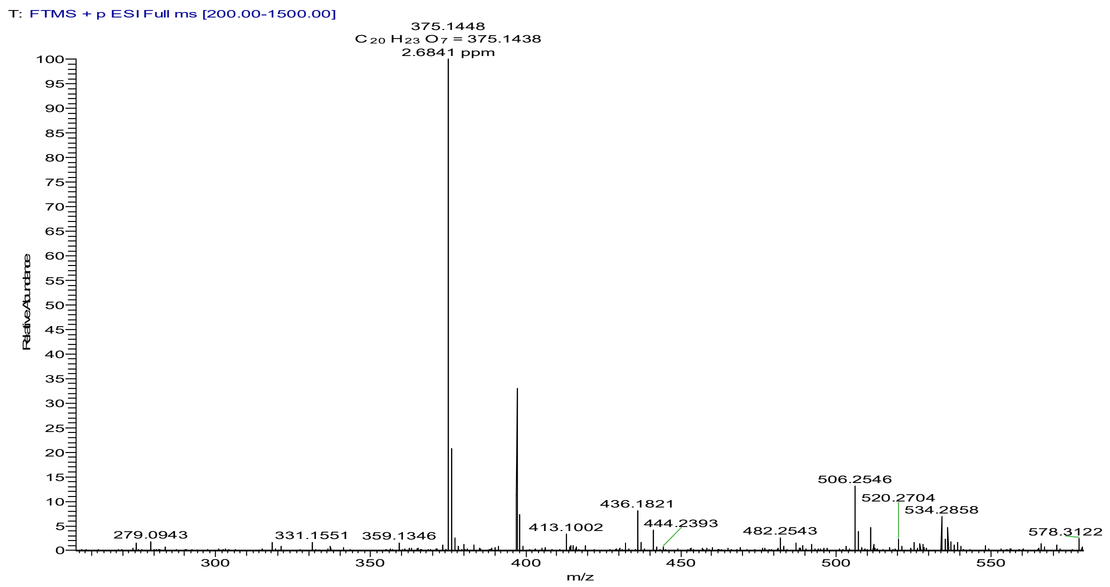

Figure S34. The positive HRESIMS spectrum of **3**

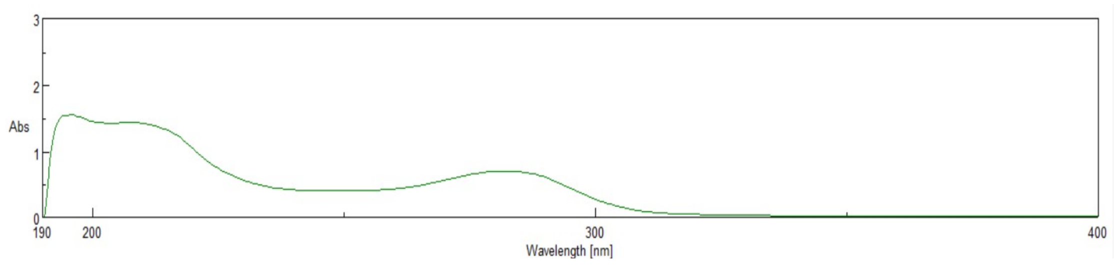

Figure S35. UV spectrum of compound **3**

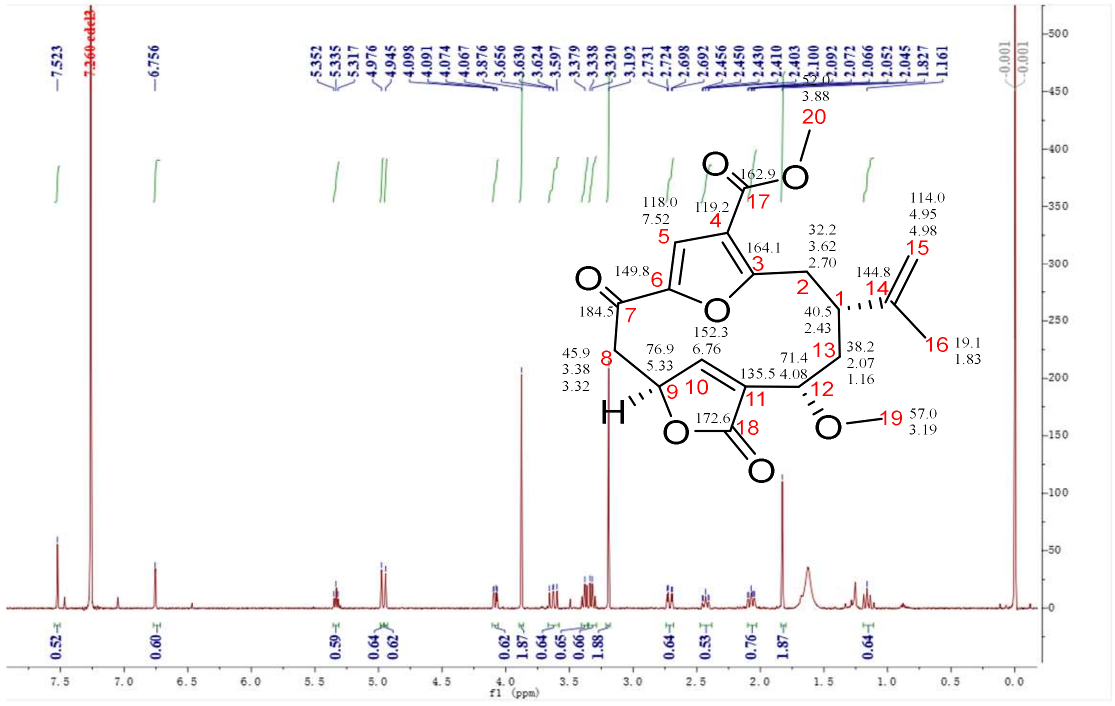

Figure S36. ^{1}H NMR spectrum (500 MHz, $CDCl_3$) of **3**

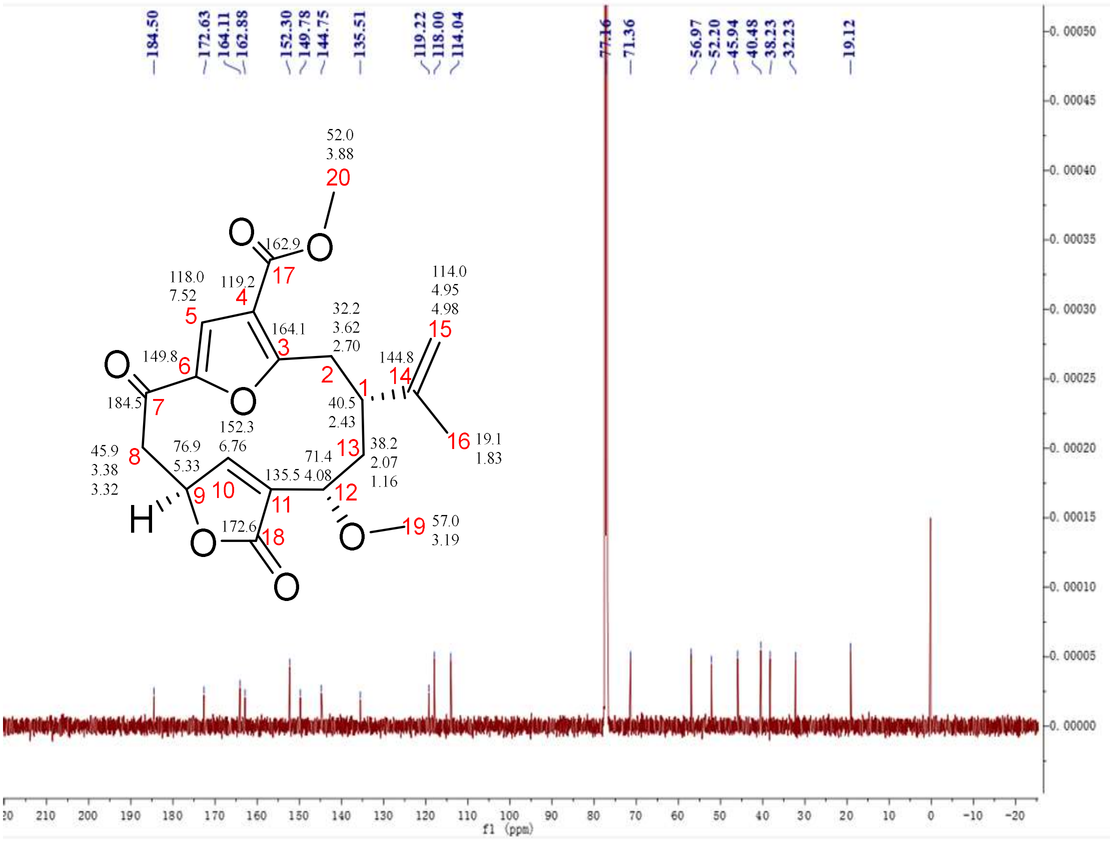

Figure S37. ^{13}C NMR spectrum (125 MHz, CDCl$_3$) of **3**

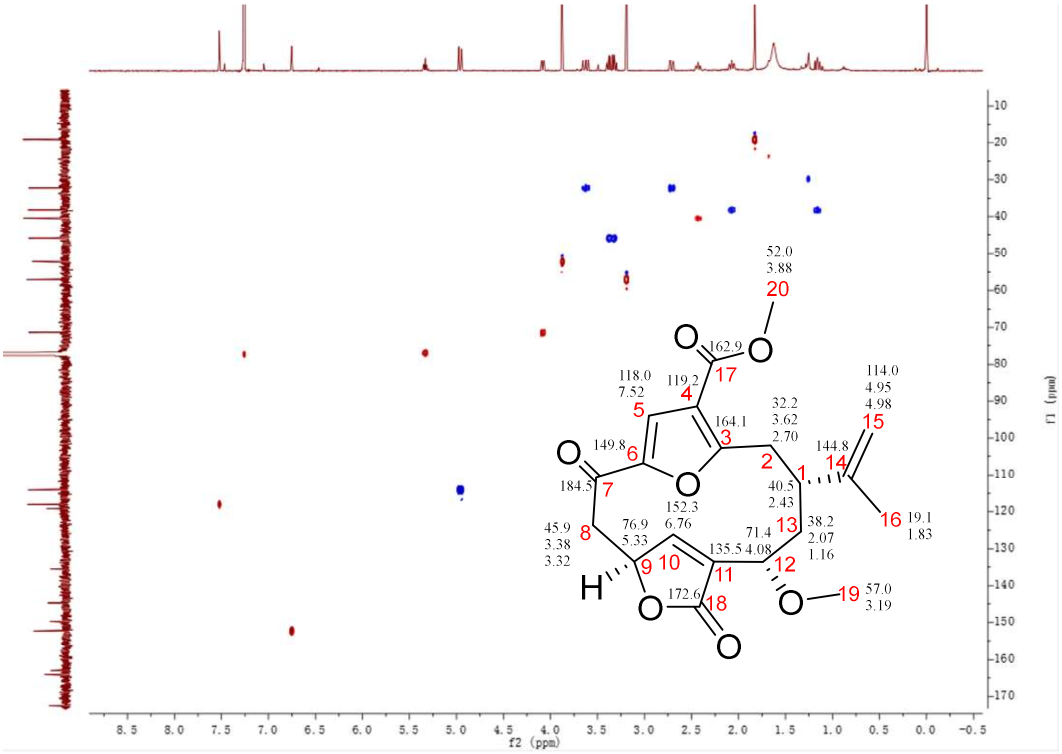

Figure S38. HSQC (500 M Hz, CDCl$_3$) of **3**

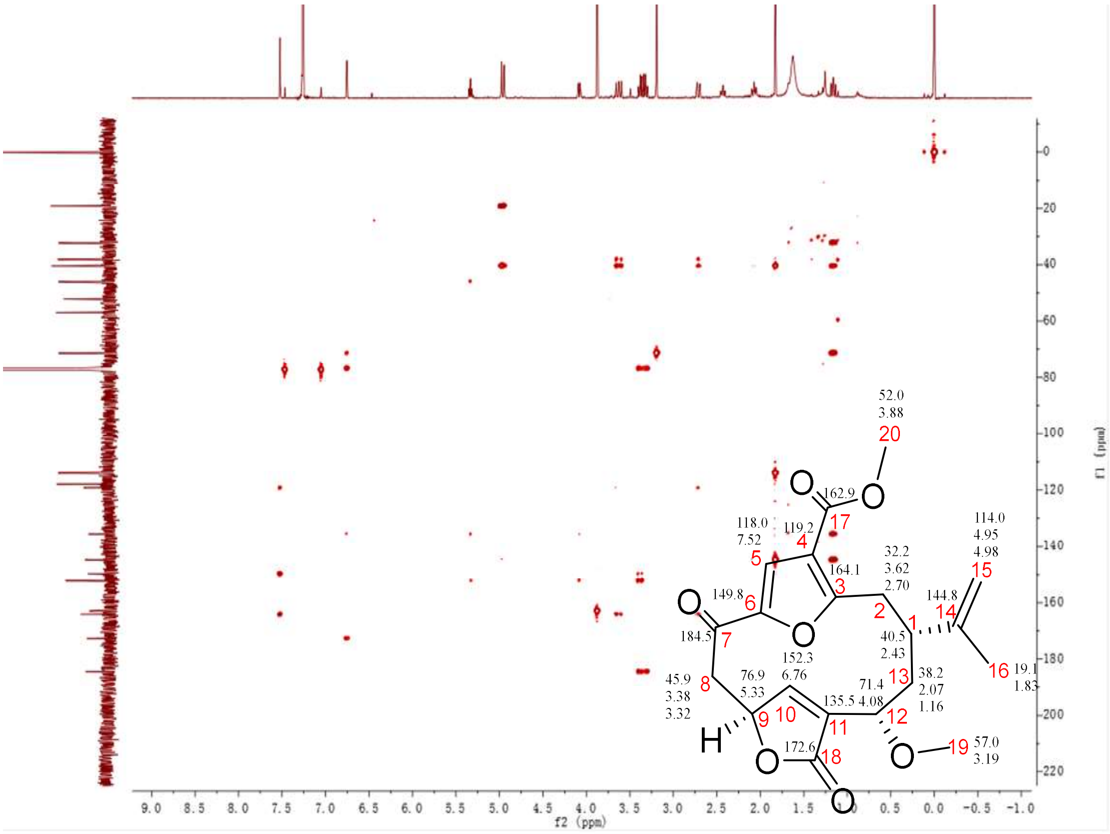

Figure S39. HMBC (125 MHz, $CDCl_3$) of **3**

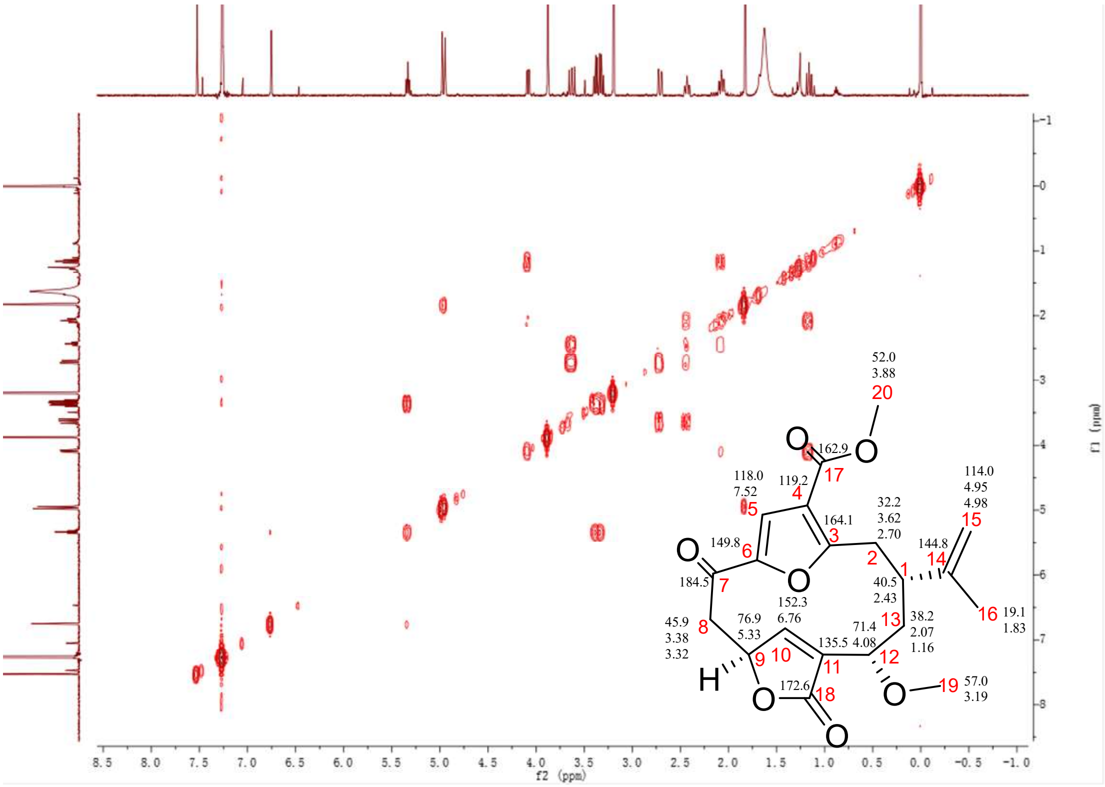

Figure S40. ^1H-^1H COSY (500 MHz, $CDCl_3$) of **3**

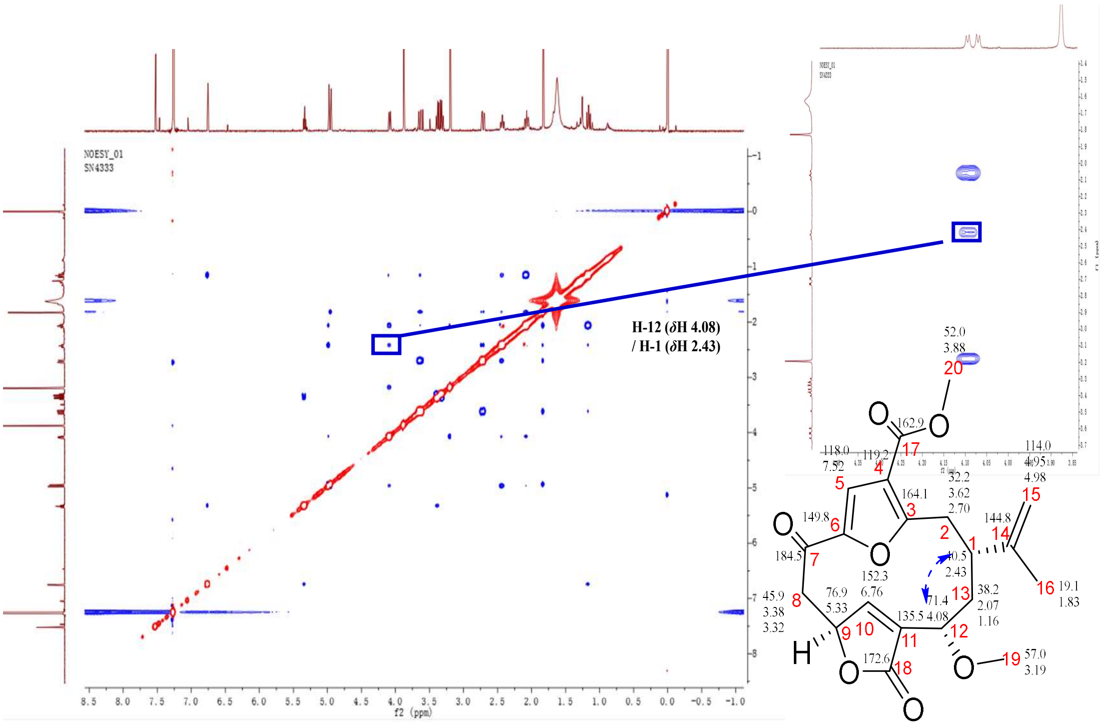

Figure S41. NOESY (500 MHz, $CDCl_3$) of **3**

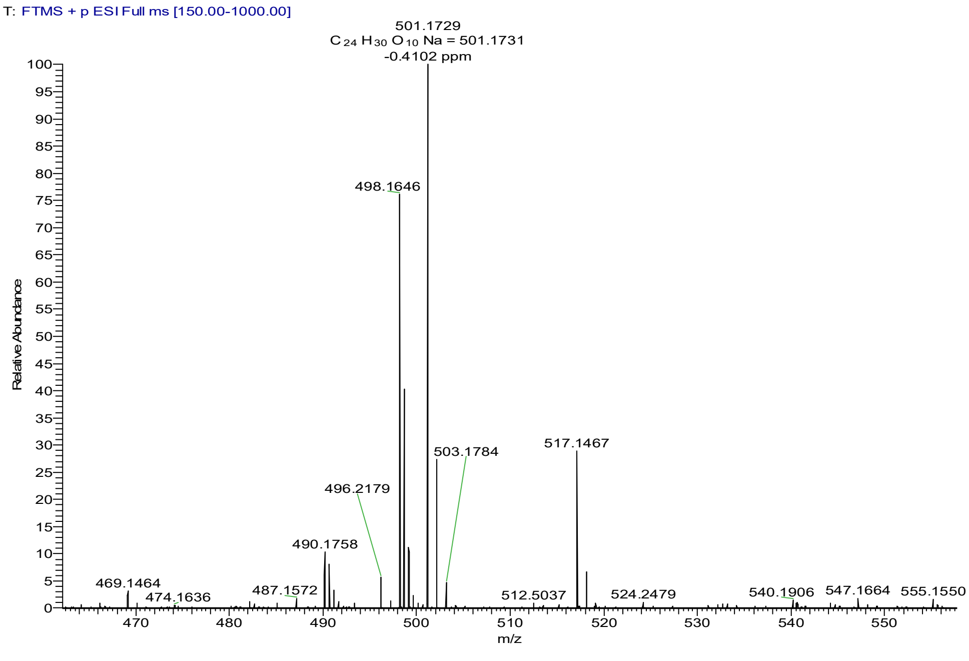

Figure S42. The positive HRESIMS spectrum of **4**

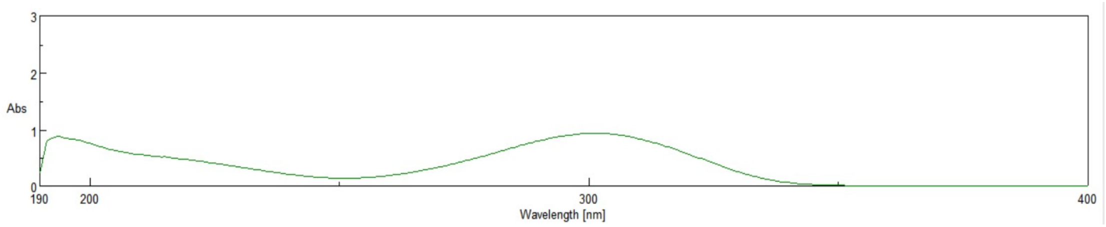

Figure S43. UV spectrum of **4**

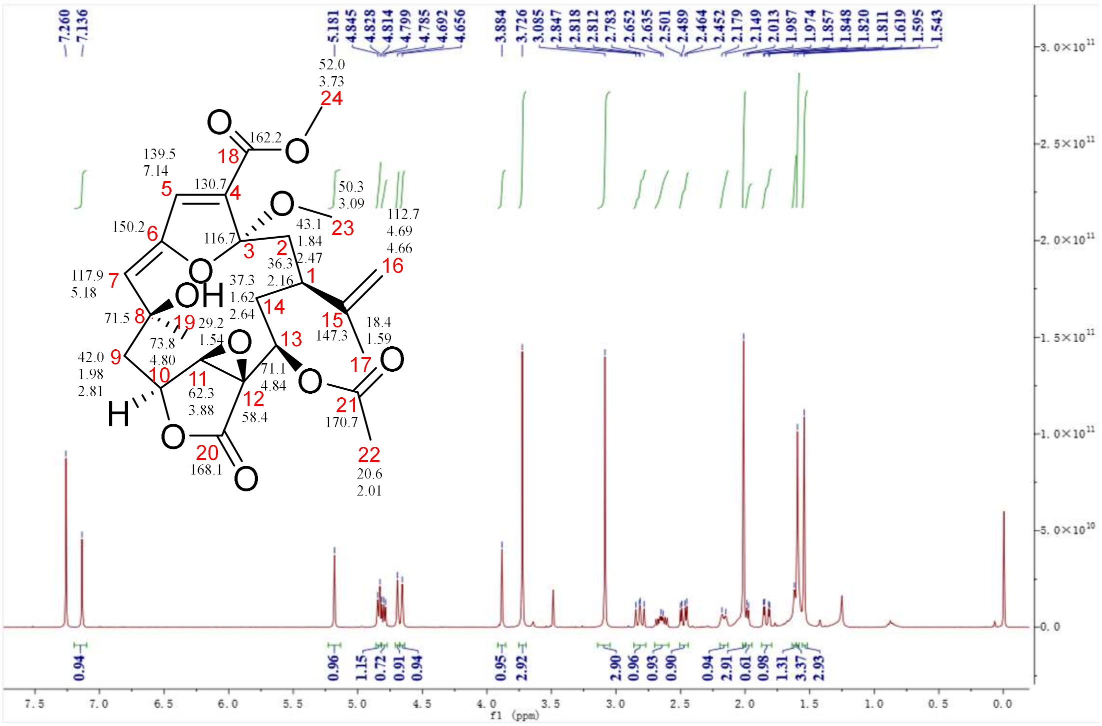

Figure S44. ^{1}H NMR spectrum (500 MHz, $CDCl_3$) of **4**

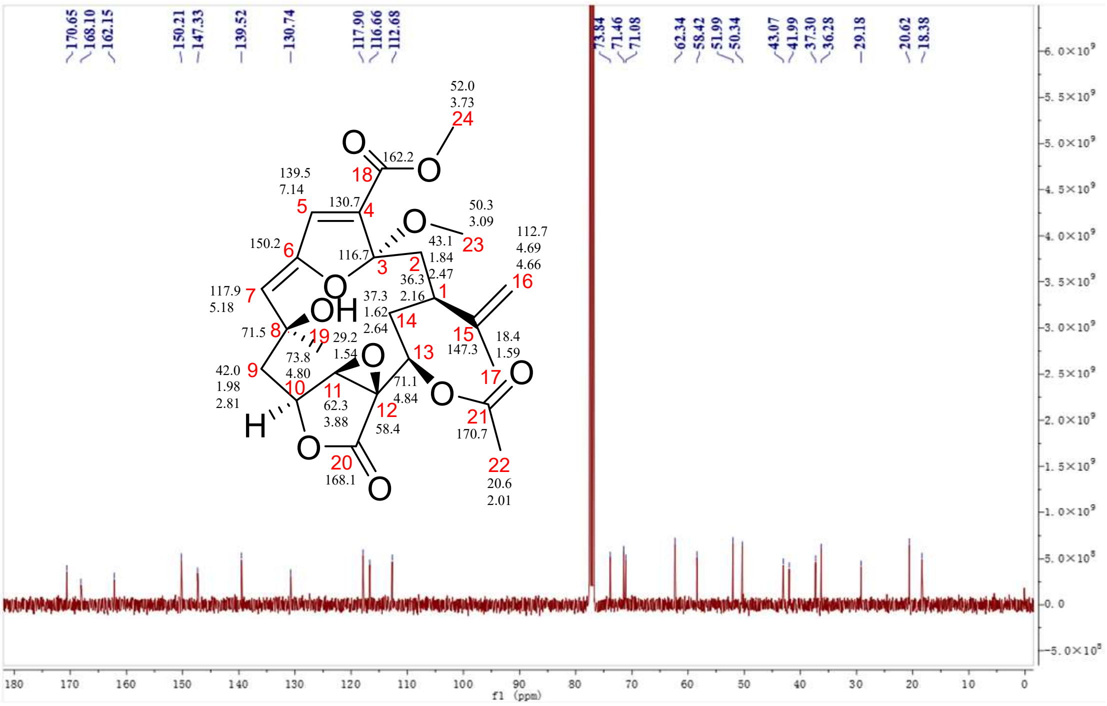

Figure S45. ^{13}C NMR spectrum (125 MHz, $CDCl_3$) of **4**

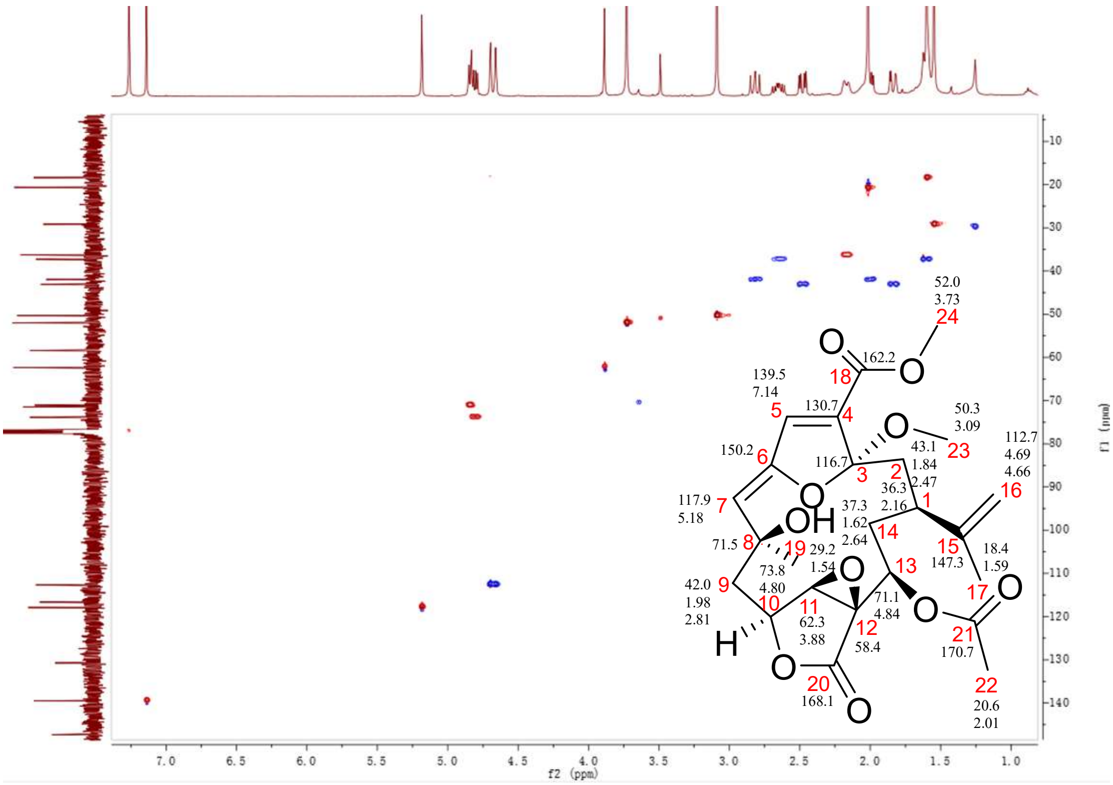

Figure S46. HSQC (500 M Hz, $CDCl_3$) of **4**

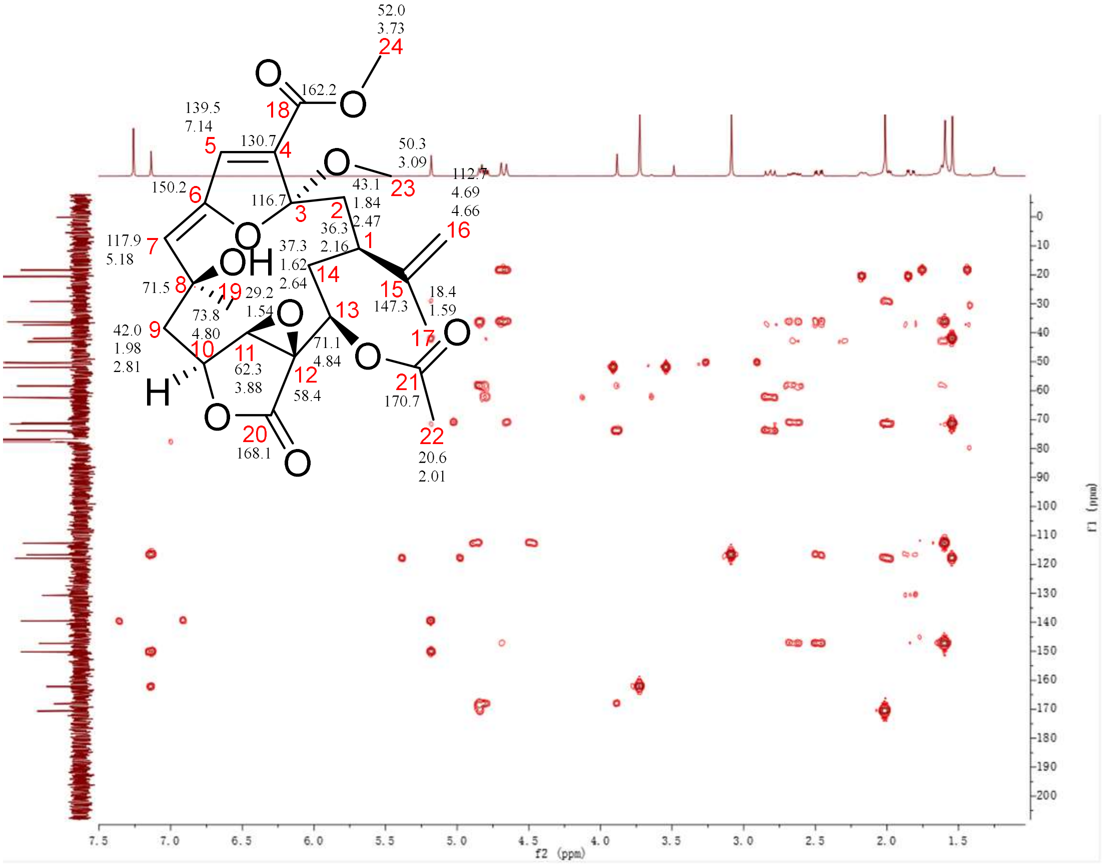

Figure S47. HMBC (125 MHz, $CDCl_3$) of **4**

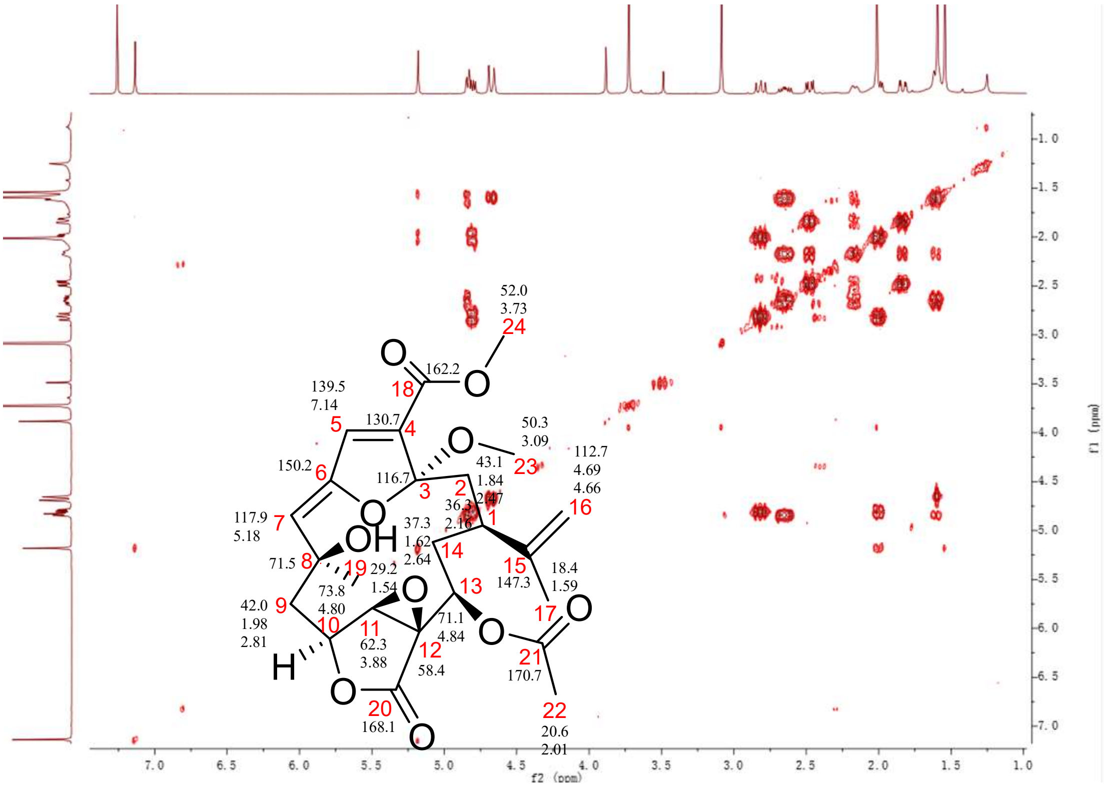

Figure S48. ^{1}H-^{1}H COSY (500 MHz, $CDCl_3$) of **4**

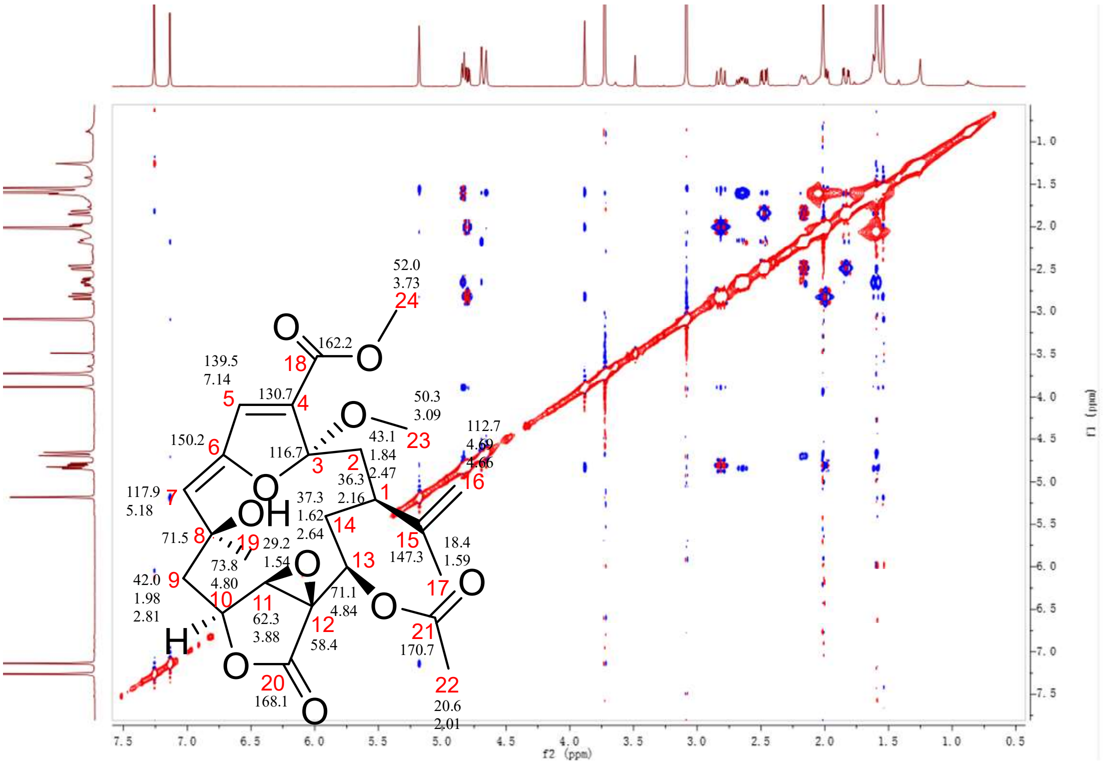

Figure S49. NOESY (500 MHz, $CDCl_3$) of **4**

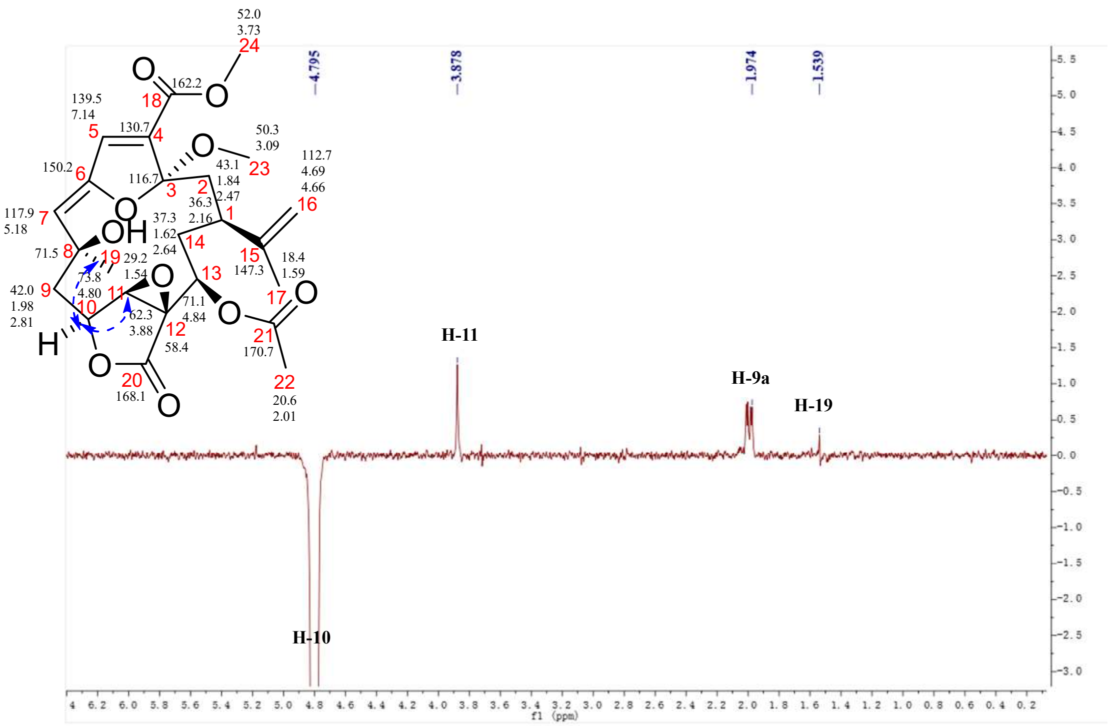

Figure S50. NOESY (500 MHz, $CDCl_3$) of **4**

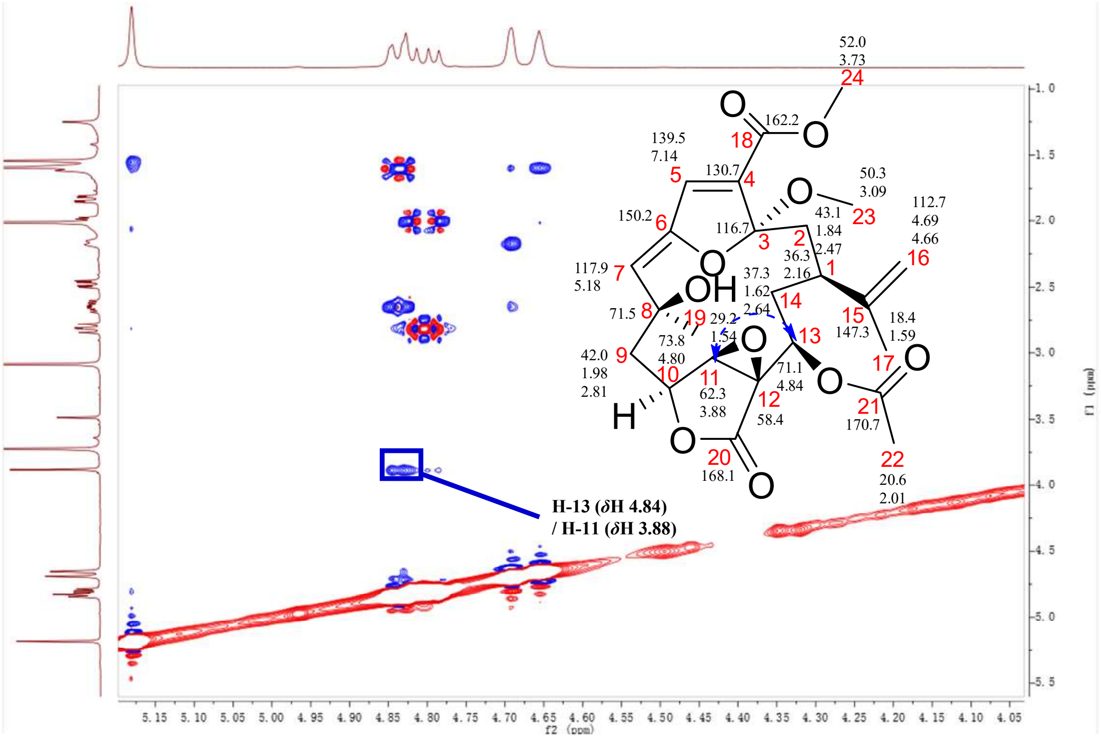

Figure S51. NOESY (500 MHz, $CDCl_3$) of **4**

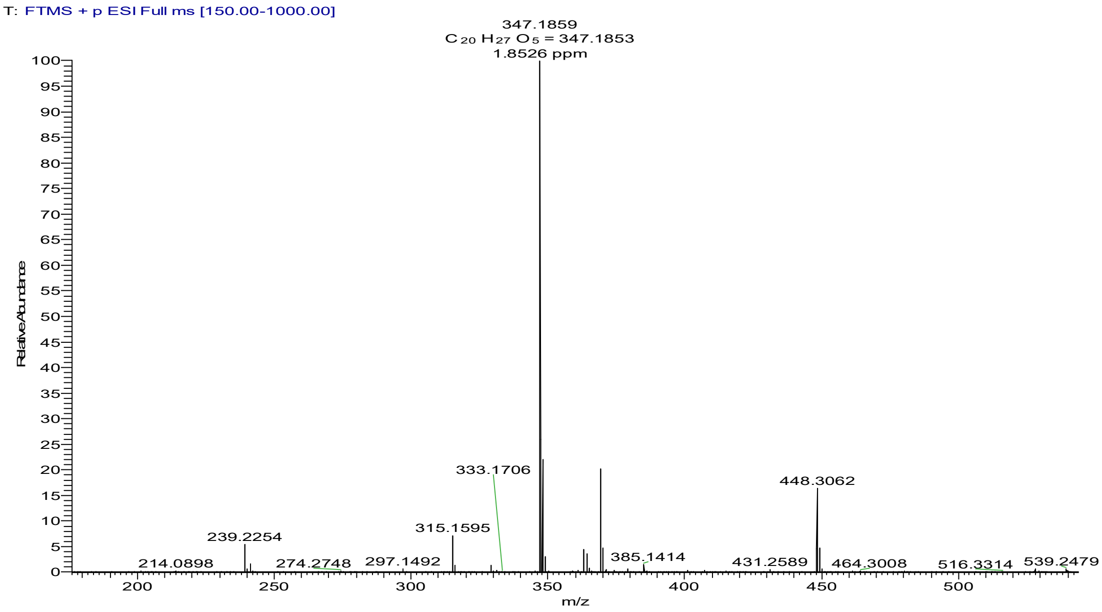

Figure S52. The positive HRESIMS spectrum of **5**

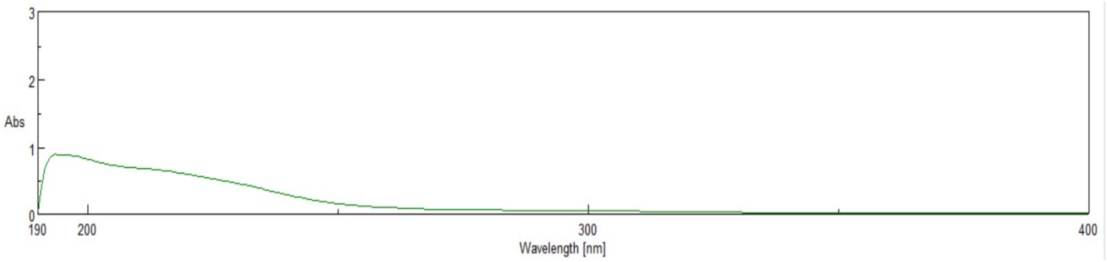

Figure S53. UV spectrum of compound **5**

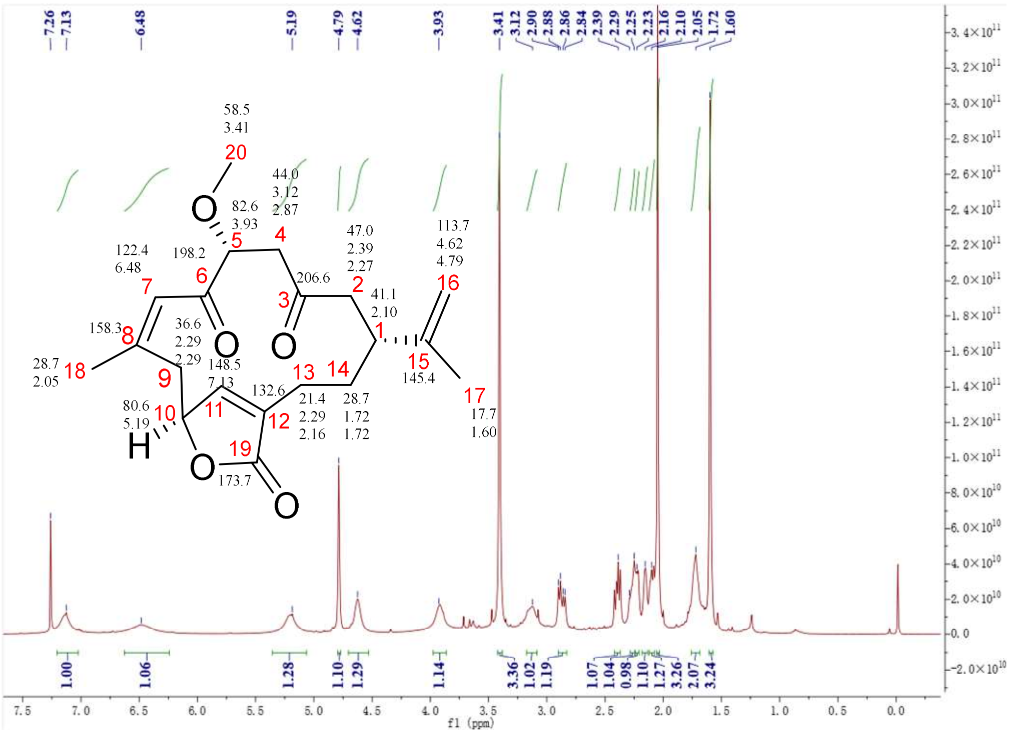

Figure S54. ^{1}H NMR spectrum (500 MHz, $CDCl_3$) of **5**

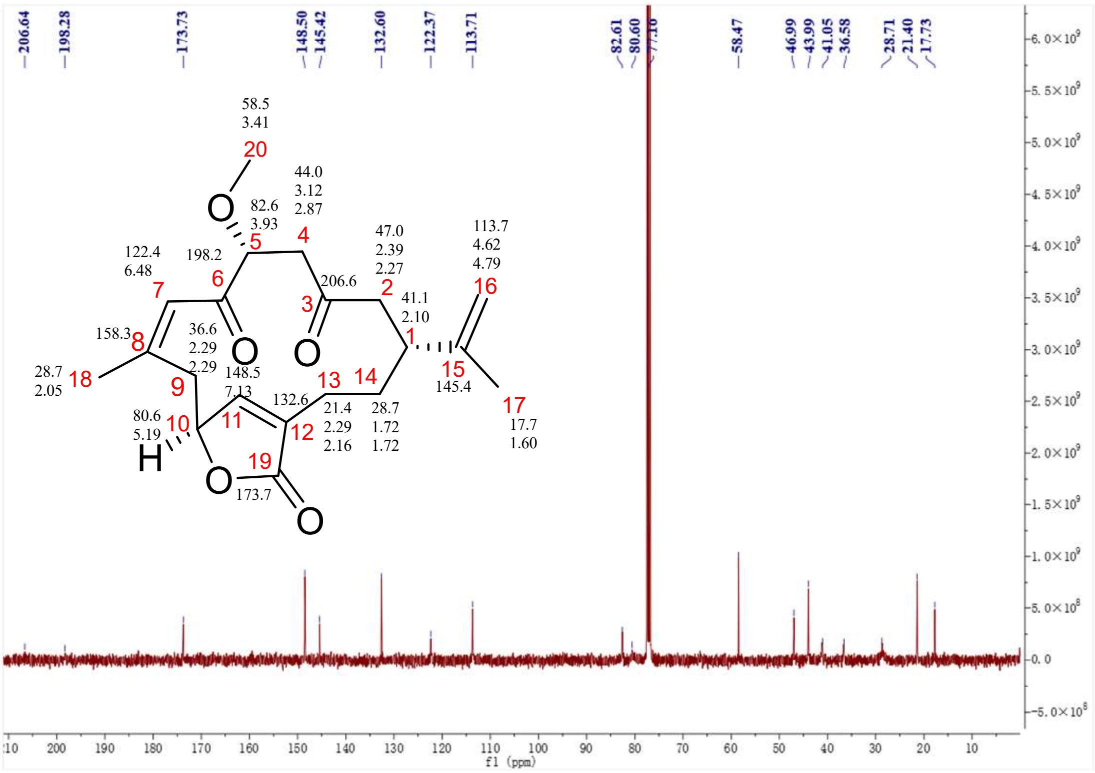

Figure S55. ^{13}C NMR spectrum (125 MHz, $CDCl_3$) of **5**

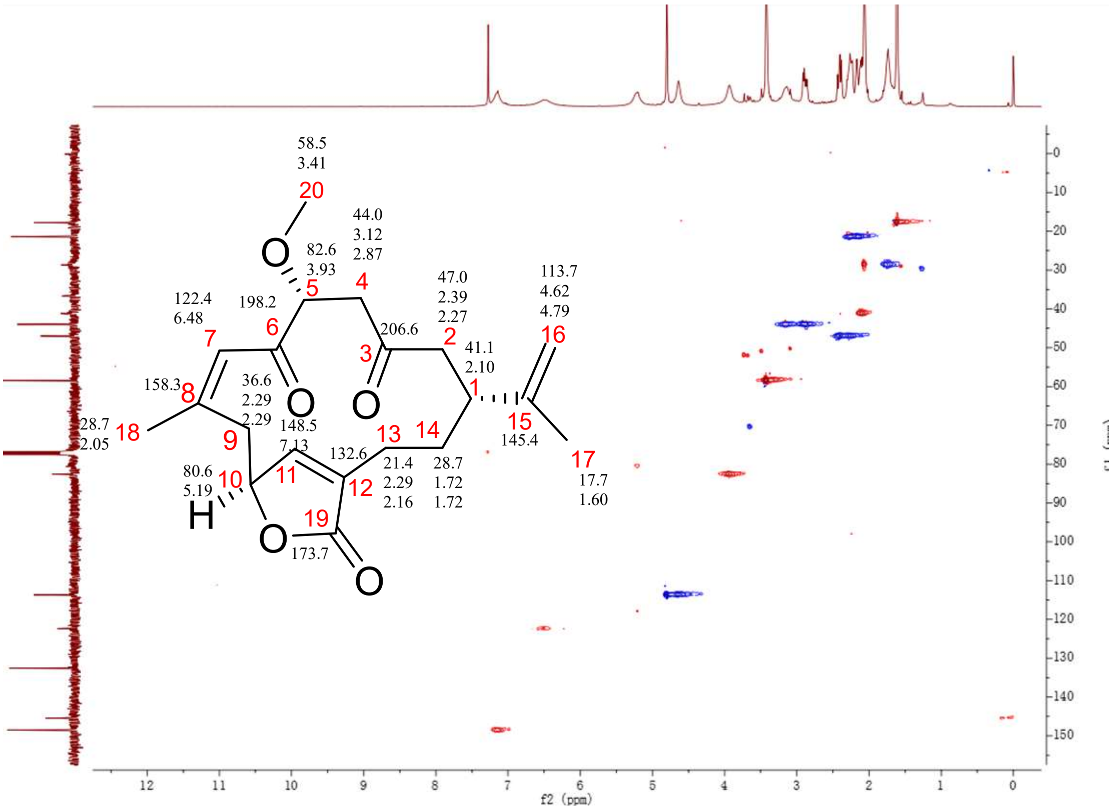

Figure S56. HSQC (500 M Hz, $CDCl_3$) of **5**

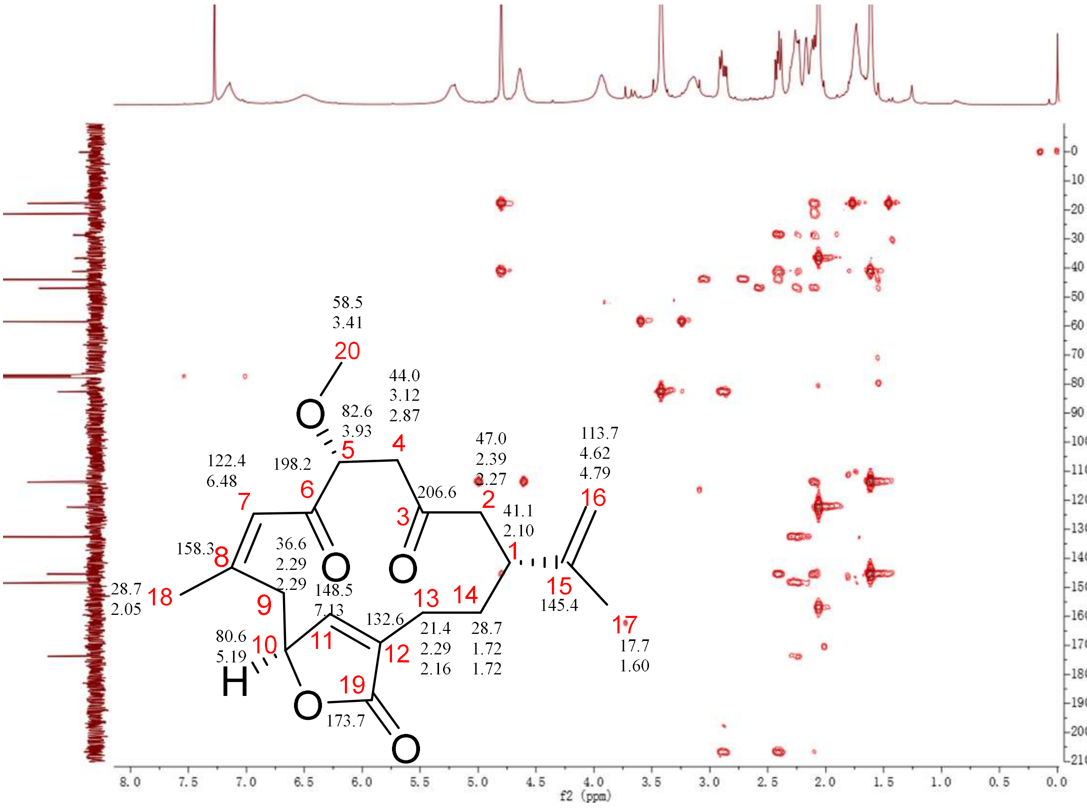

Figure S57. HMBC (125 MHz, $CDCl_3$) of **5**

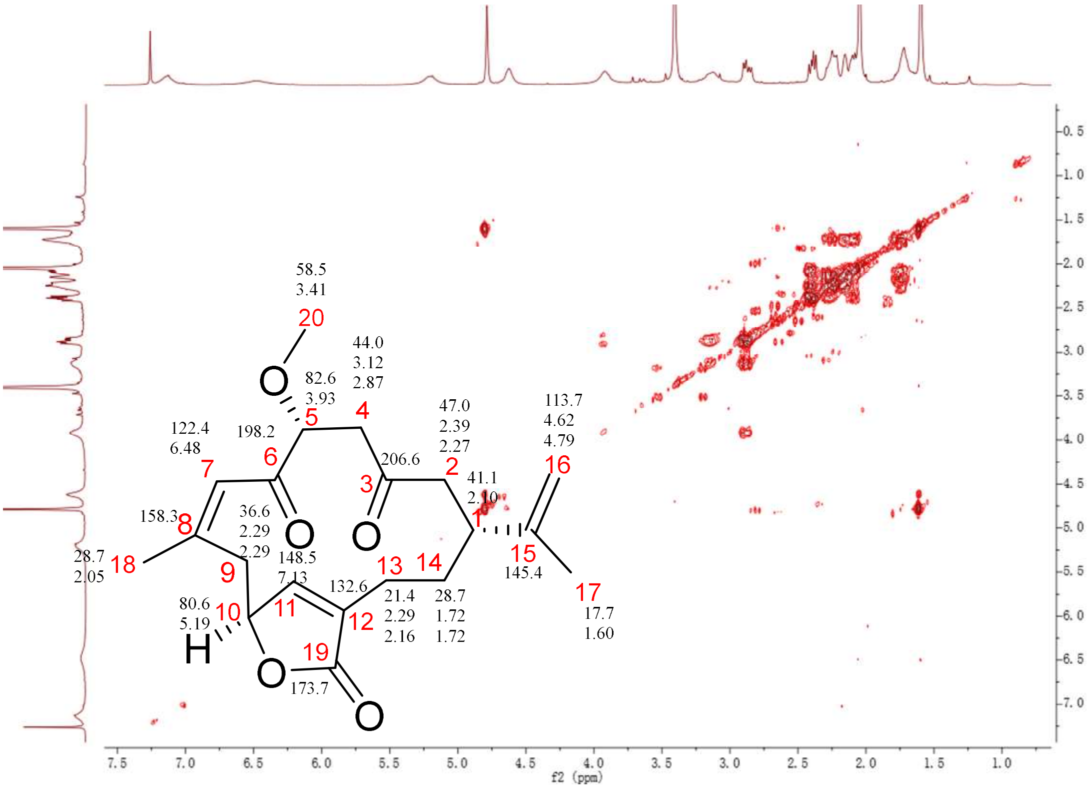

Figure S58. ^{1}H-^{1}H COSY (500 MHz, $CDCl_3$) of **5**

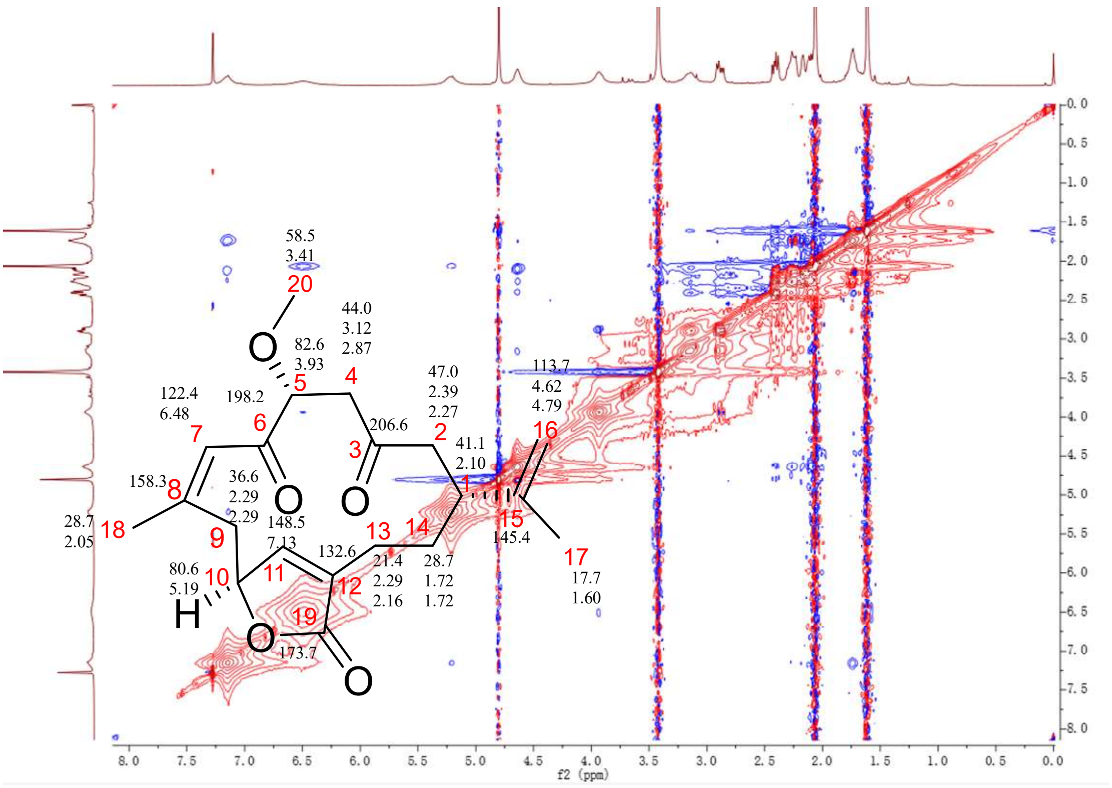

Figure S59. NOESY (500 MHz, $CDCl_3$) of **5**

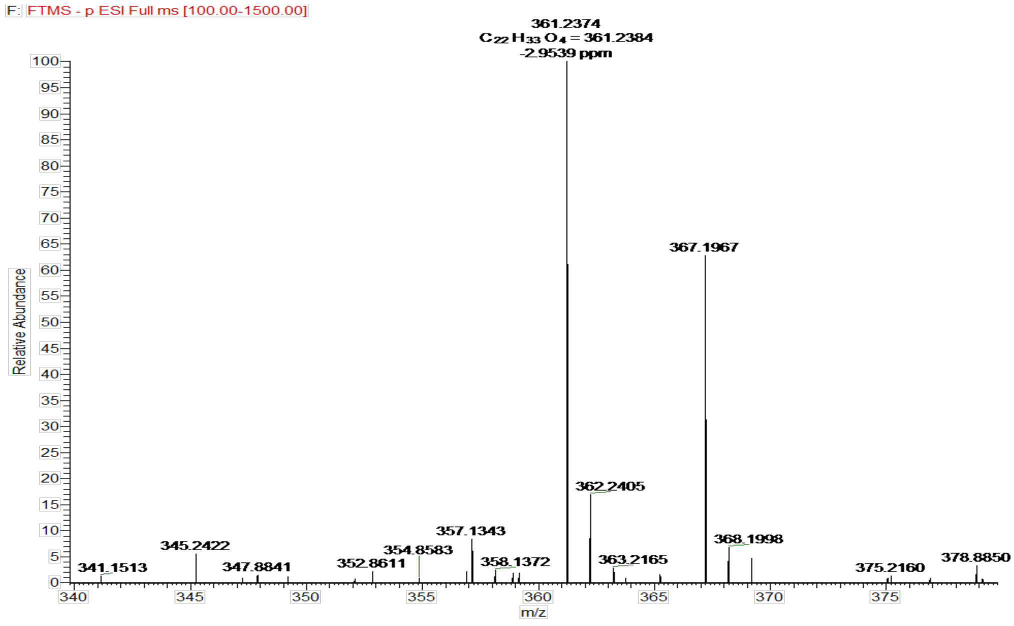

Figure S60. The positive HRESIMS spectrum of **6**

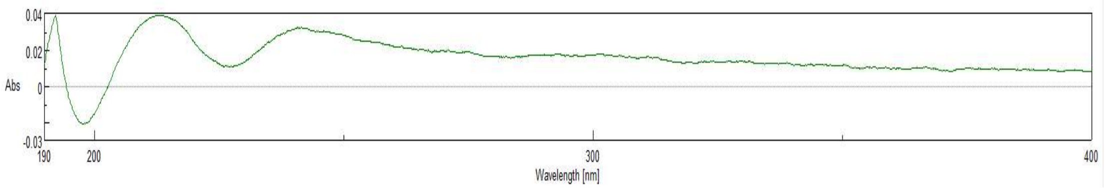

Figure S61. UV spectrum of **6**

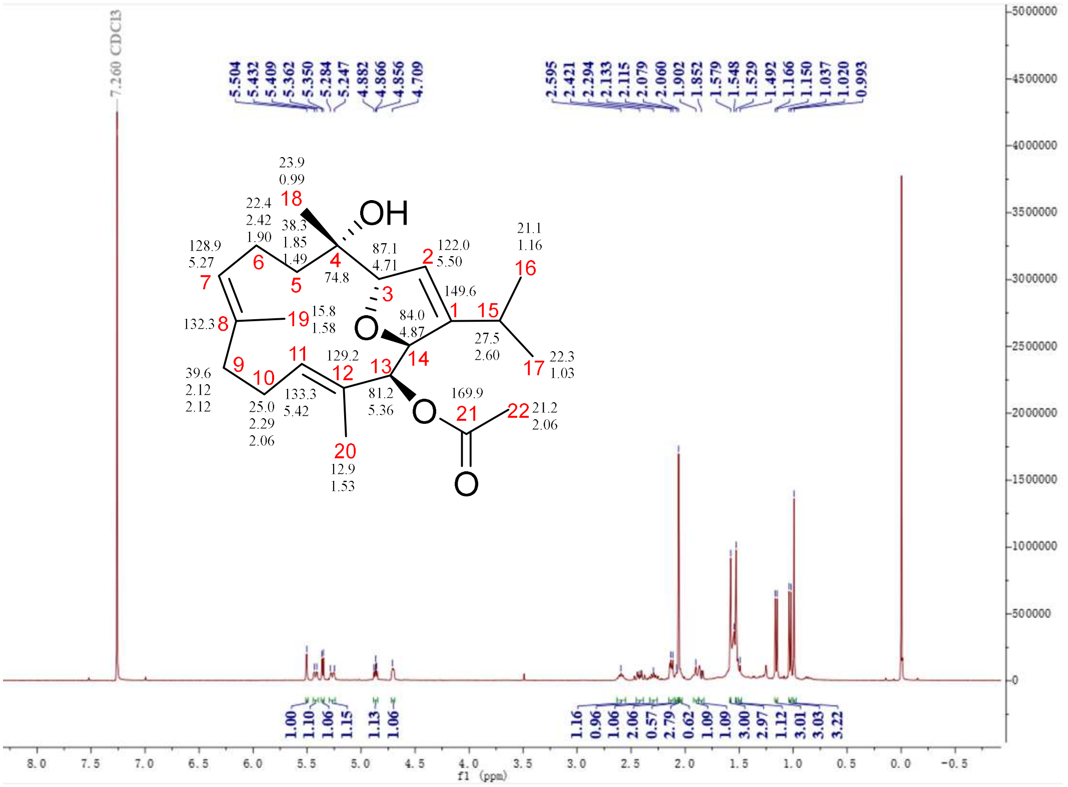

Figure S62. ^{1}H NMR spectrum (400 MHz, $CDCl_3$) of **6**

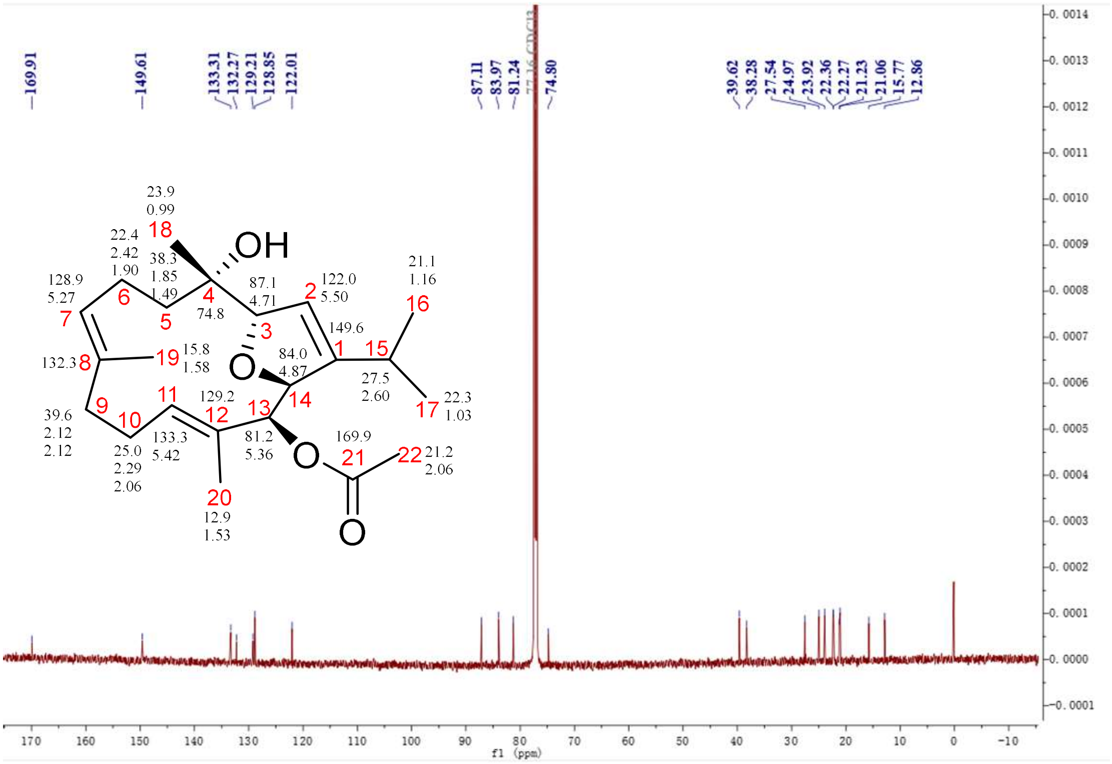

Figure S63. ^{13}C NMR spectrum (125 MHz, $CDCl_3$) of **6**

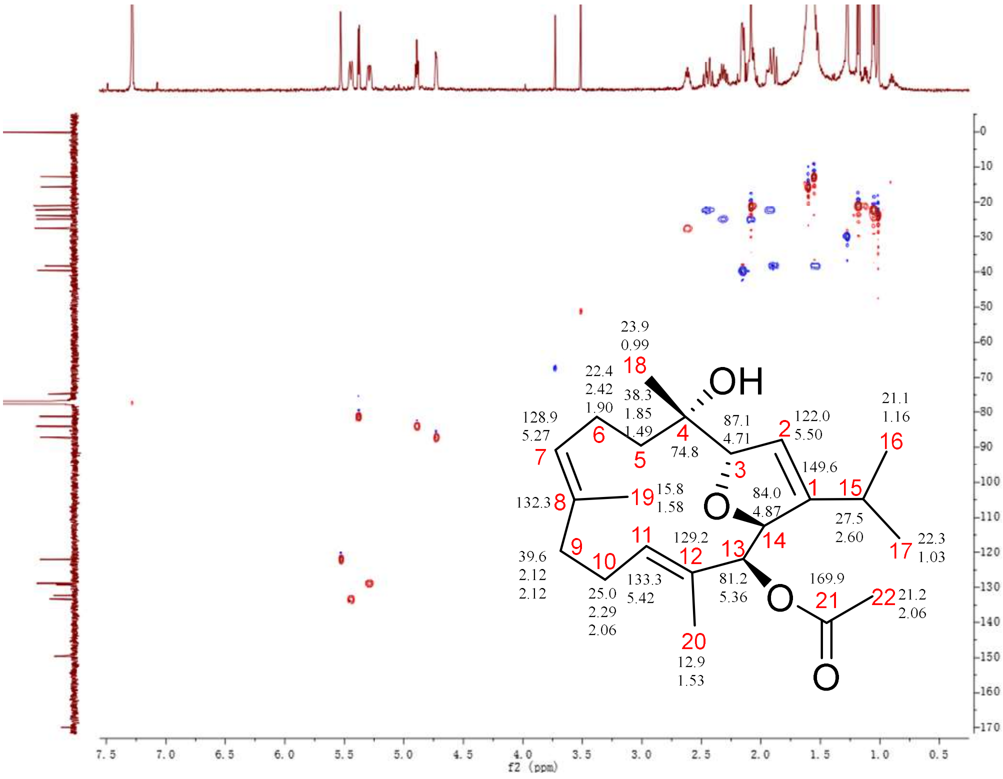

Figure S64. HSQC (500 M Hz, $CDCl_3$) of **6**

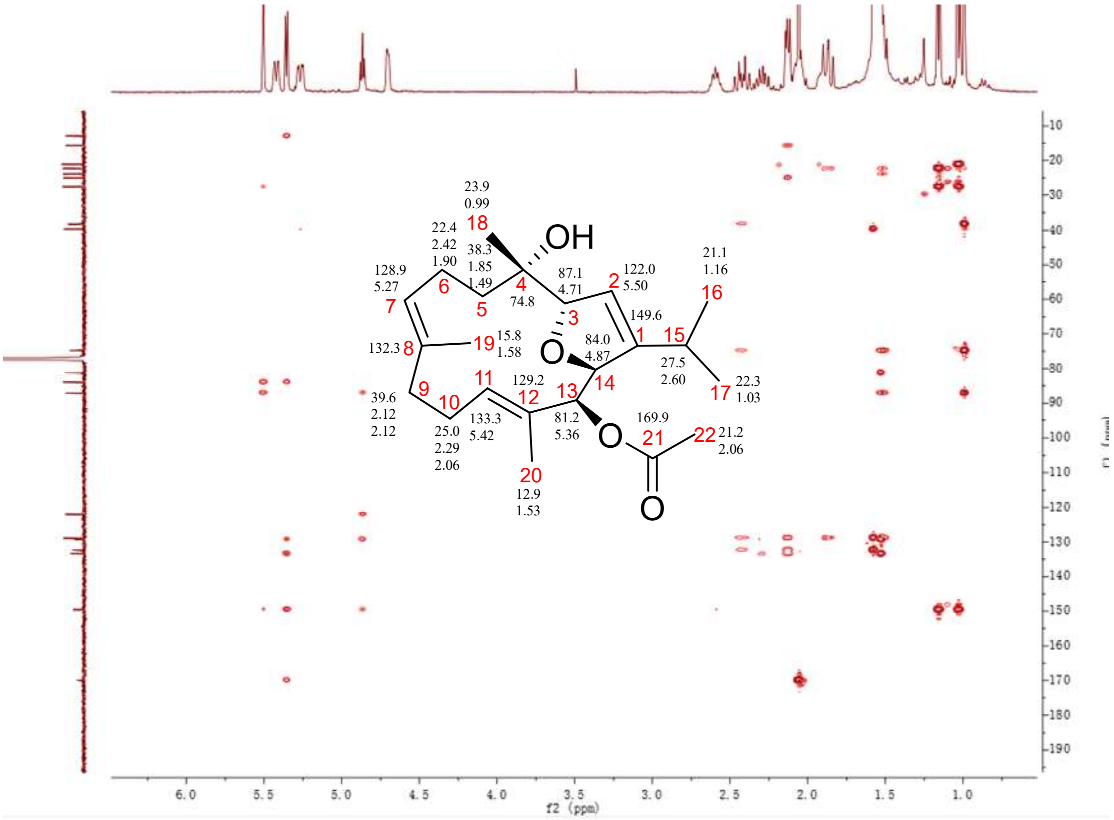

Figure S65. HMBC (125 MHz, $CDCl_3$) of **6**

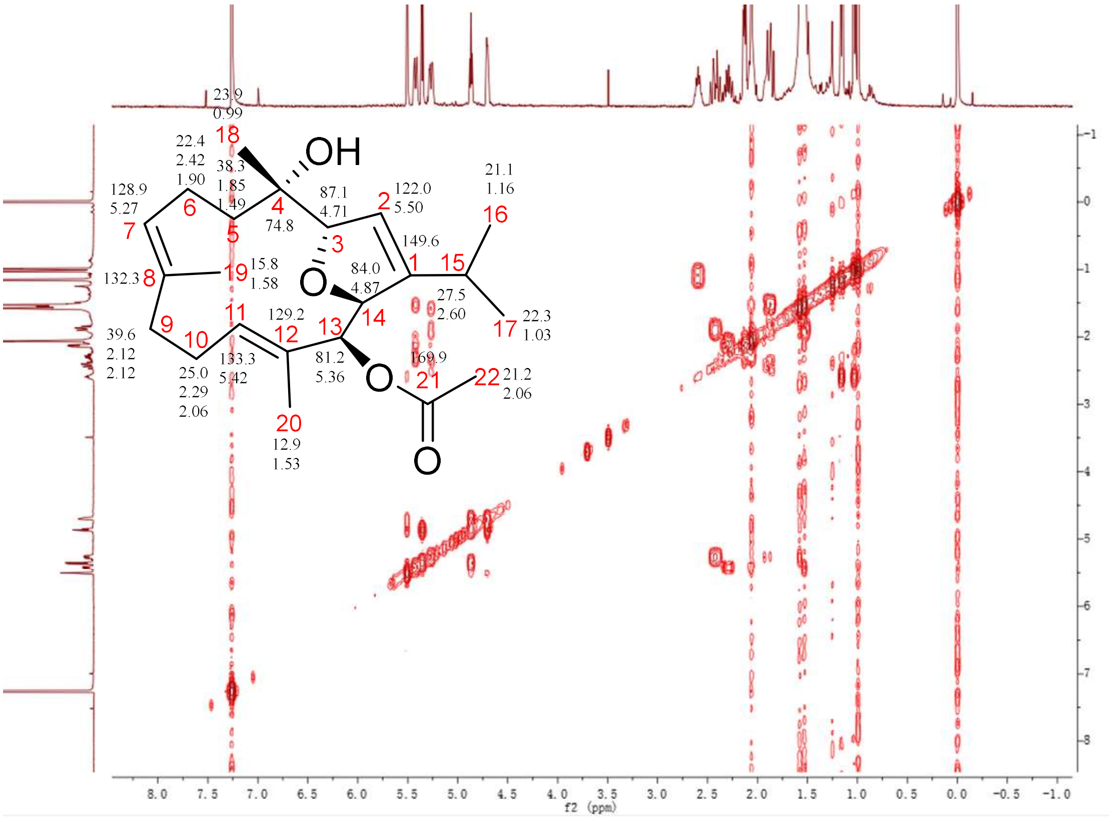

Figure S66. ^{1}H-^{1}H COSY (500 MHz, $CDCl_3$) of **6**

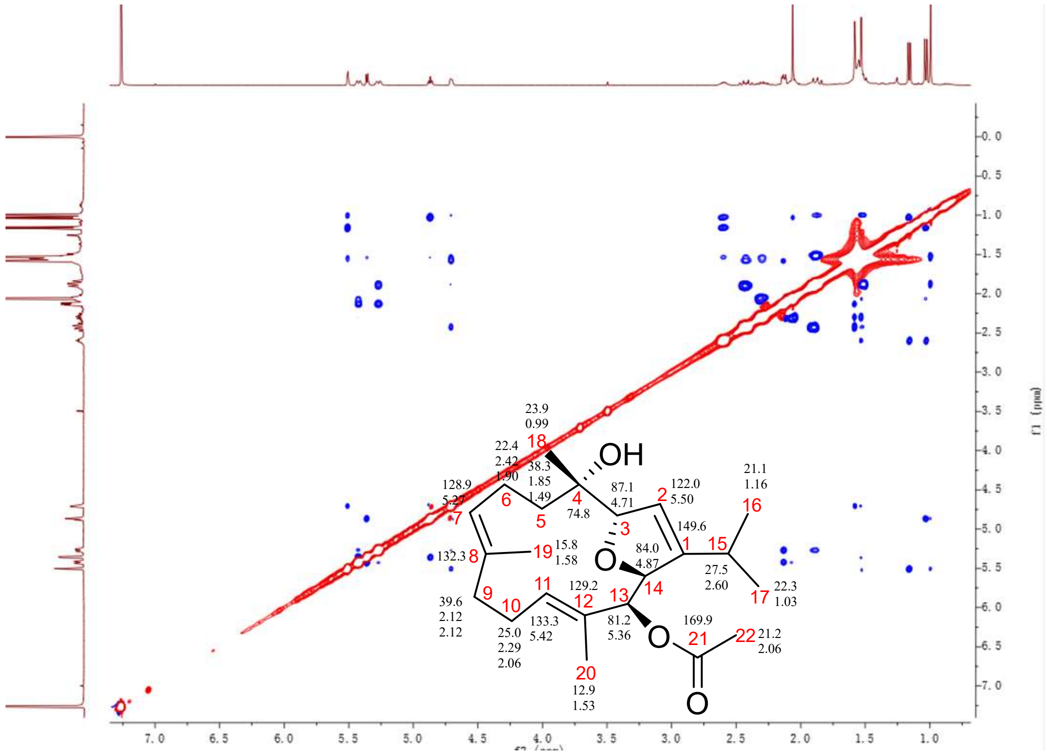

Figure S67. NOESY (500 MHz, $CDCl_3$) of **6**

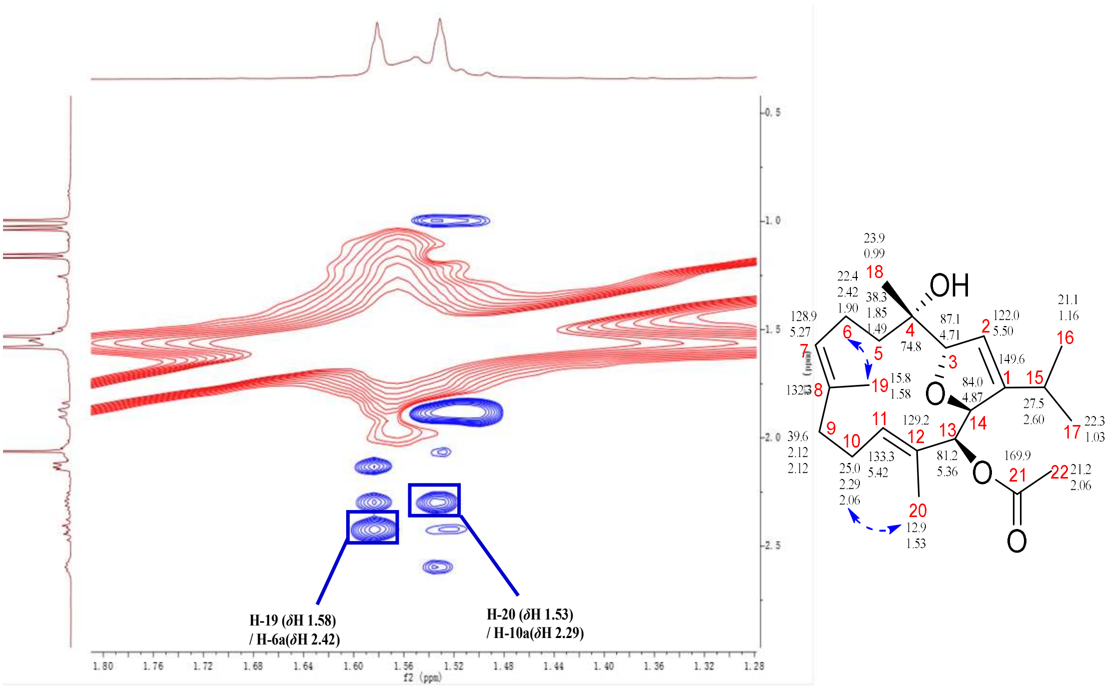

Figure S68. NOESY (500 MHz, $CDCl_3$) of **6**

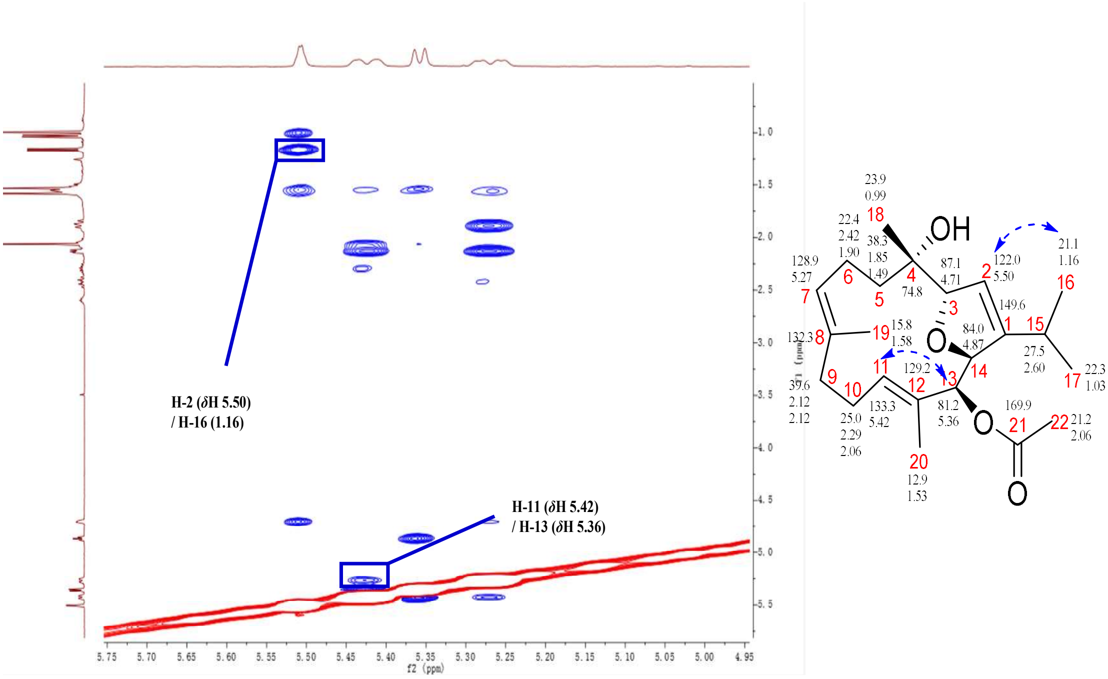

Figure S69. NOESY (500 MHz, $CDCl_3$) of **6**

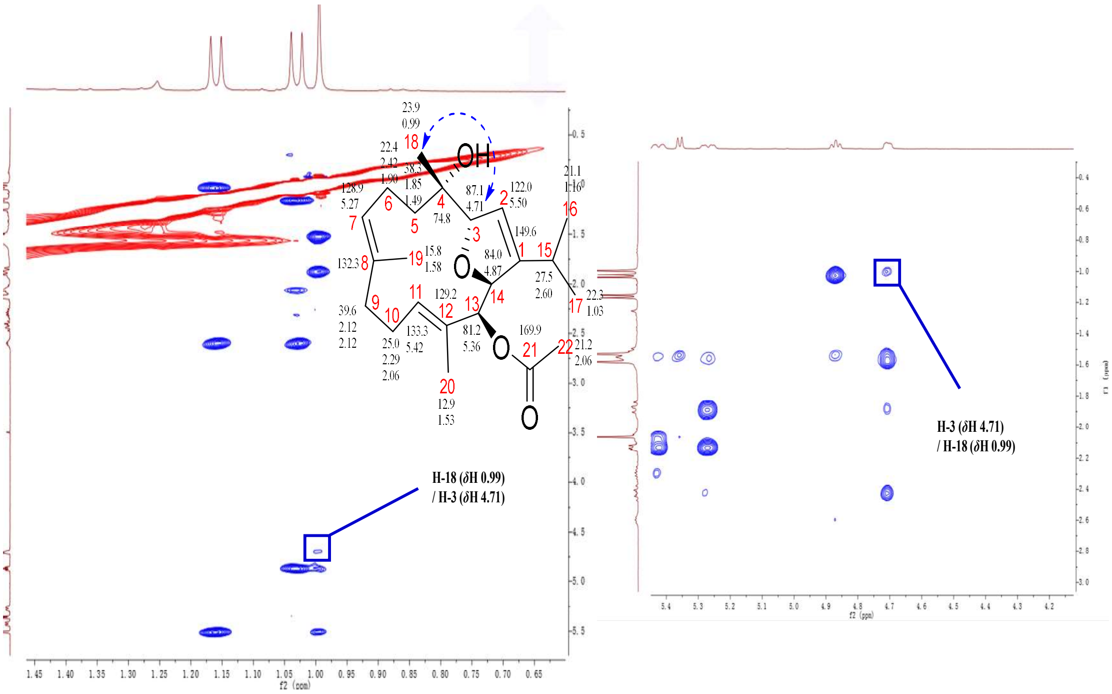

Figure S70. NOESY (500 MHz, CDCl3) of **6**

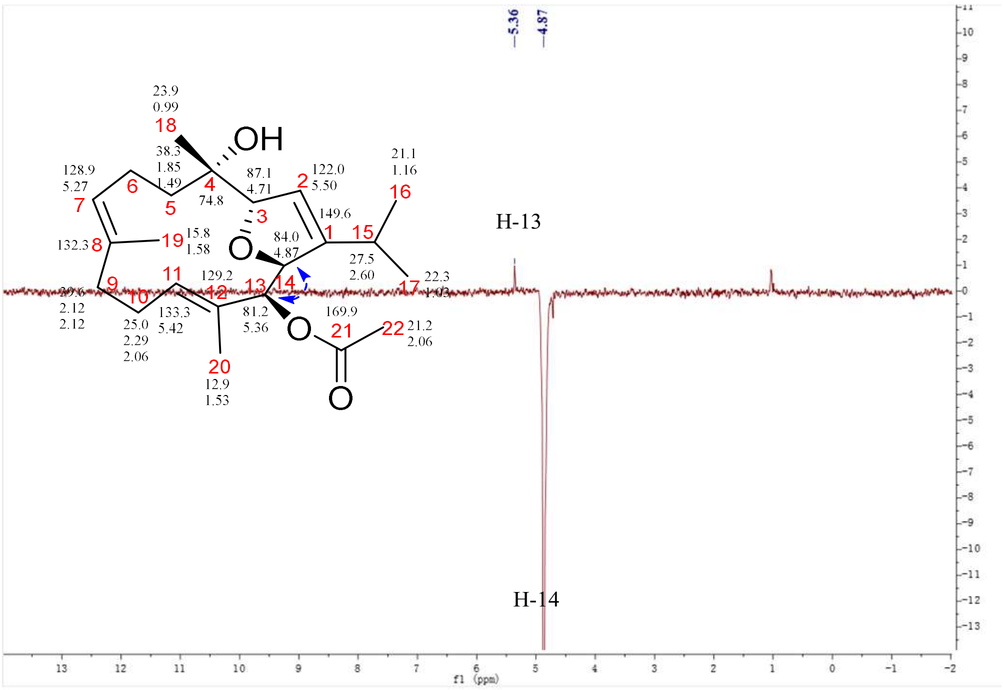

Figure S71. NOESY (500 MHz, CDCl3) of **6**

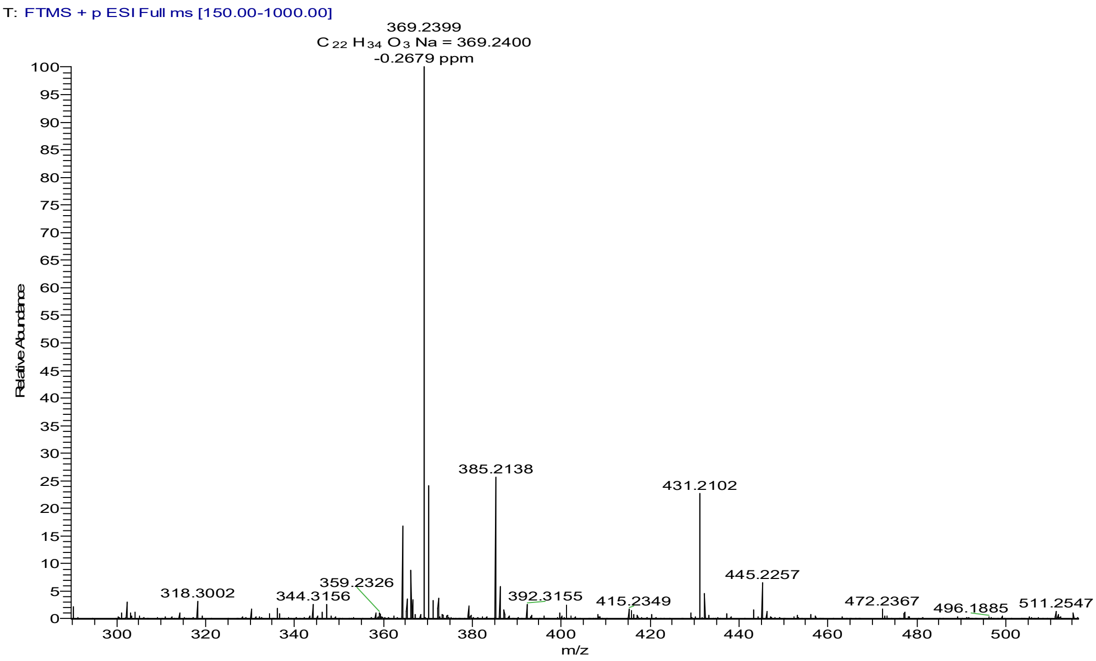

Figure S72. The positive HRESIMS spectrum of **7**

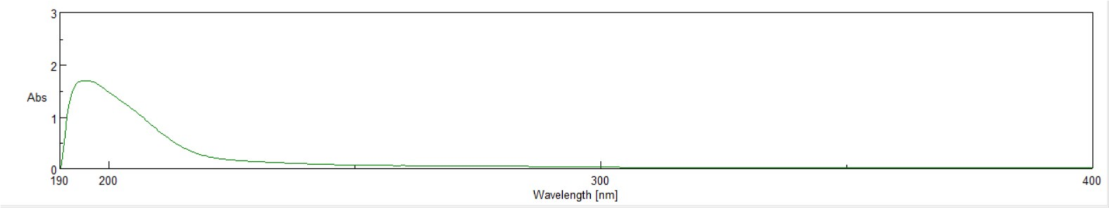

Figure S73. UV spectrum of **7**

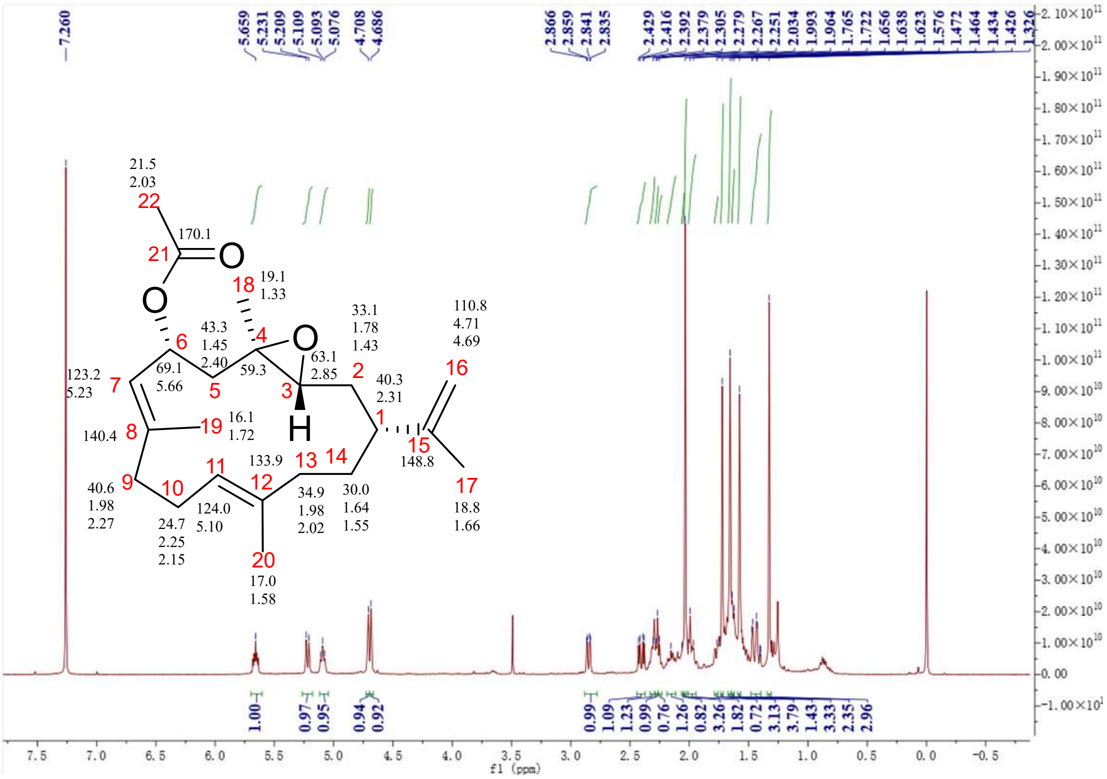

Figure S74. ^{1}H NMR spectrum (500 MHz, $CDCl_3$) of **7**

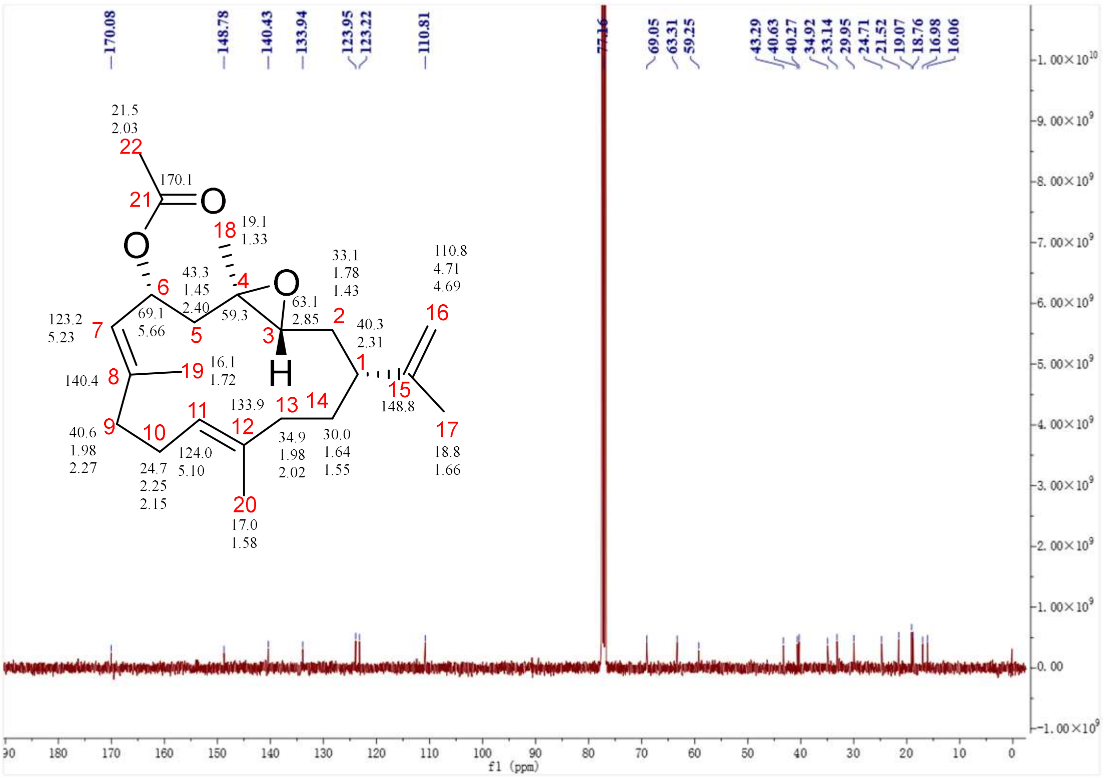

Figure S75. ^{13}C NMR spectrum (125 MHz, $CDCl_3$) of **7**

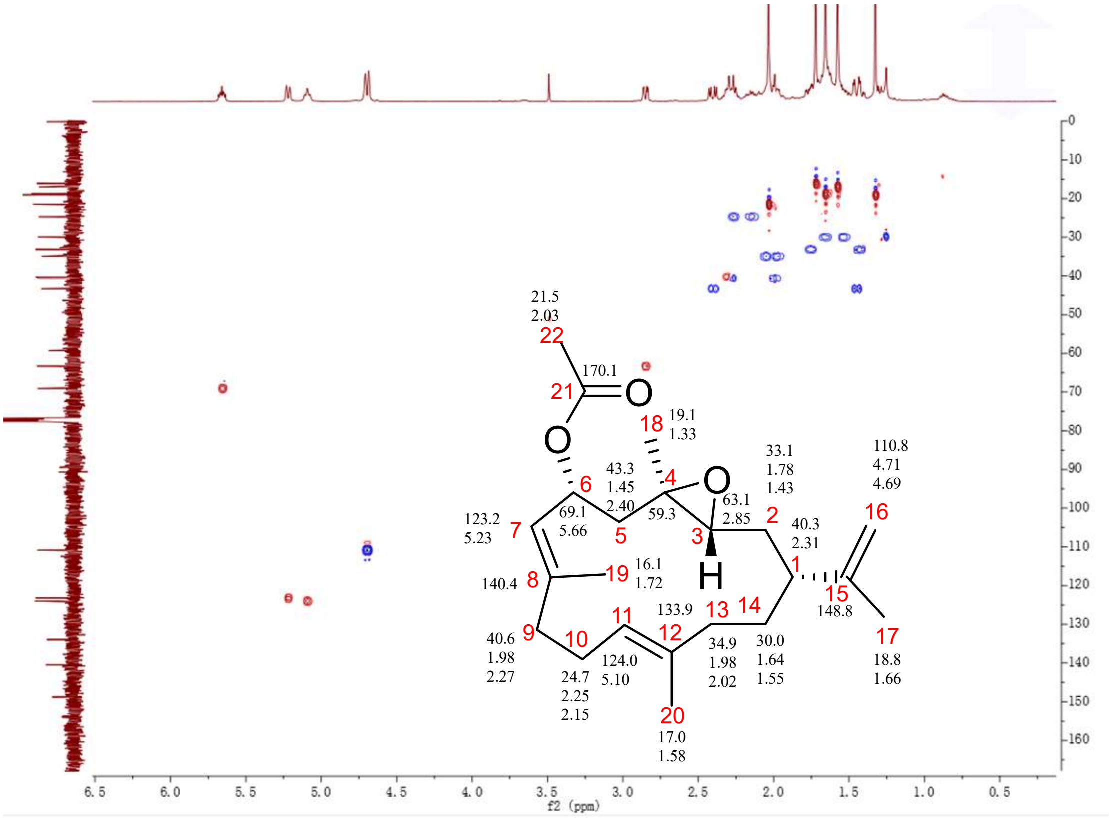

Figure S76. HSQC (500 MHz, $CDCl_3$) of **7**

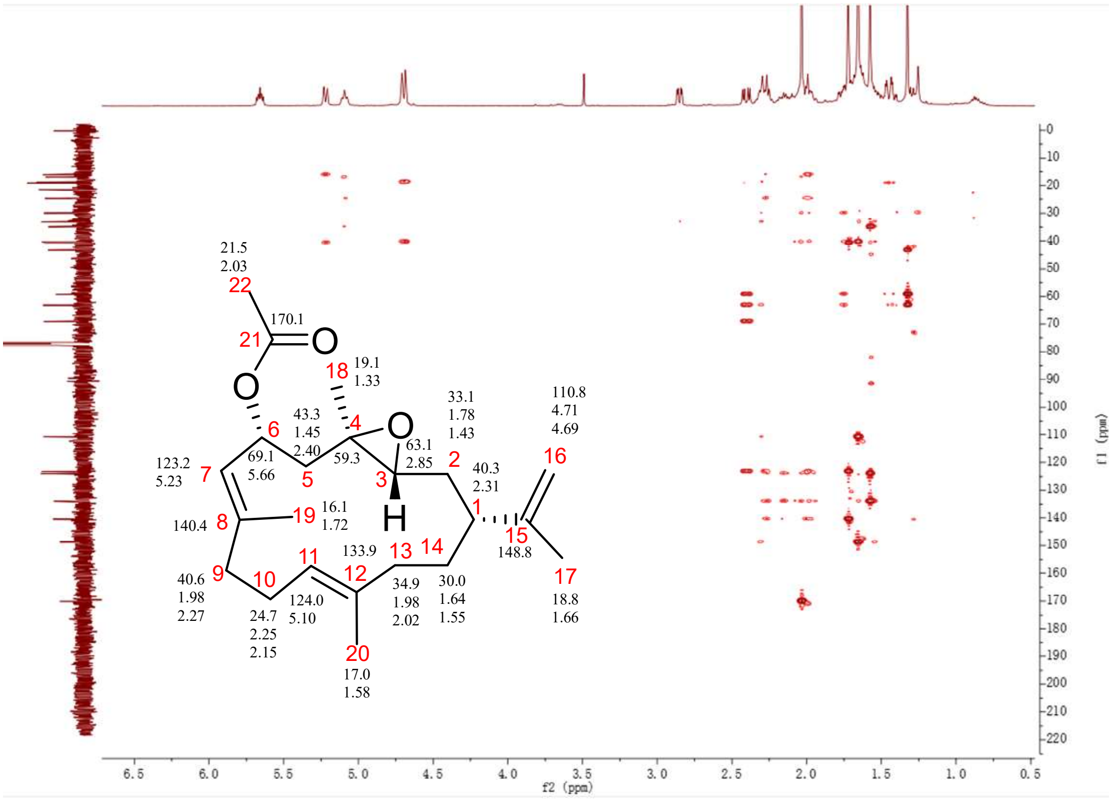

Figure S77. HMBC (125 MHz, $CDCl_3$) of **7**

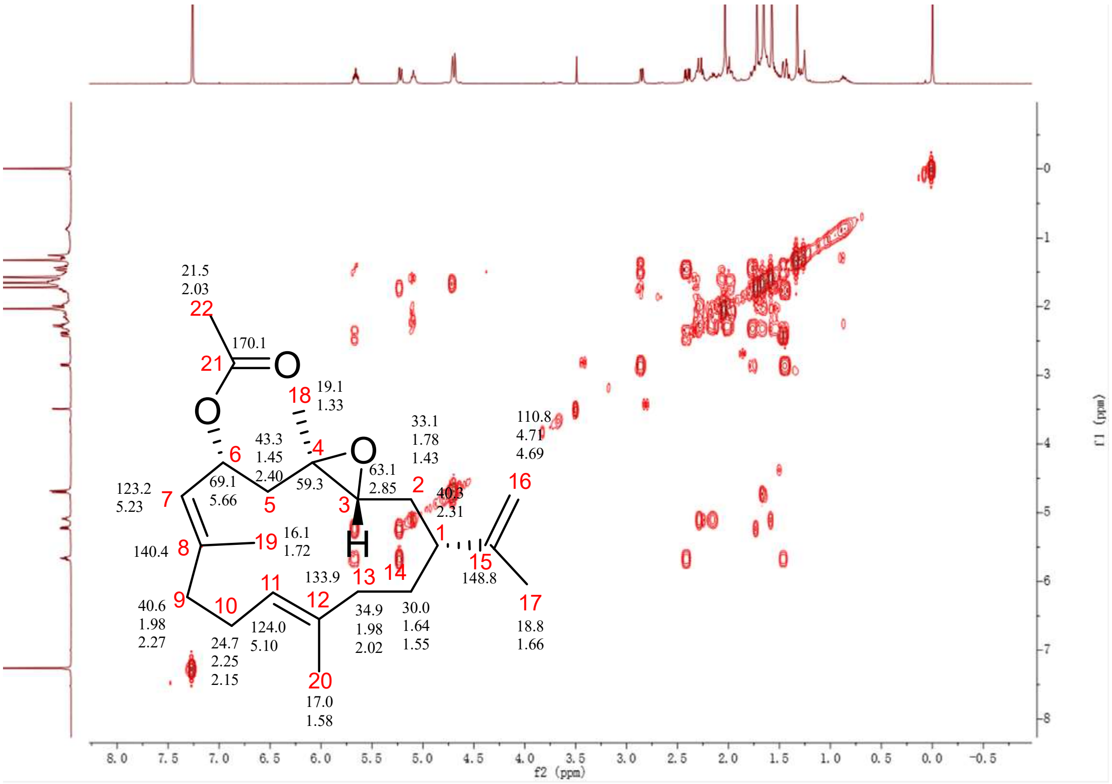

Figure 78. ^1H-^1H COSY (500 MHz, $CDCl_3$) of **7**

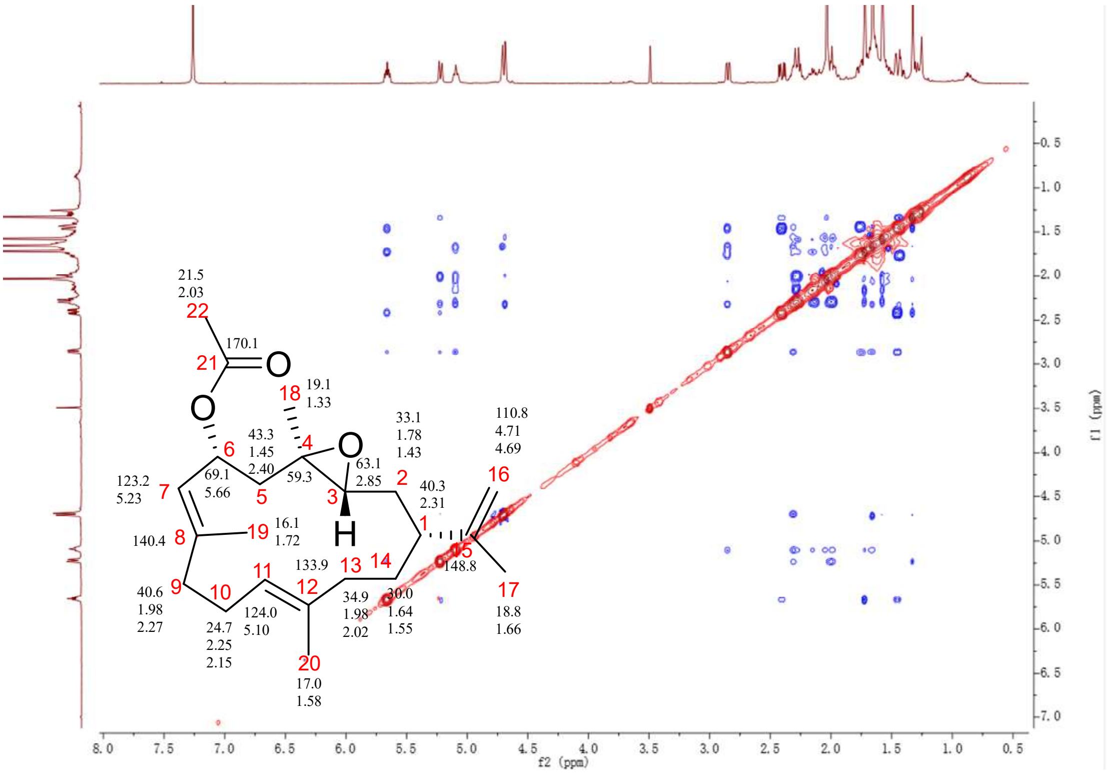

Figure S79. NOESY (500 MHz, $CDCl_3$) of **7**

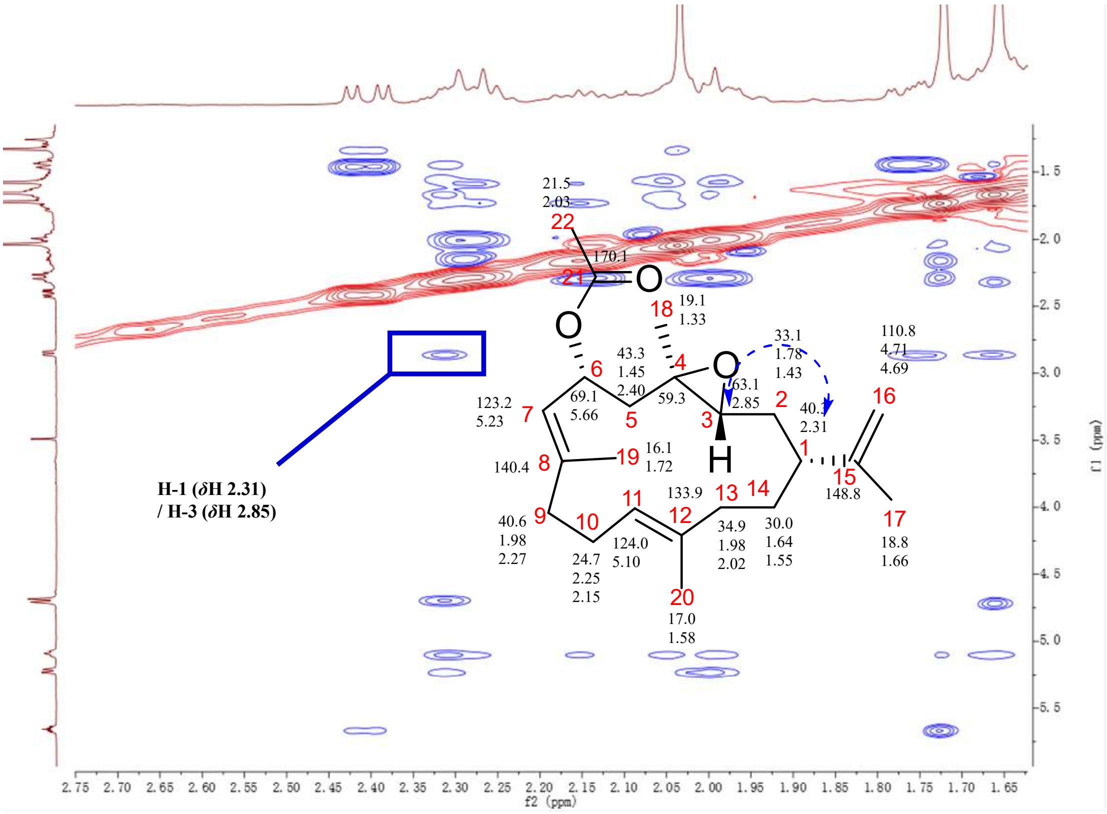

Figure S80. NOESY (500 MHz, $CDCl_3$) of **7**

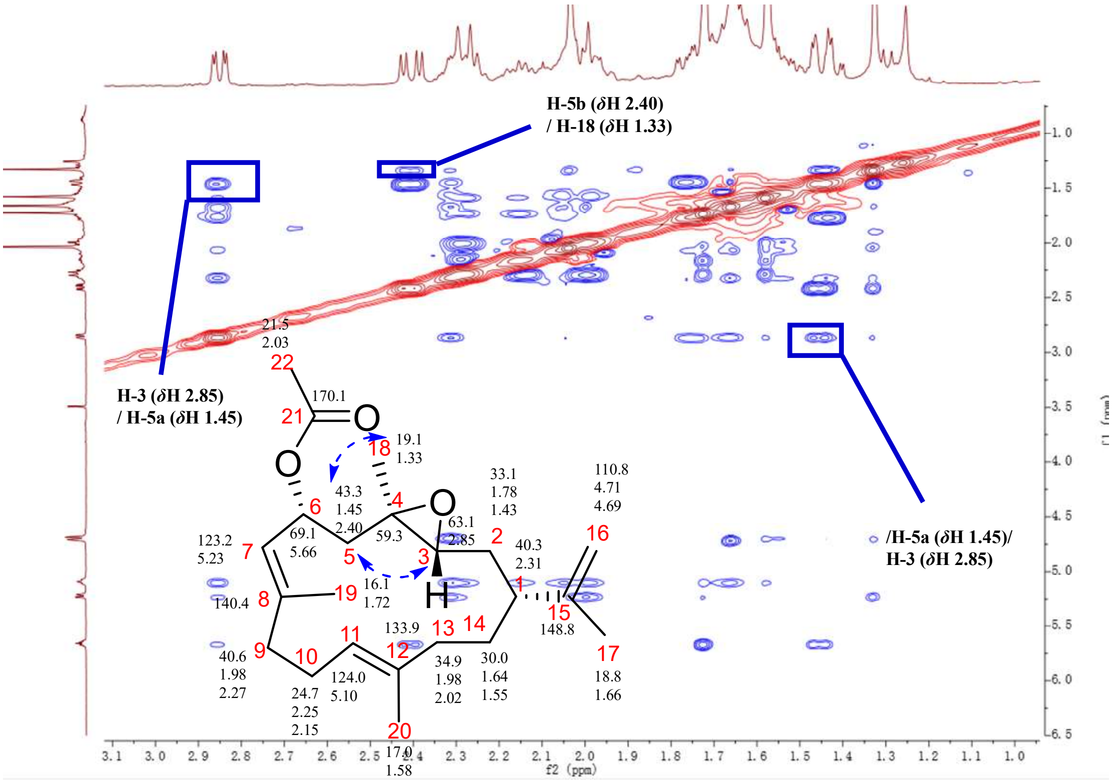

Figure S81. NOESY (500 MHz, $CDCl_3$) of **7**

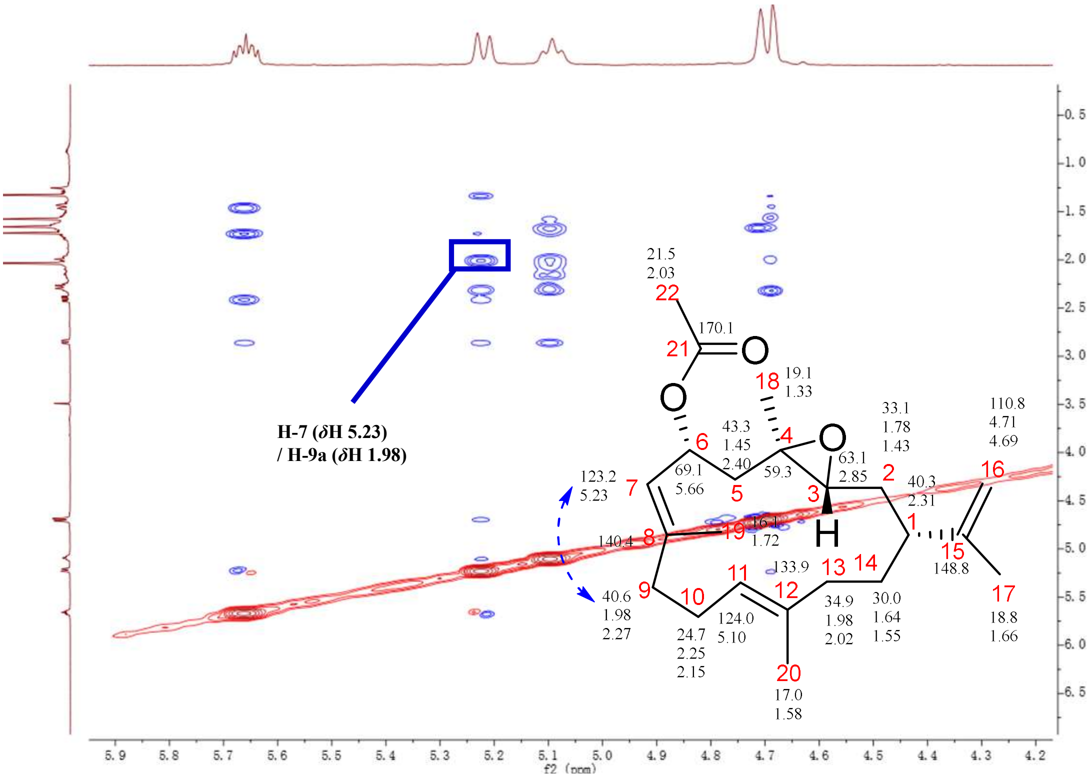

Figure S82. NOESY (500 MHz, $CDCl_3$) of **7**

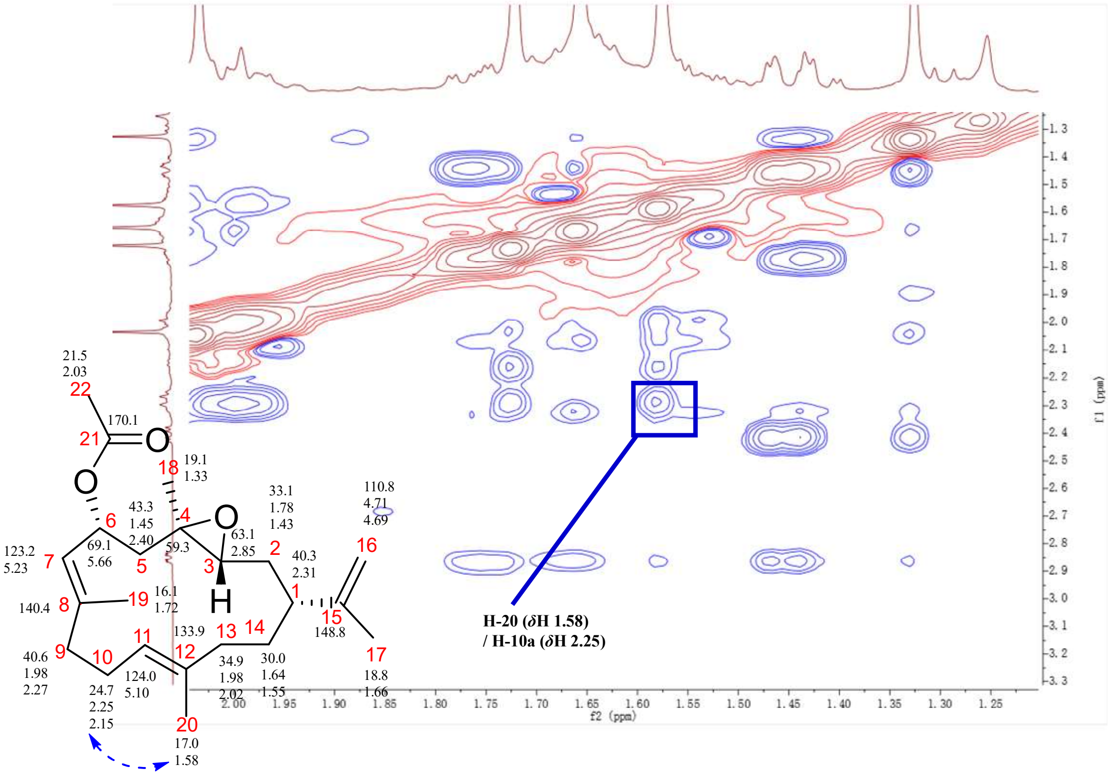

Figure S83. NOESY (500 MHz, $CDCl_3$) of **7**

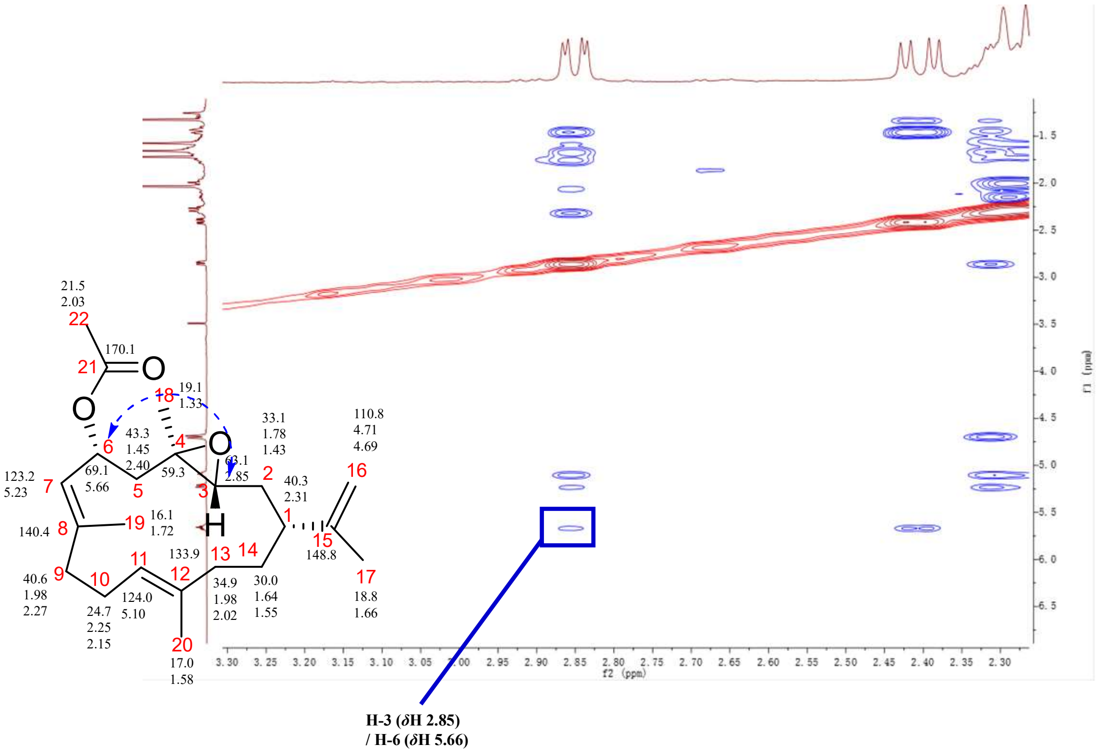

Figure S84. NOESY (500 MHz, $CDCl_3$) of **7**
